# Supplementary material for: Revealing process and material parameter effects on densification via phase-field studies
Source: Sci Rep. 2024 Mar 4;14:5350. doi: 10.1038/s41598-024-51915-w (PMC10912692; doi:10.1038/s41598-024-51915-w)
Supplement: Supplementary file 1 — Supplementary Information. [file 41598_2024_51915_MOESM1_ESM.zip › supmat/derivation.pdf]

Derivation of the sintering model used in "Revealing process and material parameter effects on densification via phase-field studies"

Marco Seiz, Henrik Hierl, Britta Nestler, Wolfgang Rheinheimer

December 10, 2023

1 Evolution equations

In this section the derivation of the evolution equations is given. In principle, it is the same as for any phase-field model using the grand potential ansatz, so the interested reader is referred to [1] for more details. The only relevant deviations will be the addition of a more complicated mobility term and advective terms in the evolution equation.

We start from a free-energy functional

$$\mathcal{F} = \int_V \epsilon a(\phi, \nabla\phi) + \frac{1}{\epsilon} w(\phi) + \psi(\phi, \mu, T) dV \tag{1}$$

which describes the total energy contained in the volume V. The phase-field vector $\phi = (\phi_0, \phi_1, \dots, \phi_{N-1})$ describes the local volume fractions of the N phases ϕ_α. For the present case of sintering, $\phi_0 = \phi_\mathrm{V}$ represents the surrounding atmosphere and $\phi_\alpha, \alpha > 0$ grains of arbitrary orientation. These phase-fields represent the local microstructure and thus their spatial distribution can be compared to experimental microstructures. The chemical potential vector $\mu = (\mu_0, \mu_1, \dots, \mu_{K-1})$ similarly describes the local chemical potential of the K components and is related to the concentration vector $c = (c_0, c_1, \dots c_{K-1})$. The local temperature is given by T. For conciseness, summations over the phase-field vector will be assumed to be from $\alpha = 0$ to $N-1$, i.e. all phases.

The gradient energy density a and the bulk energy density w are defined as

$$a(\phi, \nabla\phi) = \sum_\alpha \sum_{\alpha<\beta} \gamma_{\alpha\beta} A^\gamma(q_{\alpha\beta})^2 |q_{\alpha\beta}|^2 \tag{2}$$

$$w(\phi) = \begin{cases} \frac{16}{\pi^2} \sum_\alpha \sum_{\alpha<\beta} \gamma_{\alpha\beta}\phi_\alpha\phi_\beta + \sum_\alpha \sum_{\alpha<\beta} \sum_{\alpha<\beta<\delta} \phi_\alpha\phi_\beta\phi_\delta\gamma_{\alpha\beta\delta}, & \phi \in \mathcal{GS}^N \\ \infty, & else \end{cases} \tag{3}$$

and include contributions due to interfacial energy $\gamma_{\alpha\beta}$. The function $A^\gamma(q_{\alpha\beta})^2$ allows the modelling of anisotropy into the gradient energy density, effectively scaling the interface width depending on the orientation. The orientation is described by the generalized gradient vector $q_{\alpha\beta} = \phi_\alpha\nabla\phi_\beta - \phi_\beta\nabla\phi_\alpha$. For the present work $A^\gamma(q_{\alpha\beta})^2 = 1$ and hence isotropic behavior is assumed.

The chosen bulk energy density w is an obstacle type of potential. It consists of dual interactions terms, accounting for the two-phase interfacial profile, and a triple interaction term which reduces the appearance of so-called ghost phases along interfaces[2, 3]. The obstacle potential can reduce the computational effort when solving the phase-field equations by constricting the nontrivial phase-field values to only a narrow range called the interface. Thus computations in which the volume and its neighbours all contain the same phase-field values (called *bulk*) of either 0 or 1 can be skipped altogether, as no change is possible for these. This is allowed by forcing the phase-field vector ϕ to lie within the N-dimensional Gibbs simplex

$$\mathcal{GS}^N = \{\phi \in \mathcal{R}^N : \sum_\alpha \phi_\alpha = 1, \phi_\alpha \geq 0\} \tag{4}$$

via declaring the energy outside of this simplex to be infinite. This requires a projection step in the numerical solution, as the evolution equation derived later does not guarantee that ϕ stays in $\mathcal{GS}^N$.

The driving force for interface migration is described by

$$\psi(\phi, \mu, T) = \sum_\alpha h_\alpha(\phi)\psi_\alpha(\mu, T) \tag{5}$$

with the grand potential densities of the individual phases ψ_α and a weighting function $h_\alpha(\phi)$ with the properties $h_\alpha(0) = 0, h_\alpha(1) = 1, \frac{\mathrm{d}h_\alpha}{\mathrm{d}\phi}(\phi \in \{0, 1\}) = 0, \sum_\alpha h_\alpha = 1$. These properties ensure that the driving force for other phases β vanish within the bulk of an α phase. The function

$$h_\alpha(\phi) = \frac{\phi_\alpha^2}{\sum_\beta \phi_\beta^2} \tag{6}$$

will be employed here, based on [4]. For sintering, the driving force for interface migration should be negligible compared to capillary driving forces. Hence to a first order approximation, the precise value of ψ_α does not matter as long as it results in negligible phase transformation. For this reason we take the simple approach of modelling the Gibbs energy G_α of the respective phases as parabolas

$$G_\alpha = A_\alpha(c - c_{eq,\alpha})^2 \tag{7}$$

from which the Legendre transform to ψ_α results as

$$\psi_\alpha = -\frac{\mu^2}{4A} - c_{eq,\alpha}\mu. \tag{8}$$

As long as each phase α is initialized with a value of $c_{eq,\alpha}$ (flat equilibrium value) for its concentration, capillarity will be the main driving force.

Now that all the terms of the functional eq. (1) are known, the evolution is

given by a variational derivative plus an advective term

$$\frac{\partial \phi_\alpha}{\partial t} + \nabla \cdot (v_\alpha \phi_\alpha) = -\frac{1}{\tau(\phi, q_{\alpha\beta})\epsilon} \frac{\delta \mathcal{F}}{\delta \phi_\alpha} - \Lambda \tag{9}$$

for each phase α. The term τ can be seen as an inverse mobility and by including the orientation via $q_{\alpha\beta}$ as a parameter, a kinetic anisotropy can be included as well, but for the present work isotropic behaviour is assumed. Λ is a Lagrange multiplier as to account for the constraint $\sum_\alpha \phi_\alpha = 1$. For the obstacle potential, a one-dimensional analysis of the equilibrium profile shows that the phase-field parameter $\gamma_{\alpha\beta}$ corresponds to the interface energy $\sigma_{\alpha\beta}$ and hence no distinction is made henceforth between the two. Finally, the advective term is motivated by earlier works of the authors [5–7] which showed that for densification to occur homogeneously, advection is necessary. It should be noted that the advective term at this point in the model is not related to the functional and thus could lead to an increase in free energy and wrong dihedral angles[5, 7]. This point will be touched upon later, since first we need to couple eq. (9) to a conservative equation as to ensure conservation between the green body and its surrounding atmosphere.

The change in concentration c is given by a conservation law with fluxes due to both diffusion and advection

$$\frac{\partial c}{\partial t} = -\nabla \cdot (j_d + j_a) \tag{10}$$

$$= -\nabla \cdot (-M\nabla\mu + vc) \tag{11}$$

with the mobility M describing volume (V), surface (S) and grain boundary (GB) diffusion as[8]

$$M = M_V + M_{\alpha\beta}^{GB} + M_{\alpha \mathrm{V}}^{S} \tag{12}$$

$$M_V = \sum_\alpha \frac{\partial c_\alpha(\mu, T)}{\partial \mu} h_\alpha(\phi) D_\alpha \phi_\alpha \tag{13}$$

$$M_{\alpha \mathrm{V}}^{S} = \sum_{\alpha > \mathrm{V}} D_\alpha^S \left(\frac{\partial c_\alpha}{\partial \mu}\phi_\alpha + \frac{\partial c_\mathrm{V}}{\partial \mu}\phi_\mathrm{V}\right) I(\phi_\alpha, \phi_\mathrm{V}) \tag{14}$$

$$M_{\alpha\beta}^{GB} = \sum_{\alpha > \mathrm{V}} \sum_{\beta > \alpha} D_{\alpha\beta}^{GB} \left(\frac{\partial c_\alpha}{\partial \mu}\phi_\alpha + \frac{\partial c_\beta}{\partial \mu}\phi_\beta\right) I(\phi_\alpha, \phi_\beta) \tag{15}$$

with the generalized susceptibility $\frac{\partial c_\alpha(\mu,T)}{\partial \mu}$ and the relevant diffusion coefficients D. The function

$$I(\phi_\alpha, \phi_\mathrm{V}) = 4\phi_\alpha\phi_\beta \tag{16}$$

interpolates the interfacial diffusion across the variable phase-field. In order to decouple the interfacial energy from any bulk driving forces, the concentration is not taken to be the independent variable, but rather the chemical potential

μ. Given this inversion of relationships, the concentration follows as

$$c = -\frac{\delta \mathcal{F}}{\delta \mu} \tag{17}$$

$$c = -\frac{\partial \psi(\phi, \mu, T)}{\partial \mu} \tag{18}$$

$$c = -\sum_{\alpha} \frac{\partial \psi_{\alpha}(\mu, T)}{\partial \mu} h_{\alpha}(\phi) \tag{19}$$

$$c = \sum_{\alpha} c_{\alpha}(\mu, T) h_{\alpha}(\phi) \tag{20}$$

in which the thermodynamic relation $c_{\alpha} = -\frac{\partial \psi_{\alpha}}{\partial \mu}$ is exploited to arrive at the phase-specific concentration $c_{\alpha}(\mu, T)$. Taking the time derivative of eq. (20) yields

$$\frac{\partial c}{\partial t} = \sum_{\alpha} \frac{\partial h_{\alpha}(\phi)}{\partial t} c_{\alpha}(\mu, T) + \sum_{\alpha} h_{\alpha}(\phi) \frac{\partial c_{\alpha}(\mu, T)}{\partial t} \tag{21}$$

$$= \sum_{\alpha} \frac{\partial h_{\alpha}(\phi)}{\partial t} c_{\alpha}(\mu, T) + \sum_{\alpha} h_{\alpha}(\phi) \left[\frac{\partial c_{\alpha}(\mu, T)}{\partial \mu} \frac{\partial \mu}{\partial t} + \frac{\partial c_{\alpha}(\mu, T)}{\partial T} \frac{\partial T}{\partial t} \right] \tag{22}$$

which we can equate to the flux conservation law eq. (11) and solve for the time evolution of μ:

$$\frac{\partial \mu}{\partial t} = \left[\sum_{\alpha} h_{\alpha}(\phi) \left(\frac{\partial c_{\alpha}(\mu, T)}{\partial \mu} \right) \right]^{-1} \tag{23}$$
$$\left(-\nabla \cdot \Big(-M \nabla \mu + v c \Big) - \sum_{\alpha} c_{\alpha}(\mu, T) \frac{\partial h_{\alpha}(\phi)}{\partial t} - \sum_{\alpha} h_{\alpha}(\phi) \frac{\partial c_{\alpha}(\mu, T)}{\partial T} \frac{\partial T}{\partial t} \right).$$

Under the assumption of constant temperature T, the time derivative of T vanishes and we are left with

$$\frac{\partial \mu}{\partial t} = \left[\sum_{\alpha} h_{\alpha}(\phi) \left(\frac{\partial c_{\alpha}(\mu, T)}{\partial \mu} \right) \right]^{-1} \tag{24}$$
$$\left(-\nabla \cdot \Big(-M \nabla \mu + v c \Big) - \sum_{\alpha} c_{\alpha}(\mu, T) \frac{\partial h_{\alpha}(\phi)}{\partial t} \right).$$

Note that if the phase concentrations c_{α} are independent of temperature, as is the case for stoichiometric compounds, then T need not be assumed constant for the term to vanish. Since the Gibbs free energies assumed here are independent of temperature, c_{α} is so as well. The effect of a variable temperature would thus be restricted to the materials parameters $\gamma_{\alpha\beta}$, $\tau_{\alpha\beta}$ and the diffusivities.

2 Velocity modelling

Now that the evolution equations are known, we need a method for determining the velocity field v with the following properties:

- the free energy is monotonically reduced
- a homogeneous microstructure under isothermal conditions densifies homogeneously

The interested reader is referred to [5–7] for the details of why these conditions are required and how models based on [9] fail to account for these conditions. The short summary consists of two points: First, the potential used for the force and velocity calculation does not have a connection to the energy functional. Thus two different energy minima are sought by the system, one to eliminate the force and one to minimize the energy given by the energy functional. This leads to effects such as the wrong dihedral angle and "unshrinkage", in which the sample lengthens while trying to eliminate the force. Second, calculating grain velocity from nearest-neighbour interactions only fails to account for the actual global coupling between all grains, which happens at much faster timescales than the diffusive one. This effectively only allows densification for grains which have missing neighbours, whereas those in an "isotropic" neighbourhood do not experience strain w.r.t. their immediate neighbours. The mathematics of this are shown in greater detail in section 3.

In order to build a model for these properties, we start from a Gedankenexperiment: Consider a GB not in mechanical equilibrium with its adjacent grains, and for simplicity say that it only differs in pressure to these. It can change its state by absorbing and emitting vacancies, which changes its volume and hence pressure, until equilibrium is obtained. The volume change dV is given by

$$dV = \Omega dN \tag{25}$$

with the vacancy volume Ω and the number of emitted vacancies dN, with a negative sign for absorbed vacancies. This volume change also causes the adjacent grains to move towards the GB by a displacement element

$$du = -\frac{dV}{A} \tag{26}$$

where A is the grain boundary area, with the negative sign causing a shrinking GB to attract the grains into its reduced volume. Note that both grains move by $\frac{|du|}{2}$, but with differing signs towards the GB. The relative velocity element then follows simply by division over the observed time interval dt:

$$dv = \frac{du}{dt} \tag{27}$$

$$dv = -\frac{\Omega dN}{A dt} \tag{28}$$

with a volume integral of the GB giving the relative grain velocities / displacement jumps as

$$\Delta v = -\int_{GB} \frac{\Omega dN}{A dt} dV \tag{29}$$

$$\Delta u = -\int_{GB} \frac{\Omega dN}{A} dV. \tag{30}$$

Note that for ease of notation, the negative signs are omitted in the main paper since dN is taken to be the number of absorbed vacancies.

Based on this derivation, we still need to ask two questions:

- How is the displacement transported across the rest of the grain structure?
- How is the number of emitted vacancies dN determined?

The first question can be answered by considering that du also describes the displacement jump Δu between the grains. Assuming that these displacement jumps can be superimposed, with justification given in [7], one can write

$$\mathrm{C}u = \Delta u \tag{31}$$

wherein the contact matrix C describes how grains are connected. The resulting displacements u do not necessarily conserve momentum, which can be ensured by requiring $\sum_\alpha u_\alpha V_\alpha = 0$ over all grains α with their respective volume V_α. Note that the strain between two particles is independent of this requirement; the green body as a whole would simply undergo a rigid-body translation in space if the condition is not enforced.

The structure of C is most readily explained by a simple example of a two-by-two grid of particles, as shown in Fig. 1 and described in greater detail in [7]. In each dimension a particle has two contacts. The contact is taken to be from the lower grain index α to the higher one, i.e. the ordered set $\{C_{1,2}, C_{1,3}, C_{2,4}, C_{3,4}\}$ describes the contacts completely. A contact matrix C_d is now constructed for each dimension by the following procedure: For each contact, the matrix gains a row with as many columns as there are grains, with the only non-zero entries being given by the grain indices. The entries' magnitude is 1, but the sign is consistent with the grain boundary normal pointing from the lower to the higher grain index. If the grain boundary normal in this direction is zero, then arbitrary signs can be used, as no displacement jump will occur in this direction[1]. Thus for the contact matrix in the x dimension we have

$$\begin{pmatrix} 1 & -1 & 0 & 0 \\ 1 & 0 & -1 & 0 \\ 0 & 1 & 0 & -1 \\ 0 & 0 & 1 & -1 \end{pmatrix}.$$

The matrix equation fulfills the homogeneity requirement, analytically shown for a grain chain in section 3 and experimentally in [7].

Now we only need to approximate the number of emitted vacancies dN. This couples back to the question of free energy, as this process must stop at the same equilibrium as predicted by the energy functional. For this, we assume that the chemical potential on the surface μ_s gives a good approximation of the equilibrium state and hence the value of μ which ought to be found in the grain boundary. It is determined by

$$\mu_{s,\alpha} = \frac{\int 4\phi_\alpha \phi_\mathrm{V} \mu dV}{\int 4\phi_\alpha \phi_\mathrm{V} dV} \tag{32}$$

[1]This ignores for example grain boundary sliding.

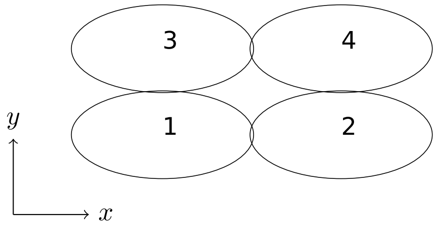

Figure 1: Example two-by-two setup of grains for clarifying the matrix structure, based on [7]. The number within the circles indicates the grain index.

i.e. a weighted average of μ on the surface, since $\phi_\alpha \phi_\mathrm{V}$ is nonzero only on the surface. Since this value ought to be shared by both grains across a grain boundary, the average over both observed μ_s is taken:

$$\mu_{s,\alpha\beta} = \frac{\mu_{s,\alpha} + \mu_{s,\beta}}{2}. \tag{33}$$

With this, a relaxation ansatz holding in the GB region and of the form

$$\frac{\partial c_{\alpha\beta}^{GB}}{\partial t} = -\frac{c - c_{eq}^{GB}(S)}{t_r} \tag{34}$$

$$\Delta c_{\alpha\beta}^{GB} = -\frac{c - c_{eq}^{GB}(S)}{t_r} \Delta t \tag{35}$$

can be written, wherein the dependence on the state S gives a relationship between the concentration on the GB and $\mu_{s,\alpha\beta}$. Note that the concentration c can be converted into a number density n, from which the absolute number of vacancies N follows as

$$n = \frac{N_a}{V_m} c \tag{36}$$

$$N = V_r n \tag{37}$$

with Avogadro's constant N_a, the molar volume V_m and the reference volume V_r in which the vacancy absorption/emission process is assumed to be homogeneous. Inserting these relations in eq. (30) yields

$$\Delta u = -\int_{GB} \frac{\Omega}{A} \frac{N_a}{V_m} \Delta c^{GB} dV \tag{38}$$

which needs to take into account the GB orientation $\vec{n}_{\alpha\beta}$

$$\Delta \vec{u}_{\alpha\beta} = -\frac{1}{V_{\alpha\beta}} \int_{GB} 4\phi_\alpha \phi_\beta \frac{\Omega}{A_{\alpha\beta}} \frac{N_a}{V_m} \Delta c_{\alpha\beta}^{GB} \vec{n}_{\alpha\beta} dV \tag{39}$$

$$V_{\alpha\beta} = \int_{GB} 4\phi_\alpha \phi_\beta dV \tag{40}$$

in which an additional weighting by the grain boundary $4\phi_\alpha\phi_\beta$ is introduced and the dependence on the phases made explicit. The grain boundary region

GB is defined as the region in which $\phi_\alpha \phi_\beta > \phi_{\alpha\beta}^{min} = 0.14$.

The dependence on the state S can be resolved via $c_\alpha = -\frac{\partial \psi_\alpha}{\partial \mu}$ under the assumption that the GB has approximately the same concentration as the bulk grain in equilibrium:

$$c_{eq}^{GB}(S) \approx c_\alpha(\mu_{s,\alpha\beta}) = \frac{\mu_{s,\alpha\beta}}{2A} + c_{eq,\alpha} \tag{41}$$

wherein the parabolic approximation eq. (7) was used. A different concentration could be assumed for the GB as in [10] to account for the experimentally observed variance of density across GBs. This mainly couples back to the form of the free energies which is used, with the rest of derivation staying as-is once the new functional form of the free energy is determined.

Since this ansatz depends on the system state, analytically showing that the free energy is reduced monotonically is nontrivial[2]. It is easy to see however that the equilibrium state is unchanged by this ansatz, since the chemical potential on a particle's surface is independent of how it moves as a rigid body through space. Hence the observed μ_s in equilibrium, with or without advection, will be the same. Together with the necessary condition of $\mu = \mu_s$ everywhere for equilibrium, the same equilibrium state follows.

3 Homogeneity of strain

Consider a chain of N equally-sized cuboid grains of individual length l and constant grain boundary area A. Assume that each of the $B = N - 1$ GBs has just absorbed ΔN vacancies, which induces a displacement jump Δu across each GB. By the superposition property, the solution is the sum of all solutions only dealing with a single GB. Thus without loss of generality, assume that only the first GB absorbed vacancies. This results in the system

$$u_1 - u_2 = \Delta u \tag{42}$$

$$\sum_i u_i = 0 \tag{43}$$

with conservation of momentum employing the equal grain size assumption. The first equation directly resolves to $u_2 = u_1 - \Delta u$. Since there is no displacement jump between grains 2 and 3, 3 and 4 and so on, we must necessarily have

$$u_2 = u_3 = u_4 = \ldots = u_1 - \Delta u \tag{44}$$

[2]Except for the case of a dense polycrystal, since then $\mu_s = \mu_{eq} = 0$ as no surface is present.

and inserting this into conservation of momentum yields

$$u_1 + (N-1)(u_1 - \Delta u) = 0 \tag{45}$$
$$Nu_1 = (N-1)\Delta u \tag{46}$$
$$u_1 = \frac{N-1}{N}\Delta u \tag{47}$$
$$u_2 = (\frac{N-1}{N} - 1)\Delta u \tag{48}$$

This procedure can easily be repeated for every grain boundary yielding a similar expression for the grain displacement. The displacements will always be weighted by how many grains are to the left and to the right of the grain boundary[3] absorbing vacancies, i.e.

$$u_i = \frac{N-i}{N}\Delta u \tag{49}$$
$$u_{i+1} = (\frac{N-i}{N} - 1)\Delta u \tag{50}$$

with all grains left of grain i exhibiting the same displacement as grain i and v.v. for grains right of grain $i+1$. The superposition property now lets us sum over all these problems to arrive at the solution to our original problem. For simplicity, we only consider the leftmost grain

$$u_1^{SP} = \sum_{i=1}^{N-1} \frac{N-i}{N}\Delta u \tag{51}$$
$$= \frac{\Delta u}{N}\sum_{i=1}^{N-1} N-i \tag{52}$$
$$= \frac{\Delta u}{N}\left[N(N-1) - \frac{(N-1)N}{2}\right] \tag{53}$$
$$= \frac{\Delta u(N-1)}{2} \tag{54}$$

and note that there are only $N-1$ GBs and thus limit the sum to $N-1$. As a sanity check, consider $N=2$ in which the above formula yields $u_1^{SP} = \frac{\Delta u}{2}$ which is also the result of solving eqs. (42) and (43) by hand. It is entirely sufficient to only consider the leftmost grain since the displacement field should be point symmetric w.r.t. the total center of mass (conservation of momentum) and thus $u_N^{SP} = -u_1^{SP}$. Furthermore, the total length change of the chain is simply $u_1^{SP} - u_N^{SP} = \Delta u(N-1)$. Hence we have with the definition of strain

$$e = \frac{\Delta L}{L} \tag{55}$$
$$= \frac{\Delta u}{l}\frac{N-1}{N} \tag{56}$$

which converges to a constant ($\frac{\Delta u}{l}$) as N goes to infinity.

[3]In effect the grains with non-absorbing boundaries can be lumped together with their adjacent, absorbing grain.

This would imply some finite dependence of the strain on the system size (here number of grains N) as a whole. However, each contact experiences the same strain $\frac{\Delta u}{l}$. The apparent non-convergence at low N results from the choice of end-to-end distance as the reference length. If the reference length is taken to be measured between center of masses, then N cancels out and constant strain independent of system size is achieved.

We can also take a similar approach for showing why taking into account nearest neighbour interactions only, i.e. a simple resultant force expression, fails to reach homogeneous strain. Trivially, if the same force occurs on each GB, all but the two outermost particles will not move. This does cause a change in the force distribution, spreading at the speed of one grain size per time step. This change, however, will arrive at different GBs at different times and hence for sufficiently large structures, the changes will become decorrelated and hence result in strain mostly occurring on the outside of the structure, as shown in [6]. A more magnanimous comparison might be asking what kind of force distribution is necessary to produce a linear profile in displacement and hence constant strain? Based on [9], ignoring multiplicative factors, each grain's displacement is given by

$$u_\alpha = v_\alpha dt \tag{57}$$

$$= \sum_{\beta \neq \alpha} F_{\alpha,\beta} \tag{58}$$

$$= F_{\alpha+1,\alpha} - F_{\alpha,\alpha-1} \tag{59}$$

in which the same chain geometry as before is assumed to resolve the summation. The forces F are taken to be located on the GB, with the index pair α, β uniquely identifying the GB. Forces on a grain α are taken to be positive if the first index is larger than α, negative otherwise. Now we would simply like to have

$$u_{\alpha+1} - u_\alpha = \Delta u \tag{60}$$

$$= F_{\alpha+2,\alpha+1} - F_{\alpha+1,\alpha} - (F_{\alpha+1,\alpha} - F_{\alpha,\alpha-1}) \tag{61}$$

$$= F_{\alpha+2,\alpha+1} + F_{\alpha,\alpha-1} - 2F_{\alpha+1,\alpha} \tag{62}$$

to be constant for any α, since then we recover the same problem as solved earlier[4]. Now say F is given by a continuous function of space x, sharply concentrated at the GBs positioned at $x + nl, n \in \mathbb{Z}$, then we would like to have

$$f(x+l) + f(x-l) - 2f(x) = const. \tag{63}$$

in order for eq. (62) to be constant. This can be interpreted as a finite difference for the second derivative of f and hence f ought to be at most parabolic[5]. Now the physical origin of F becomes relevant: If F describes the force of springs connecting the grains, as it effectively does in [9], then the outer GBs will need to experience a greater displacement[6] out of equilibrium than the inner GBs, in order for F to be parabolically distributed. This would imply that the outer GBs

[4]Except for the outermost particles, but assume a large enough chain that it does not matter.

[5]Lower order solutions apply as well; the solution $f(x) = const.$ does not move the inner particles and for a linear f the same argument as for the parabolic solution holds.

[6]Within the model of [9], a greater density difference at their GBs.

are further from their equilibrium than the inner GBs, which is not justifiable without recourse to external factors such as a temperature distribution. If on the other hand F results from accumulated pressure differences, as is likely the case in viscous sintering, then an almost physically consistent picture is obtained. Almost, because F would not be concentrated in the absent GBs in viscous sintering, but rather it would be a true continuous function (describing pressure) and imply a continuous change in velocity, making the rigid-body assumption untenable.

References

[1] M. Plapp. Unified derivation of phase-field models for alloy solidification from a grand-potential functional. *Physical Review E*, 84(3):031601, 2011.

[2] Johannes Hötzer, Oleg Tschukin, Marouen Ben Said, Marco Berghoff, Marcus Jainta, Georges Barthelemy, Nikolay Smorchkov, Daniel Schneider, Michael Selzer, and Britta Nestler. Calibration of a multi-phase field model with quantitative angle measurement. *Journal of Materials Science*, 51(4):1788–1797, 2015.

[3] Simon Daubner, Paul W. Hoffrogge, Martin Minar, and Britta Nestler. Triple junction benchmark for multiphase-field and multi-order parameter models. *Computational Materials Science*, 219:111995, February 2023.

[4] N. Moelans. A quantitative and thermodynamically consistent phase-field interpolation function for multi-phase systems. *Acta Materialia*, 59(3):1077–1086, 2011.

[5] Marco Seiz. Effect of rigid body motion in phase-field models of solid-state sintering. *Computational Materials Science*, 215, 2022. Publisher: Elsevier B.V.

[6] Marco Seiz, Henrik Hierl, and Britta Nestler. An improved grand-potential phase-field model of solid-state sintering for many particles. *Modelling and Simulation in Materials Science and Engineering*, 31(5):055006, July 2023.

[7] Marco Seiz, Henrik Hierl, and Britta Nestler. Unravelling densification during sintering by multiscale modelling of grain motion. *Journal of Materials Science*, 58(35):14051–14071, Sep 2023.

[8] Johannes Hötzer, Marco Seiz, Michael Kellner, Wolfgang Rheinheimer, and Britta Nestler. Phase-field simulation of solid state sintering. *Acta Materialia*, 164:184–195, 2019.

[9] Yu U. Wang. Computer modeling and simulation of solid-state sintering: A phase field approach. *Acta Materialia*, 54(4):953–961, 2006.

[10] Ian Greenquist, Michael R Tonks, Larry K Aagesen, and Yongfeng Zhang. Development of a microstructural grand potential-based sintering model. *Computational Materials Science*, 172, 2020.
