# Supplementary material for: Revealing process and material parameter effects on densification via phase-field studies
Source: Sci Rep. 2024 Mar 4;14:5350. doi: 10.1038/s41598-024-51915-w (PMC10912692; doi:10.1038/s41598-024-51915-w)
Supplement: Supplementary file 1 — Supplementary Information. [file 41598_2024_51915_MOESM1_ESM.zip › supmat/moments.pdf]

Supplementary material to "Revealing process and material parameter effects on densification via phase-field studies": Individual moment invariants

Marco Seiz[1,2*], Henrik Hierl[2], Britta Nestler[1,2,3], and Wolfgang Rheinheimer[4]

[1]Institute for Applied Materials, Karlsruhe Institute of Technology, Straße am Forum 7, 76131 Karlsruhe, Germany
[2]Institute of Nanotechnology, Karlsruhe Institute of Technology, Hermann-von-Helmholtz-Platz 1, 76344 Eggenstein-Leopoldshafen, Germany
[3]Institute for Digital Materials, Karlsruhe University of Applied Sciences, Moltkestr. 30, 76133 Karlsruhe, Germany
[4]Institute for Manufacturing Technology of Ceramic Components and Composites, University of Stuttgart, Allmandring 7B, 70569 Stuttgart, Germany
[*]corresponding author: marco.seiz@kit.edu

September 15, 2023

The individual moment invariants $\widetilde{\Omega}$ are shown here for each of the defined domains, i.e. grains, isolated pores and detached pores. The invariants $\widetilde{\Omega_1}$ and $\widetilde{\Omega_2}$ are invariant under similarity transforms, i.e. the same shape but under arbitrary isotropic scaling, translation, rotation and reflection. In contrast $\widetilde{\Omega_3}$ is invariant under affine transforms, i.e. arbitrary anisotropic scaling and shearing as well as translation, rotation and reflection.

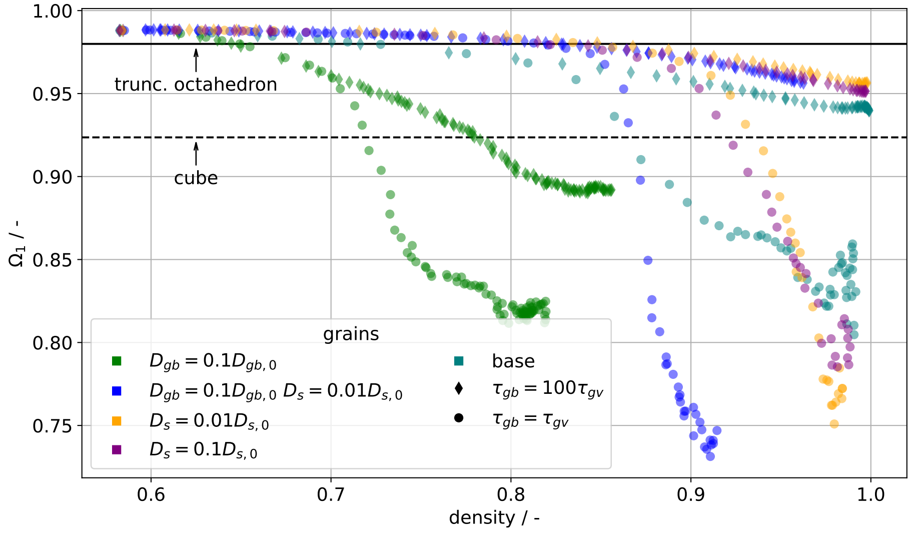

Figure 1: The moment invariant $\widetilde{\Omega_1}$ for the grain domain is depicted.

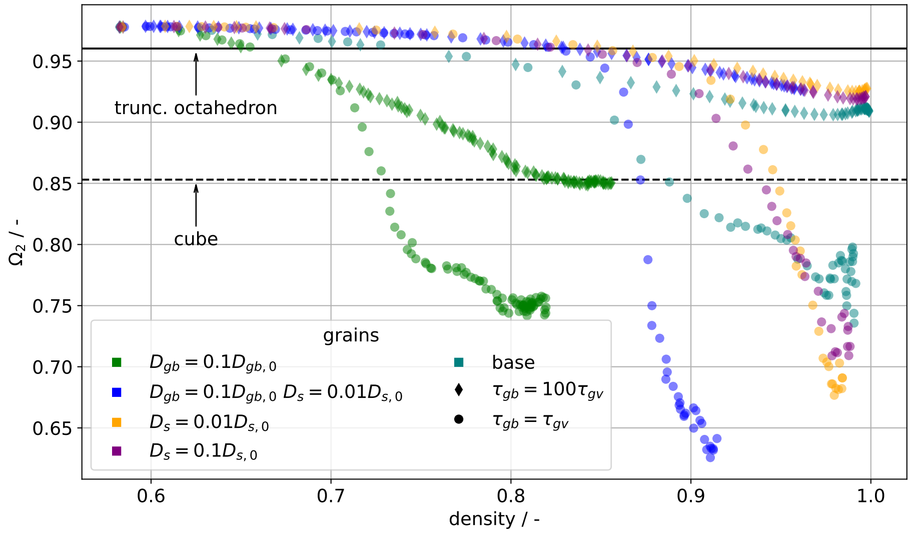

Figure 2: The moment invariant $\widetilde{\Omega_2}$ for the grain domain is depicted.

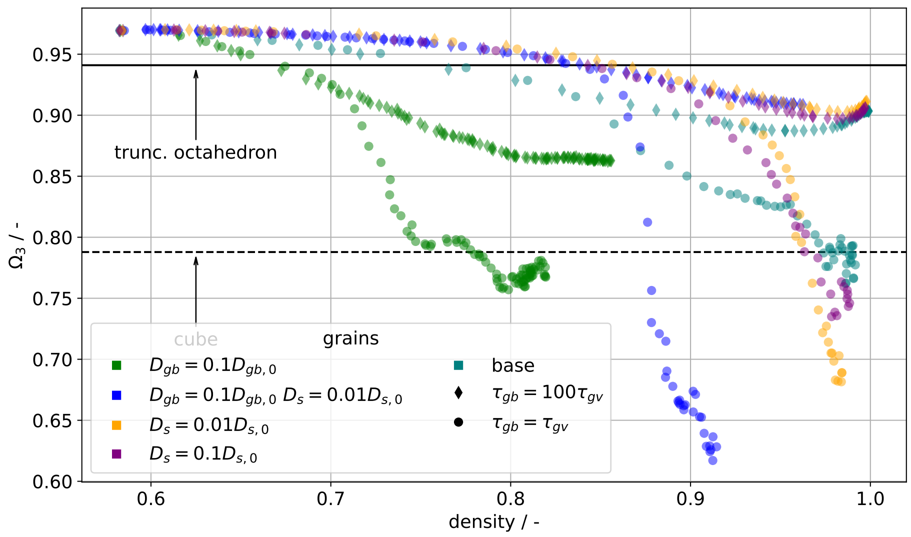

Figure 3: The moment invariant $\widetilde{\Omega_3}$ for the grain domain is depicted.

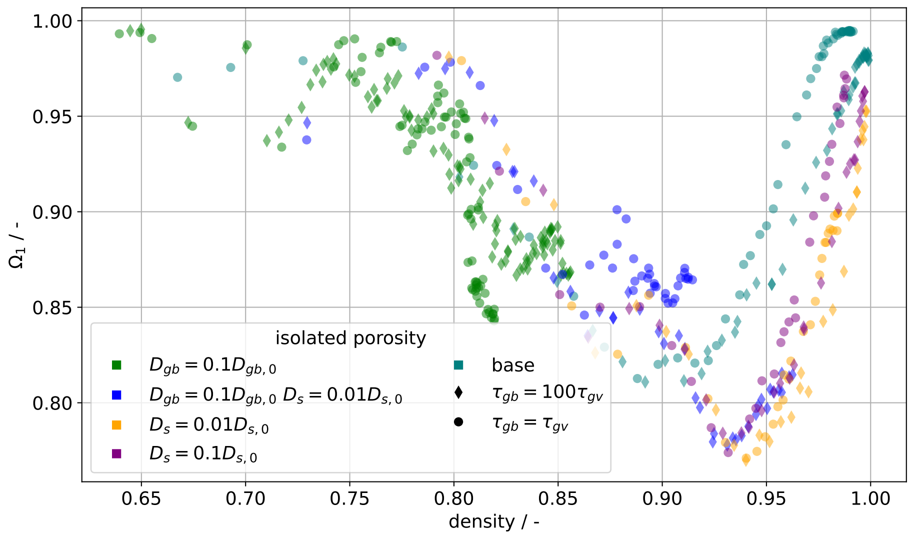

Figure 4: The moment invariant $\widetilde{\Omega_1}$ for the isolated porosity domain is depicted.

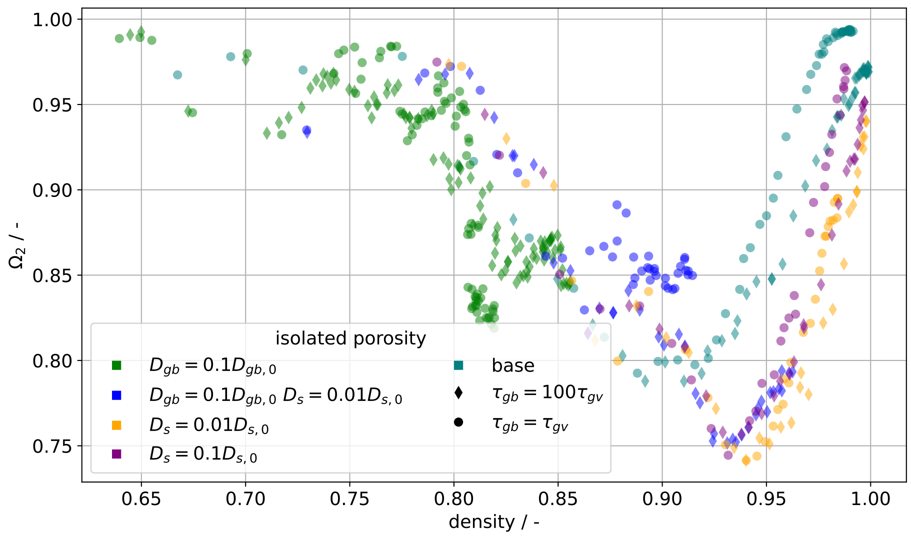

Figure 5: The moment invariant $\widetilde{\Omega_2}$ for the isolated porosity domain is depicted.

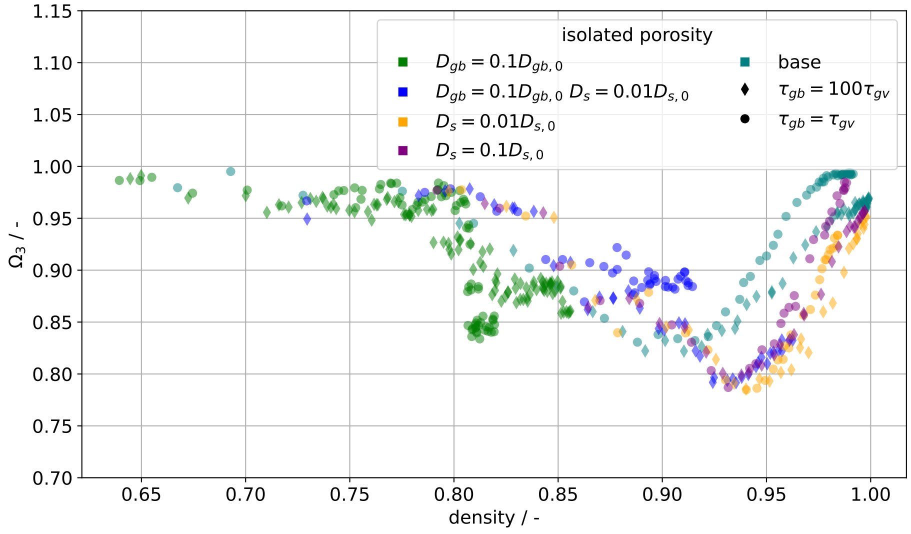

Figure 6: The moment invariant $\widetilde{\Omega_3}$ for the isolated porosity domain is depicted.

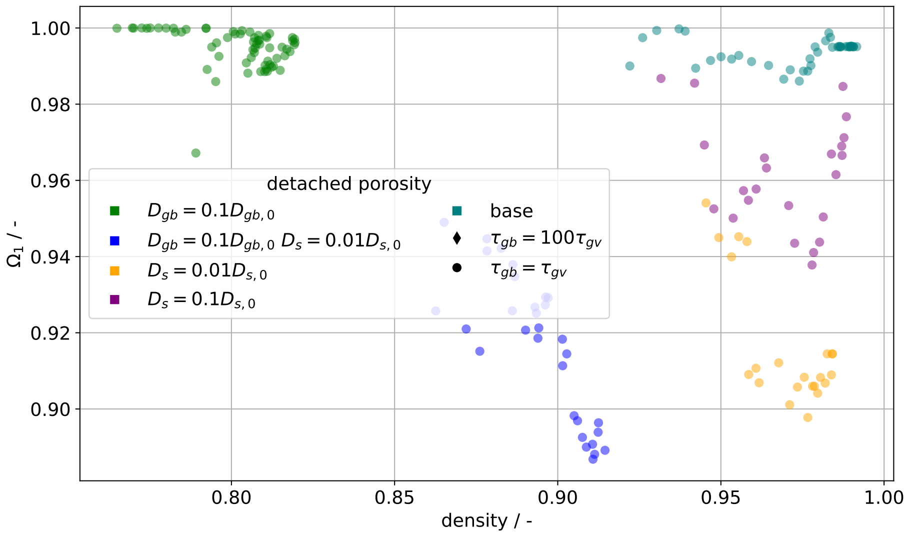

Figure 7: The moment invariant $\widetilde{\Omega_1}$ for the detached porosity domain is depicted.

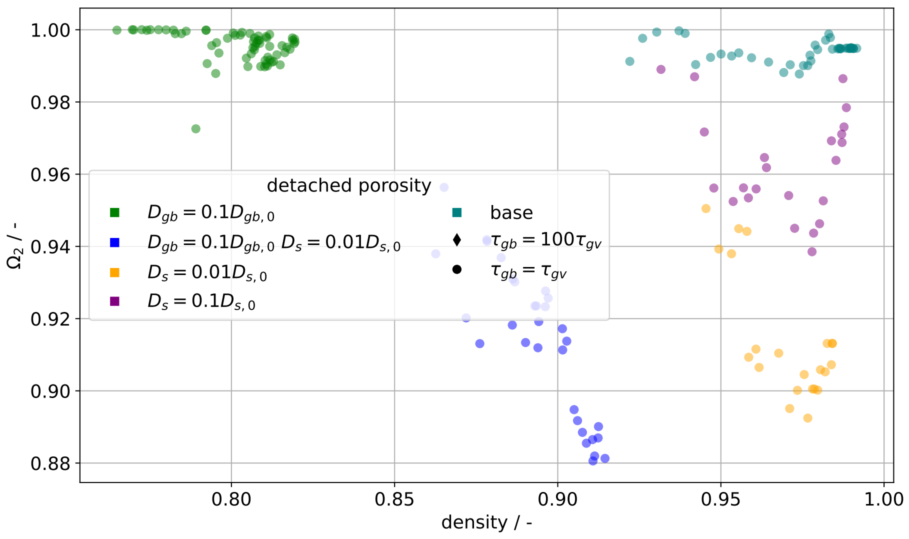

Figure 8: The moment invariant $\widetilde{\Omega_2}$ for the detached porosity domain is depicted.

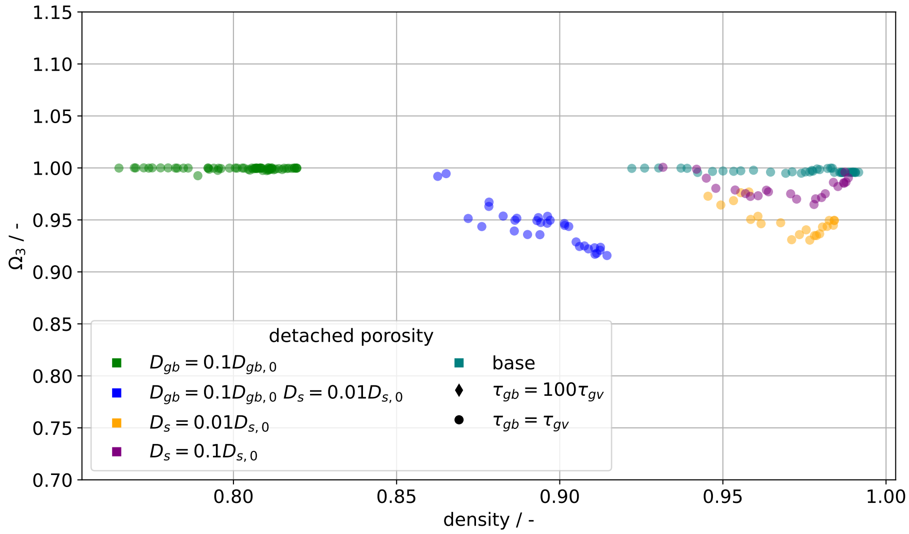

Figure 9: The moment invariant $\widetilde{\Omega_3}$ for the detached porosity domain is depicted.
