# Supplementary material for: Revealing process and material parameter effects on densification via phase-field studies
Source: Sci Rep. 2024 Mar 4;14:5350. doi: 10.1038/s41598-024-51915-w (PMC10912692; doi:10.1038/s41598-024-51915-w)
Supplement: Supplementary file 1 — Supplementary Information. [file 41598_2024_51915_MOESM1_ESM.zip › supmat/strain-analysis-creep.pdf]

Strain analysis of creep simulations conducted in the paper "Revealing process and material parameter effects on densification" by Seiz et al. (2023).

The strain calculation is based on particle-wise displacements from their original positions. Once all particle displacements are known, they are averaged over slabs placed in one direction, which yields an average displacement per slab. Since slab position and displacement are known, finite differences yield the strain per slab. This strain is assumed to be sufficiently homogeneous to be averaged over.

In [1]:
```
%matplotlib inline
# notebook backend doesn't work on jupyterlab and widgets seems to die
# exclusively on binder, but works fine locally
# hence if you want interactivity in the plots:
# restart kernel, change the inline of the above to notebook and
# go to the notebook view via changing the url from
# https://hub.ovh2.mybinder.org/whatever/lab?
# to
# https://hub.ovh2.mybinder.org/whatever/tree/
# NB that the domain can be different depending on whereever you land.
# the important part is replacing lab with tree to get the notebook view
import numpy as np
import pandas as pd
import sympy as sp
import matplotlib.pyplot as plt
import matplotlib as mpl
import glob
from pathlib import Path
import re
import natsort
import scipy.optimize
import scipy.interpolate
from helper import *
X, Y, Z = 0, 1, 2
dirs = np.array([X, Y, Z])
dirnames = ["X", "Y", "Z"]
fsize = (10,5)

#mpl.rcParams.update({'font.size': 13})
mpl.rcParams['figure.figsize'] = (10,5)
mpl.rcParams.update({'font.size': 18})
mpl.rcParams.update({"axes.grid" : True})

mpl.rcParams['axes.prop_cycle'] = mpl.cycler(color=coloursen)
```

In [2]:
```
def u2strain(ulist, poslist):
    epsen = [[0,0,0]] # initial condition are unstrained
    epsen_std = [[np.array((0,0,0)), np.array((0,0,0))]]
    for pos, u in zip(poslist, ulist):
        divu = []
        for tdir in [X, Y, Z]:
            divu.append(np.array(np.gradient(u[tdir], pos[tdir] )))
        divu = np.array(divu)
        avgeps = np.average(divu, axis=1)
        epsen.append(avgeps)

        mineps, maxeps = avgeps-np.min(divu, axis=1), np.max(divu, axis=1)-avgeps # f
        epsen_std.append([mineps, maxeps])

    epsen = np.array(epsen)
    epsen_std = np.array(epsen_std)
```

In [3]:
```
def calcStrain(coms):
    first = coms[0]
```

```python
    ulist = []
    poslist = []
    #1d slices
    for frame in range(1,coms.shape[0]):
        nonzero = coms
        curcom = coms[frame]
        nonzerofilt = np.any(curcom != 0, axis=1)
        comsF = first[nonzerofilt]
        disp = (comsF-coms[frame][nonzerofilt])

        minen, maxen = [], []
        for testdir in [0,1,2]:
            minen.append(np.min(comsF[:,testdir]))
            maxen.append(np.max(comsF[:,testdir]))
        bincounts = 6 #[12, 12, 12]
        # -+ so we cut off the edges, roughly

        binedges = []
        binpos = []
        for testdir in [0,1,2]:
            binedges.append(np.linspace(minen[testdir]+12, maxen[testdir]-12, bincoun
            binpos.append(np.zeros(bincounts-1))
        #np.linspace(xmin+12, xmax-12, bincount)
        #bins = []
        ubinned = np.zeros((3, bincounts-1))

        for testdir in [X,Y,Z]:
            filtcount = 0
            for dircount in range(bincounts-1):
                mins = binedges[testdir][dircount]
                maxs = binedges[testdir][dircount+1]
                filt = (comsF[:,testdir] > mins) & (comsF[:,testdir] < maxs)
                binpos[testdir][dircount] = 0.5*(mins + maxs)

                if np.any(filt):
                    umeans = np.average(disp[filt,testdir])
                    ubinned[testdir,dircount] = umeans

                else:
                    ubinned[testdir,dircount] = np.nan
                #if testdir == X:
                #    filtcount += np.count_nonzero(filt)
            #if testdir == X:
            #    tmpr = rs[frame]
            #    rfilt = tmpr > 0
            #    avgr = np.average(tmpr[rfilt], weights = vols[frame][rfilt])
            #    #print(filtcount, times[frame], avgr)

        ulist.append(ubinned)
        poslist.append(binpos)
    epsen = [[0,0,0]] # initial condition are unstrained
    epsen_std = [[np.array((0,0,0)), np.array((0,0,0))]]
    for pos, u in zip(poslist, ulist):
        divu = []
        for tdir in [X, Y, Z]:
            divu.append(np.array(np.gradient(u[tdir], pos[tdir] )))
        divu = np.array(divu)
        avgeps = np.average(divu, axis=1)
        epsen.append(avgeps)

        mineps, maxeps = avgeps-np.min(divu, axis=1), np.max(divu, axis=1)-avgeps # f
        epsen_std.append([mineps, maxeps])

    epsen = np.array(epsen)
    epsen_std = np.array(epsen_std)
    return epsen, epsen_std, ulist, poslist
```

In [4]:
```python
bpath = "data/"
comnames = glob.glob(bpath + "*creep*_comss.npy")
names = []
datdict =  {}
uniq_ps = set()
for comname in comnames:
    p = Path(comname)
    bn = p.name
    names.append(bn)
    coms = np.load(comname)
    densdf = pd.read_csv(comname.replace("_comss.npy", "_density.dat"), sep=" ")
    datdict[bn] = {"coms": coms, "dens": densdf}
    epsen, epsen_std, _, _ = calcStrain(coms)
    datdict[bn]["strain"] = epsen
    datdict[bn]["strain_std"] = epsen_std
    p = float(muHandler(bn, pprint=None))
    datdict[bn]["meta"] = { "p": p}
    uniq_ps.add(p)
```

After an initial transient, the strain is observed to be a linear function of time, characteristic of second stage creep. The porosity decreases at first since overall enough GBs with a sufficiently large compressive stress exist. The porosity increases eventually due to these GBs reducing their stresses sufficiently such that the tensile stresses become dominant.

In [5]:
```python
fig, ax = plt.subplots(figsize=(fsize[0]+2,fsize[1]))
dirstyle = {X: "-o", Y: "-x", Z: "-v"}
coloursen = ["green", "orange", "blue", "purple"]
ps = sorted(list(uniq_ps))
pcolours = {k:v for k,v in zip(ps, coloursen)}
for k, v in datdict.items():
    #if "mu-3" in k:
    #    continue
    eps = v["strain"]
    eps_std = v["strain_std"]
    densdict = v["dens"]
    densi = densdict["density"].values
    time = densdict["time"].values
    time -= time[0] # normalize since continued from other simulation with different
    time2 = time * t0  * 1e3 # ms
    # the simulations were actually run to different times
    # for ease of presentation in the paper they are cut to a fixed time
    # the behavior doesn't change if you comment out the if/else block and just set f
    if "mu-2" not in k:
        filt = time2 < 0.11
    else:
        filt = time2 < np.inf
    stress = v["meta"]["p"]
    color = None
    for tdir in dirs:
        if "nuc" in k:
            color = coloursen[-2]
        else:
            color=pcolours[stress]
        ax.plot(time2[filt], eps[:,tdir][filt], dirstyle[tdir]
                    , color=color, alpha=0.5)
    ax.plot(time2[filt], 1-densi[filt], "--", color=color, alpha=0.5)
#, label="p = %.3g MPa" % (stress)
for p in ps[::-1]:
    ax.plot([], [], color = pcolours[p], label = r"$\sigma$ = %.3g MPa" % (mu2MPa(p))
#ax.plot([], [], color = coloursen[-2], label=r"$\sigma$ = %.3g MPa, w/ nuc." % (mu2M

for tdir in dirs:
    ax.plot([], [], dirstyle[tdir].replace("-", ""), color="black", label=dirnames[td
```

```
ax.plot([], [], "--", color="black", label="porosity")
ax.grid(True)
ax.set_xlabel("time / ms")
ax.set_ylabel("strain or porosity / -")

ax.legend(ncol=2, fontsize="small")
fig.tight_layout()
#fig.savefig("creep-strain.pdf", bbox_inches="tight")
```

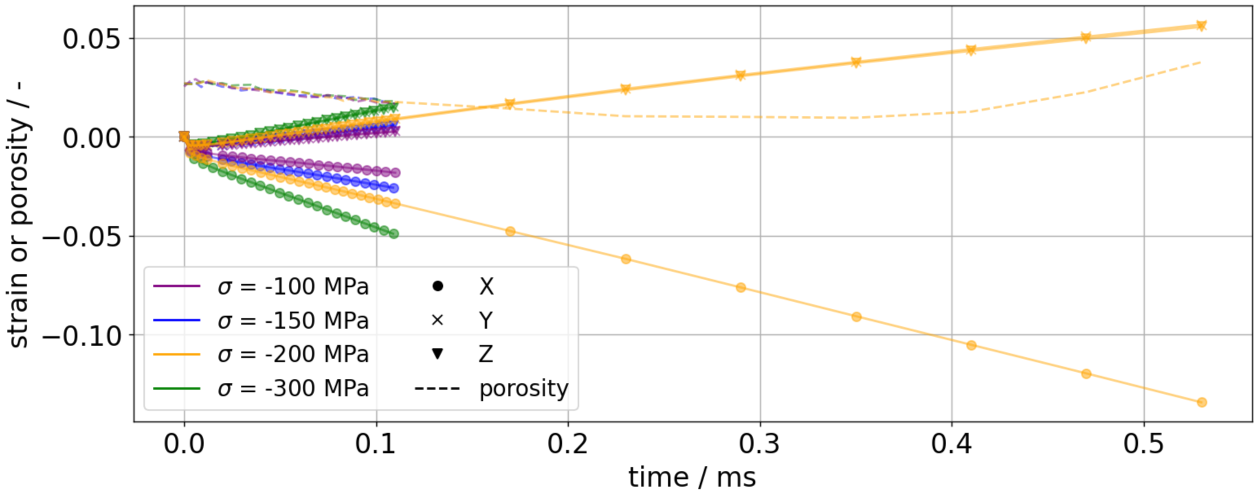

There is a slight slope to the strain rates. This is most likely the influence of changing capillary pressure as the surfaces adjust.

In [6]:

```
fig, ax = plt.subplots()
dirstyle = {X: "-o", Y: "-x", Z: "-v"}
coloursen = ["green", "orange", "blue", "purple"]
#ps = [-2.0, -1.0]
#pcolours = {k:v for k,v in zip([-2.0, -1.0], coloursen)}
for k, v in datdict.items():
    eps = v["strain"]
    eps_std = v["strain_std"]
    densdict = v["dens"]
    densi = densdict["density"].values
    time = densdict["time"].values
    time -= time[0] # normalize


    stress = v["meta"]["p"]
    color = None
    for tdir in dirs:
        if "nuc" in k:
            color = coloursen[-2]
        else:
            color=pcolours[stress]
        depsdt = np.gradient(eps[:,tdir], time)
        ax.plot(time*t0*1e3, depsdt,
                    dirstyle[tdir], color=color, alpha=0.5)
#, label="p = %.3g MPa" % (stress)
for p in ps[::-1]:
    ax.plot([], [], color = pcolours[p], label = r"$\sigma$ = %.3g MPa" % (mu2MPa(p)))
#ax.plot([], [], color = coloursen[-2], label=r"$\sigma$ = %.3g MPa, w/ nuc." % (mu2M

for tdir in dirs:
    ax.plot([], [], dirstyle[tdir].replace("-", ""), color="black", label=dirnames[td
ax.grid(True)
ax.set_xlabel("simulation time / -")
ax.set_ylabel("strain rate / -")
```

```
ax.legend(ncol=2)
fig.tight_layout()
```

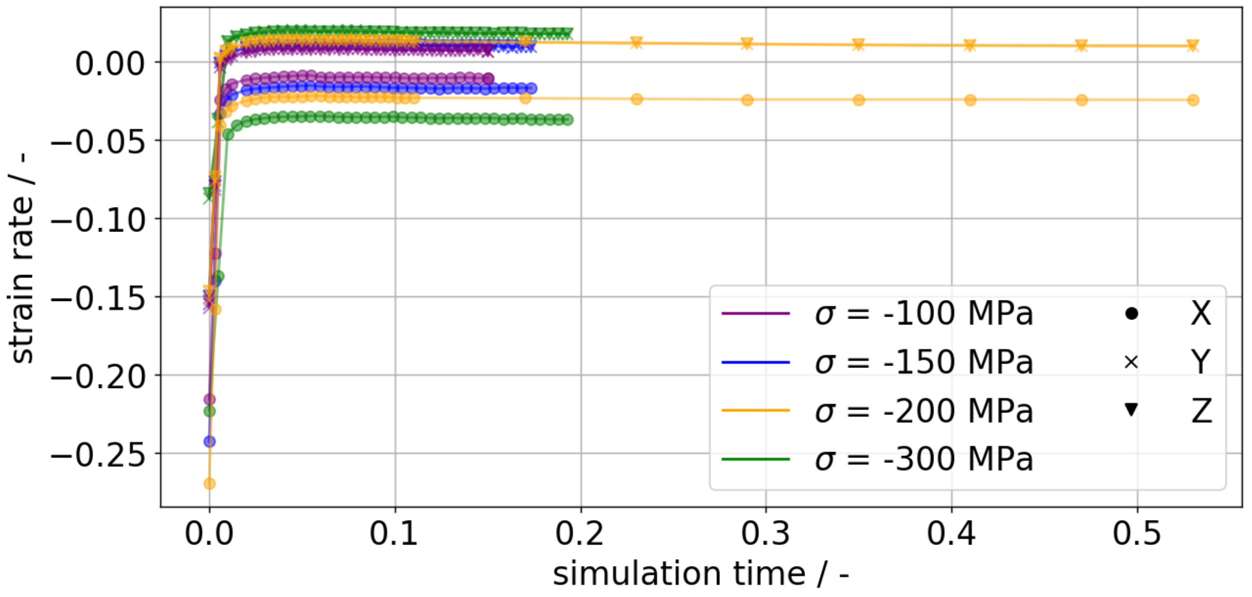

Similar to the pressure dependence, the (somewhat) steady strain rates can be gathered per stress and fitted against a linear function to test whether the dependence in stress is linear. The resulting good visual fit and high R^2 suggests that the strain rate is indeed a linear function of the applied stress.

In [7]:
```
def linfunc(x, A, n):
    return n*x + A
#stressen.shape, depsdts[:,0]
```

In [8]:
```
fig, ax = plt.subplots()
dirstyle = {X: "-o", Y: "-x", Z: "-v"}
coloursen = ["green", "orange", "blue", "purple"]
#ps = [-2.0, -1.0]
#pcolours = {k:v for k,v in zip([-2.0, -1.0], coloursen)}
depsdts = []
stressen = []
for k, v in datdict.items():
    eps = v["strain"]
    eps_std = v["strain_std"]
    densdict = v["dens"]
    densi = densdict["density"].values
    time = densdict["time"].values
    time -= time[0] # normalize


    idx = np.argmax(time*t0*1e3 > 0.1)
    stress = v["meta"]["p"]
    stressen.append(stress)
    depsdts.append([])
    color = None
    for tdir in dirs:

        if "nuc" in k:
            color = coloursen[-2]
        else:
            color=pcolours[stress]
        depsdt = np.average(np.gradient(eps[:,tdir], time)[idx-2:idx+2])
        #depsdt = np.average(np.gradient(eps[:,tdir], time)[:-10])
        depsdts[-1].append(depsdt)

stressen = np.array(stressen)
```

```python
depsdts = np.array(depsdts)

xrates = depsdts[:,0]
sortidx = stressen.argsort()
stressen = stressen[sortidx[::-1]]
xrates = xrates[sortidx[::-1]]
ax.plot(stressen, xrates, "o", label="data")

popt, pcov = scipy.optimize.curve_fit(linfunc, stressen, xrates,
                         full_output=False)
y = xrates
y_fit = linfunc(stressen, *popt)
# residual sum of squares
ss_res = np.sum((y - y_fit) ** 2)

# total sum of squares
ss_tot = np.sum((y - np.mean(y)) ** 2)

# r-squared
r2 = 1 - (ss_res / ss_tot)

ax.plot(stressen, linfunc(stressen, *popt), label="fit, $R^2=%.6g$" % (r2))
#
#for tdir in dirs:
#    ax.plot([], [], dirstyle[tdir].replace("-", ""), color="black", label=dirnames[t
ax.grid(True)

ax.set_xlabel("stress / -")
ax.set_ylabel("strain rate / -")
ax.legend()
fig.tight_layout()
```

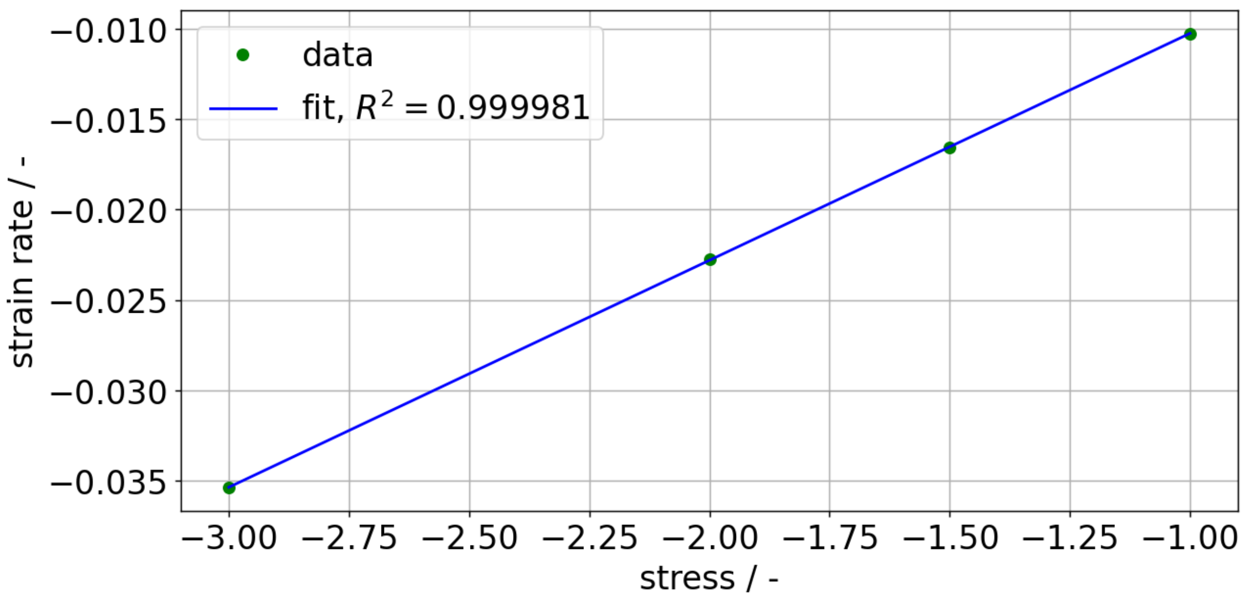

In [ ]:
