# Supplementary material for: Revealing process and material parameter effects on densification via phase-field studies
Source: Sci Rep. 2024 Mar 4;14:5350. doi: 10.1038/s41598-024-51915-w (PMC10912692; doi:10.1038/s41598-024-51915-w)
Supplement: Supplementary file 1 — Supplementary Information. [file 41598_2024_51915_MOESM1_ESM.zip › supmat/analysis.pdf]

Main analysis of the sintering simulations conducted in "Revealing process and material parameter effects on densification" by Seiz et al. (2023).

This contains most of the plots shown in the paper as well as some additional analysis.

In [1]:
```
%matplotlib notebook
# notebook backend doesn't work on jupyterlab and widgets seems to die
# exclusively on binder, but works fine locally
# hence if you want interactivity in the plots:
# restart kernel, change the inline of the above to notebook and
# go to the notebook view via changing the url from
# https://hub.ovh2.mybinder.org/whatever/lab?
# to
# https://hub.ovh2.mybinder.org/whatever/tree/
# NB that the domain can be different depending on whereever you land.
# the important part is replacing lab with tree to get the notebook view
import matplotlib as mpl
import matplotlib.pyplot as plt
import os
import sys
import matplotlib.patches as patches
import glob
import pandas as pd
from scipy      import optimize, interpolate, stats
# which function is used for fitting is somewhat inconsistent
import numpy as np
import natsort
import warnings
from pathlib import Path
from helper import *
import re
import matplotlib.patches as patches
from matplotlib.offsetbox import OffsetImage, AnnotationBbox
#mpl.rcParams.update({'font.size': 16})
mpl.rcParams.update({'font.size': 14}) # 18 is in creep
fsize = (10,5)
mpl.rcParams['figure.figsize'] = (10,6)
mpl.rcParams.update({"axes.grid" : True})

# volss: [frame, grain volumes], neighss: [frame, neighbours per grain], com
# all in nondim. cell units
bnen = ["volss", "neighss", "comss"]
npydats = {k: "_"+k+".npy" for k in bnen}

# moment data is also in npy format, but requires a bit of special handling

natkey = natsort.natsort_keygen()


tfacs = [1, 10, 100]
tfacs_ls = ["-o", "-s", "-d"]
lsen = {float(k): v for k,v in zip(tfacs, tfacs_ls)}

densi_end = "_density.dat"
```

```
isolated_end = "_isolated2.dat"
ns_end = "_revns2.dat"
energy_end = "_energy.dat"

#this could be eval'd
dfnames = {"ns": ns_end,
           "isolated": isolated_end,
           "energy": energy_end
          }

domaintypes = ["g", "i", "d"] # for invariant calc: grains, isolated pores,
domainnames = {"g": "grains", "i": "isolated porosity", "d": "detached poros


def mkName_noGG_nomu(s):
    handlers = [packHandler, rHandler]
    return mkName(s, handlers=handlers)
def mkName_noGG(s):
    handlers = [packHandler, rHandler, muHandler]
    return mkName(s, handlers=handlers)
def mkName_GG(s, handlers=None):
    # allow some flexibility here since we might want to look at only subspa
    #packHandler, rHandler not since only one box size + particle size is co
    if handlers is None:
        handlers = [taugkfHandler, DgbfHandler, DsfHandler, relaxfHandler]
    return mkName(s, handlers=handlers)
```

In [2]:
```
mpl.rcParams['axes.prop_cycle'] = mpl.cycler(color=coloursen)
```

All stored data is in nondimensional units, with the nondimensionalization scales given in helpers.py.

In [3]:
```
def readDat(densitypath, datdict, names, readNpy=True):
    p = Path(densitypath)
    name = p.name.replace(densi_end, "")
    names.append(name)

    idict = {}

    df = pd.read_csv(densitypath, sep=" ")
    # density is with MC+convex hull
    # gsum/isum are direct sums of (1-phiv) (entire domain) and phi_i (only
    # thus gsum / (gsum+isum) gives an accurate estimate of isolated porosit
    # potentially after gsum/(gsum+isum) went through a global minimum only
    # and hence could be used for an accurate estimate of total porosity at
    # however, this will need to be investigated separately
    time = df["time"]
    dens = df["density"]
    #diffts = np.diff(time, prepend=time[0])
    drhodt = np.gradient(dens, time)
    # NB: there are some with very low diffts, don't trust them -> turn to r
    df.loc[:,'densirate'] = pd.Series(drhodt, index=df.index)
    #df["densirate"] = drhodt
    #print(df["densirate"].shape)
```

```
    idict["density"] = df.copy()

    for kname, end in dfnames.items():
        try:
            isodatpath = densitypath.replace(densi_end, end)
            df = pd.read_csv(isodatpath, sep=" ")
            idict[kname] = df #.copy()

        except Exception as e:
            #print(e)
            #print("no %s data for %s" % (kname, name))
            pass # don't care
    try:
        inv_datdict = {}
        for domaintype in domaintypes:
            inv_datdict[domaintype] = {}
            for datatype in ["vol", "mom"]: # "com"
                f = str(p.parent)+"/" + p.name.replace(densi_end, "_%s%s.npy
                #for f in glob.glob(simdir+"/*_%s%s*npy" % (domaintype, data
                inv_datdict[domaintype][datatype] = np.load(f, allow_pickle=
        idict["invariants"] = inv_datdict
    except Exception as e:
        print(e)
        print("no moment data for", name)
        pass
    # no eval on these immediately, just read in
    dxval = float(redx.search(name).groups()[0])
    if readNpy:
        for k, v in npydats.items():
            #npath = str(p.parent)+"/" + p.name.replace("density.dat", v)
            npath = str(p.parent)+"/" + p.name.replace(densi_end, v)
            dat = np.load(npath)
            idict[k] = dat.copy()

        # avgs for data which is often used
        gv = idict["volss"]
        frames, _ = gv.shape
        rs = np.zeros(frames)
        for f in range(frames):
            tmp = gv[f]
            rv = vol2r(tmp)
            # once r < epsilon, pretty much only interface remains and hence
            avg = physSizeNm ( np.average(rv[rv > 4])*dxval )
            #avg = physSizeNm(vol2r((np.average(tmp[tmp != 0])))*dxval) # vo
            rs[f] = avg
        idict["avgRadius"] = rs #.copy()

        # NB: the filters for avg(N_c) and avg(r) are different currently

        gn = idict["neighss"]
        frames, _ = gn.shape
        ns = np.zeros(frames)
        for f in range(frames):
            tmp = gn[f]
            avg = np.average(tmp[tmp != 0]) # can have zero neighs because i
            ns[f] = avg
```

```
        idict["avgNeighs"] = ns.copy()


    idict["meta"] = {}
    idict["meta_num"] = {}
    # nondimensionalized base values
    tauf0 = 0.08
    Dsf0 = 169
    Dgbf0 = 55
    for identifier, handler in zip(["tauf", "Dsf", "Dgbf"],
                                    [taufHandler, DsfHandler, DgbfHandler]):
        idict["meta_num"][identifier] = eval(identifier+"0")*float(handler(r

    idict["meta"]["dx"] = dxval #

    for identifier, handler in glob_handlers.items():
        val = handler(name) # so you don't have to know the formatting...
        idict["meta"][identifier] = val

    datdict[name] = idict.copy()
```

In [4]:

```
datdict = {}
names = []
# todo update
bpath = "data/"
pat = "pack400*_weno_*density.dat"
#pat = "pack200_dx0.1_r12_mu0_weno_md_musurfPositive_mpt_*density_combined.d
#pat = "pack400_dx0.1_r12*_GG_tauf100_relaxf1_Dgbf0.1*density.dat"
paths = natsort.natsorted(glob.glob(bpath+pat))
for p in paths:
    #"Dgbf0.5" in p or
    # there's a bit more data here than presented in the paper
    # Dgbf0.5 halves the GB diffusion, the effect is just as you would expec
    # creep is investigated in another notebook
    # the relaxf* are factors on the relaxation time t_r, which showed the e
    # convergence behaviour, i.e. once t_r is sufficiently small (relaxf<N>
    # the densification curve converges
    if "Dgbf0.5" in p or  "creep" in p or "relaxf10" in p or "relaxf200" in
        continue


    # if you only want to look at density/isolated pores stuff, don't read i
    # that's mainly per-grain information which isn't relevant for those but
    readDat(p, datdict, names, readNpy=True)
names_noGG = []
names_GG = []
for name in names:
    if "GG" not in name:
        names_noGG.append(name)
    else:
        names_GG.append(name)
names_GG = natsort.natsorted(names_GG)
names_noGG = natsort.natsorted(names_noGG)
```

Pressure influence is considered first, with creep evaluation being in a separate notebook

since that requires a bit more computation before plotting.

In [5]:
```
fig, ax = plt.subplots()
trpacks = set()
trsizen = set()

ks = []
for k, v in datdict.items():
    if "GG" not in k and "dx0.1" in k and "r12" in k and "pack400" in k:
        ks.append(k)

        coms = v["comss"]

        epsen, epsen_std, _, _ = calcStrain(coms)
        v["strain"] = epsen
        v["strain_std"] = epsen_std

ks = natsort.natsorted(ks, alg=natsort.REAL)
ps = np.array([0, 0.5, 1, 2])
ps = ps * Er0 / 1e6
ds, ts = [], []
epss = [] # prep for continuum model
for k in ks:
    v = datdict[k]
    #and "r12" in k
    # and "mu0" in k

    if "GG" not in k and "dx0.1" in k and "r12" in k and "pack400" in k:
        print(k)
        pack = v["meta"]["pack"]
        R = v["meta"]["R"]
        trsizen.add(R)
        trpacks.add(pack)
        col = coloursR[R]
        ls = ls_pack[pack]

        densdf = v["density"]
        time = densdf["time"]
        dens = densdf["density"]
        stddev = densdf["sddensity"]
        filt = (dens < 1) #& (time <= 3) #& (time*t0*1e3 < 1.2*t0*1e3)
        xdat = time[filt][:-1]
        ydat = dens[filt][:-1]
        err = stddev[filt]
        lab = mkName(k, handlers=[muHandler], nodefaultprint=False)
        ds.append(ydat) # list of densities
        epss.append(np.sum(v["strain"][filt,:], axis=1)[:-1])
        ts.append(xdat) # list of times
        mevery=1
        ax.plot(xdat*t0 * 1e3, ydat,
               markevery=mevery,
               label=lab)

#title="$400^3$ R=12 nm",
ax.legend( ncol=2)
```

```
ax.set_xlabel("time / ms")
ax.set_ylabel("density / -")
fig.tight_layout()
##fig.savefig("densi-md-press.pdf", bbox_inches="tight")
```

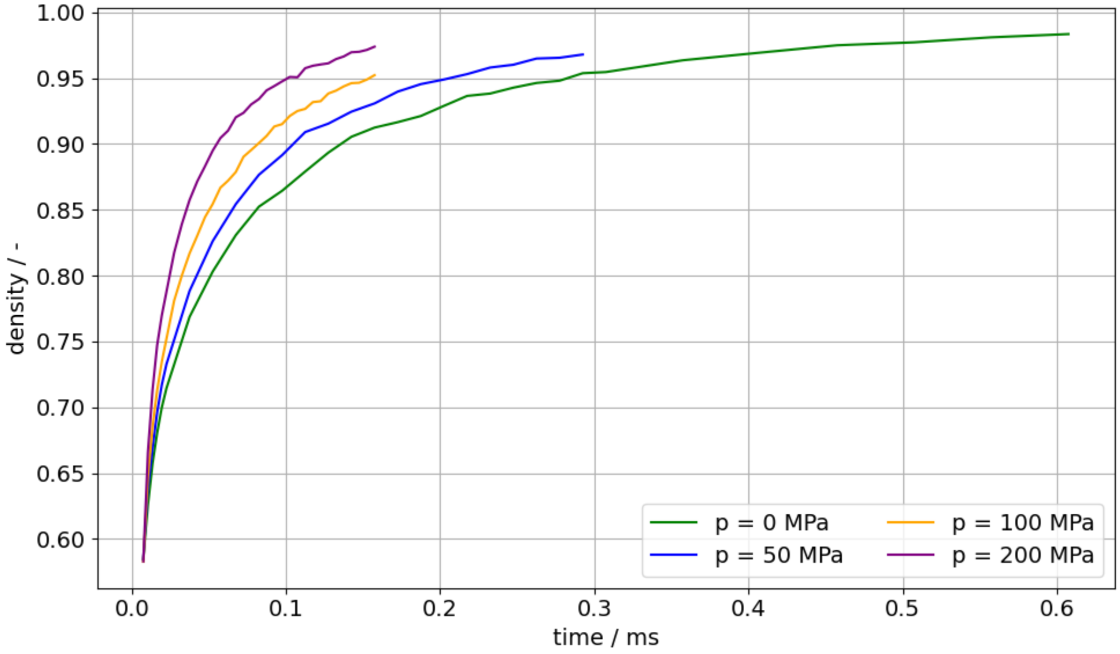


```
pack400_dx0.1_r12_mu0_weno_md_musurfPositive_mpt
pack400_dx0.1_r12_mu0.5_weno_md_musurfPositive_mpt
pack400_dx0.1_r12_mu1_weno_md_musurfPositive_mpt
pack400_dx0.1_r12_mu2_weno_md_musurfPositive_mpt
```

The influence of densification rate can be quantified by determining the densification rate at fixed density and plotting it over the applied pressure. Since generally the resulting densities do not need to match, this is done with an interpolation to a set of target densities.

In [6]:
```
def calcR2(func, xdata, ydata, popt):
    residuals = ydata- func(xdata, *popt)
    ss_res = np.sum(residuals**2)
    ss_tot = np.sum((ydata-np.mean(ydata))**2)
    r_squared = 1 - (ss_res / ss_tot)
    return r_squared
```

In [7]:
```
def linfunc(x, m, b):
    return m*x +b
def powfunc(x, m, b, a):
    return b + a*x**m
#popt, pcov = optimize.curve_fit(linfunc, xx, yy, p0=(1,1))
```

In [8]:
```
fig, ax = plt.subplots()

#rhos = np.linspace(0.75, 0.93, 10)
#p0 = x * Er0 / 1e6
```

```
testrhos = [0.8, 0.85, 0.9]
# you can change these as you like, but the result won't change for densitie
# since there are very few pores remaining for >0.95 density, the simulatior
for testrho in testrhos:
    drates = []
    for i, d in enumerate(ds):
        #trho = interpolate.interp1d(ds[i], ts[i])
        #drate = np.gradient(rhos, trho(rhos))
        densirate = np.gradient(ds[i], ts[i]) # nondim -> s, therefore grad
        drho_ip = interpolate.interp1d(ds[i], densirate)
        drate = drho_ip(testrho)
        drates.append(drate)
        #drates.append(drate)
    drates = np.array(drates)

    drates =  drates/t0
    popt, pcov = optimize.curve_fit(linfunc, ps, drates, p0=(1,1))
    #popt, pcov = optimize.curve_fit(powfunc, ps, drates, p0=(1,1,1))
    R2 = calcR2(linfunc, ps, drates, popt)
    #avgrates = np.average(drates, axis=1)
    #ax.plot(ps, avgrates/avgrates[0], "-o")
    pax = ax.plot(ps , drates, "o", label=r"$\rho$ = %.2g, $R^2 = %.3g$" % (
    # should be in 1/s -> 1/ms
    col = pax[0].get_color()
    ax.plot(ps, linfunc(ps, *popt), "--", color=col)

    #ax.plot(ps, powfunc(ps, *popt), "--", color=col)
    #print(*popt)
ax.set_xlabel("pressure / MPa ")
ax.set_ylabel("densification rate / 1/s")
ax.legend()
#ax.plot(ds[i], densirate, "-o", label=ps[i])
#ax.legend()
fig.tight_layout()
##fig.savefig("densirate-pressure.pdf", bbox_inches="tight")
```

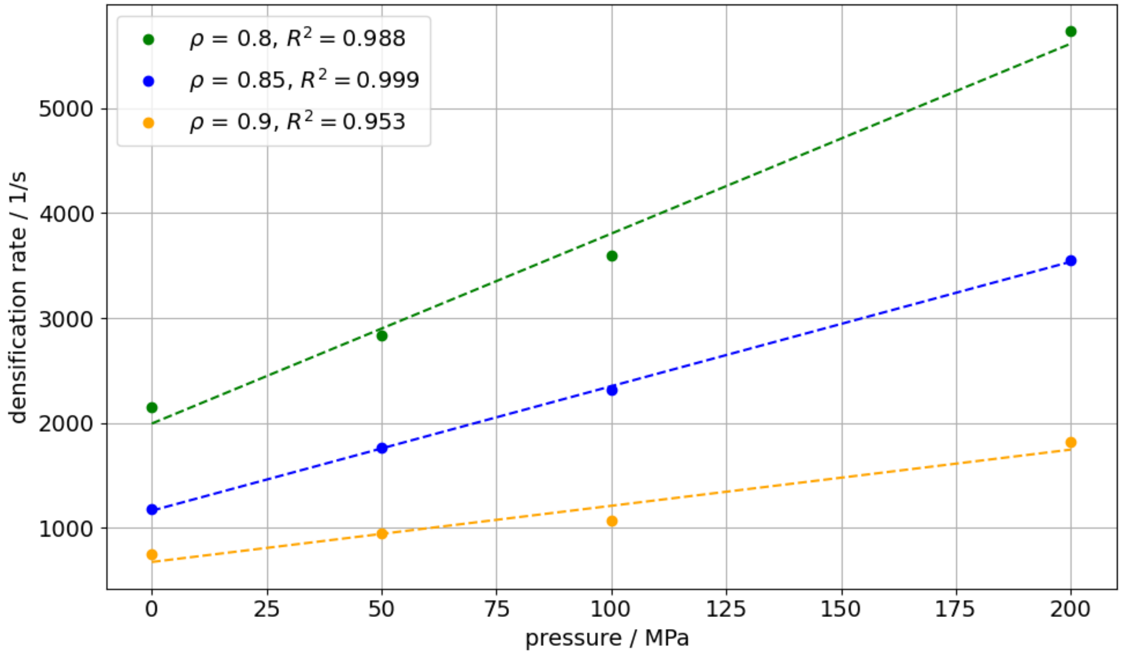

Before moving on from the pressure-assisted sintering we will make a connection to a continuum model of sintering.

In the following we will calculate the effective bulk viscosity K and sintering stress Σ by extrapolating back to the zero creep state. We assume in the spirit of Bordia & Scherer (https://doi.org/10.1016/0001-6160(88)90189-7):

$$\dot{\epsilon} = \frac{\sigma}{K} \tag{1}$$

i.e. the total strain rate $\dot{\epsilon}$ is given by the total hydrostatic stress $\sigma = \Sigma + p$ divided by the effective bulk viscosity K; the green body is assumed to be a viscous fluid, with K diverging to infinity as $\rho \to 1$ to be physical. We also assume that $\dot{\epsilon}$ is given at the same time as an affine, linear function of pressure

$$\dot{\epsilon} = mp + b \tag{2}$$

$$= \frac{\Sigma + p}{K} \tag{3}$$

and hence by comparing coefficients we have

$$K = \frac{1}{m} \tag{4}$$

$$\Sigma = bK = \frac{b}{m} \tag{5}$$

with m, b resulting from fitting the affine, linear function to our pressure-assisted sintering data.

The strain needs to be calculated first and afterwards the strain rate is plotted for a limited density range. Based on sampling a finer density range via interpolation, K, and Σ are then

determined as functions of density.

In [9]:
```
#mpl.rcParams.update({'font.size': 18})
fig, ax = plt.subplots(figsize=(fsize[0]+2,fsize[1]))

#rhos = np.linspace(0.75, 0.93, 10)
#p0 = x * Er0 / 1e6

#np.linspace(0.75, 0.95, 10) #
testrhos = [0.8, 0.85, 0.9]
for testrho in testrhos:
    drates = []
    for i, d in enumerate(ds):
        #trho = interpolate.interp1d(ds[i], ts[i])
        #drate = np.gradient(rhos, trho(rhos))

        densirate = np.gradient(epss[i], ts[i]) # nondim -> s, therefore gra
        drho_ip = interpolate.interp1d(ds[i], densirate)
        drate = drho_ip(testrho)
        drates.append(drate)
        #drates.append(drate)
    drates = np.array(drates)

    drates =  drates/t0
    popt, pcov = optimize.curve_fit(linfunc, ps, drates, p0=(1,1))
    #popt, pcov = optimize.curve_fit(powfunc, ps, drates, p0=(1,1,1))
    R2 = calcR2(linfunc, ps, drates, popt)
    #avgrates = np.average(drates, axis=1)
    #ax.plot(ps, avgrates/avgrates[0], "-o")
    pax = ax.plot(ps , drates, "o", label=r"$\rho$ = %.2g, $R^2 = %.3g$" % (
    # should be in 1/s -> 1/ms
    col = pax[0].get_color()
    ps2 = np.linspace(-300, 300, 25) # plot over ps2 to see the zero-creep x
    ax.plot(ps, linfunc(ps, *popt), "--", color=col)

    drates =  drates/t0
    popt, pcov = optimize.curve_fit(linfunc, ps, drates, p0=(1,1))
    #popt, pcov = optimize.curve_fit(powfunc, ps, drates, p0=(1,1,1))
    R2 = calcR2(linfunc, ps, drates, popt)
    K = 1/popt[0]
    eps3 = popt[1]
    sigma = eps3*K
    print(testrho, K, sigma, eps3)
    #Ks.append(K)
    #sigmas.append(sigma)

    #ax.plot(ps, powfunc(ps, *popt), "--", color=col)
    #print(*popt)
ax.set_xlabel("pressure / MPa ")
ax.set_ylabel("strain rate / 1/s")
ax.legend()
#ax.plot(ds[i], densirate, "-o", label=ps[i])
#ax.legend()
fig.tight_layout()
##fig.savefig("strainrate-pressure.pdf", bbox_inches="tight")
```

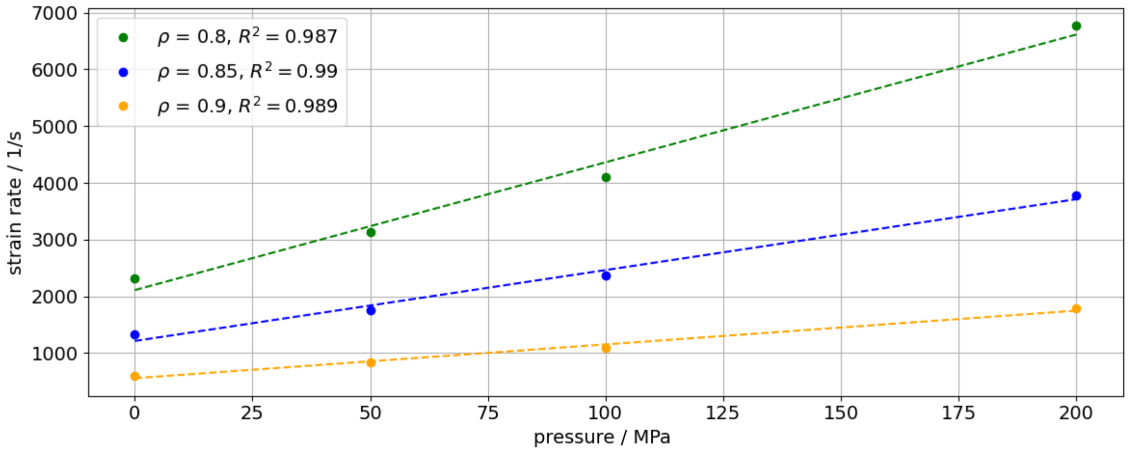


```
0.8 4.439118753111304e-06 93.65503144939899 21097662.99533162
0.85 8.002211119248637e-06 97.07854405596488 12131464.99252572
0.9 1.678992163202651e-05 93.44024552477725 5565257.990635375
```

In [10]:

```
testrhos = np.linspace(0.75, 0.95, 25)
#testrhos = [0.75, 0.8, 0.85, 0.9]
Ks = []
sigmas = []
for testrho in testrhos:
    drates = []
    pps = []
    for i, d in enumerate(ds):
        rhomax = np.max(ds[i])
        if (rhomax < 0.95):
            continue
        densirate = np.gradient(epss[i], ts[i]) # nondim -> s, therefore gra
        drho_ip = interpolate.interp1d(ds[i], densirate)
        drate = drho_ip(testrho)
        drates.append(drate)
        pps.append(ps[i])

    drates = np.array(drates)
    pps = np.array(pps)

    drates =  drates/t0
    popt, pcov = optimize.curve_fit(linfunc, pps, drates, p0=(1,1))
    #popt, pcov = optimize.curve_fit(powfunc, ps, drates, p0=(1,1,1))
    R2 = calcR2(linfunc, pps, drates, popt)
    K = 1/popt[0]
    eps3 = popt[1] # free strain rate, y-axis intercept
    sigma = eps3*K
    print("rho = ", testrho, "gives K = ", K, " and Sigma = ", sigma)
    Ks.append(K)
    sigmas.append(sigma)
```

```
rho =  0.75 gives K =  0.027483744757770816  and Sigma =  107.6976602110829
6
rho =  0.7583333333333333 gives K =  0.029977380134585674  and Sigma =  10
7.18065550081849
rho =  0.7666666666666666 gives K =  0.0329686697811742  and Sigma =  106.5
604721961527
rho =  0.775 gives K =  0.035685808595863705  and Sigma =  104.082212859808
42
rho =  0.7833333333333333 gives K =  0.03804142613822408  and Sigma =  99.0
6598124528934
rho =  0.7916666666666666 gives K =  0.040830304473884034  and Sigma =  95.
43002368932926
rho =  0.8 gives K =  0.044391187580514725  and Sigma =  93.65503161591919
rho =  0.8083333333333333 gives K =  0.049003674754355374  and Sigma =  95.
2166058872953
rho =  0.8166666666666667 gives K =  0.05508226165746026  and Sigma =  98.9
4338232018092
rho =  0.825 gives K =  0.060076921940002234  and Sigma =  97.4088434355011
rho =  0.8333333333333333 gives K =  0.06628393835347665  and Sigma =  98.0
4830650650928
rho =  0.8416666666666667 gives K =  0.07337435711631807  and Sigma =  99.4
8949335566714
rho =  0.85 gives K =  0.08002211119232576  and Sigma =  97.07854405598165
rho =  0.8583333333333333 gives K =  0.08708938358156311  and Sigma =  92.5
0667587084641
rho =  0.8666666666666667 gives K =  0.09568936942682615  and Sigma =  89.0
4687420415374
rho =  0.875 gives K =  0.10970416918855534  and Sigma =  87.95750906078659
rho =  0.8833333333333333 gives K =  0.1278167706985232  and Sigma =  91.14
444022891072
rho =  0.8916666666666666 gives K =  0.1441668168393531  and Sigma =  90.48
925801038688
rho =  0.8999999999999999 gives K =  0.16789921631995997  and Sigma =  93.4
4024552481103
rho =  0.9083333333333333 gives K =  0.20522354504501156  and Sigma =  101.
96353602090936
rho =  0.9166666666666666 gives K =  0.22299574223395682  and Sigma =  83.3
9980048652595
rho =  0.9249999999999999 gives K =  0.27942828905540745  and Sigma =  89.8
4231317308313
rho =  0.9333333333333333 gives K =  0.3473776855917298  and Sigma =  93.62
419223356626
rho =  0.9416666666666667 gives K =  0.3883959102188142  and Sigma =  83.83
51433581616
rho =  0.95 gives K =  0.5154804629711255  and Sigma =  83.31555515824616
```

In [11]:

```
fig, ax = plt.subplots(figsize=(10,4))
ax.plot(testrhos, Ks, label="$K$")
ax.set_xlabel("density / -")
ax2 = ax.twinx()
ax.set_ylabel("bulk viscosity $K$ / MPas")
pl = ax.plot([], [], label="$\Sigma$") # trick 23
ax2.plot(testrhos, sigmas, label="$\sigma$", color=pl[0].get_color())
ax2.set_ylabel("sintering stress $\Sigma$ / MPa")
ax.legend(loc="upper center")
ax2.grid(False)
```

```python
fig.tight_layout()
#fig.savefig("viscmodel.pdf", bbox_inches="tight")
```

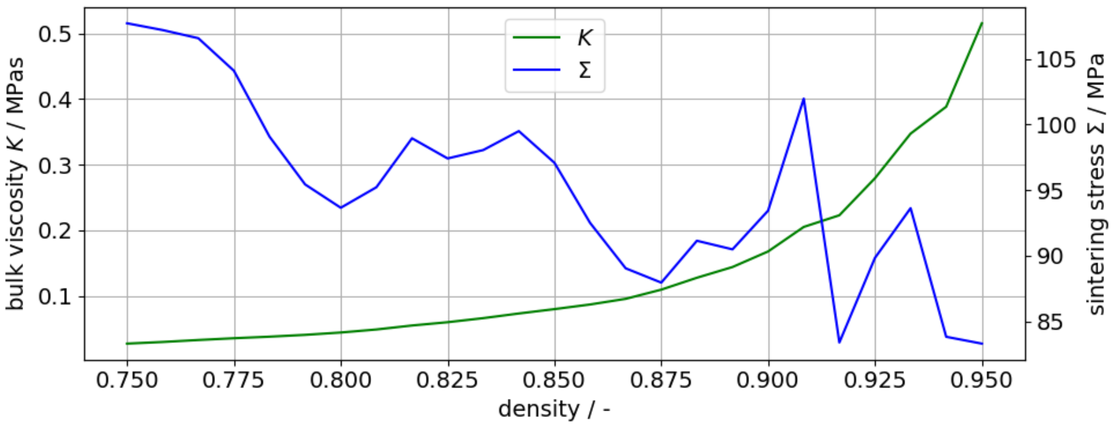

As one would expect, the bulk viscosity generally increases and seems to start diverging towards higher densities; the upper limit of density is restricted by the minimum density achieved across the simulations used for the fits, which is about 0.957. Of course, the simulations could have been continued to be closer to a density of 1, but at the time the pressure influence was the primary point of interest and thus higher densities were deemed unnecessary.

Note that the sintering stress oscillates quite wildly. This is likely due to undersampling of both the density space and pressure space by the simulations, resulting also in a larger error in the estimate of b in the fit (check pcov). Another possible influence is a breakdown of linearity in pressure close to the zero-creep stress, e.g. a switch from GB to surface diffusion control because the GB is no longer absorbing a sufficient number of vacancies to dominate the problem.

In [12]:
```python
basesim = "pack400_dx0.1_r12_GG_tauf100_weno_md_musurfPositive_mpt"
basesim1 = "pack400_dx0.1_r12_GG_tauf1_weno_md_musurfPositive_mpt"
basesims = [basesim, basesim1]
```

Next up are the simulations in which the mobilities (diffusional as a well as kinetic) of the interfaces were varied.

The density evolution over time is shown first. We can observe that the base case, as well as those with only reduced surface diffusion, effectively achieve full density. When grain boundary diffusion is reduced (green), though not simulated to infinite time, it is unlikely that full density would be achieved, even with kinetic suppression of grain growth. However, if surface diffusion is reduced sufficiently for this case it becomes quite likely that full density can be achieved again.

If grain growth is not kinetically suppressed, generally similar effects are observed. Within the present simulations two effects of grain growth on densification are evident: Before grain growth starts in "earnest", quantifiable by the maximum in grain boundary area shown later,

it tends to slightly increase densification rate. This is likely due to changes in pore coordination, though quantifying this for open porosity is somewhat challenging and hence will be left for future work. However, eventually this effect is superseded by more and more porosity detaching from grain boundaries and therefore reducing the achievable density.

In [13]:
```
fig, ax = plt.subplots()
#ax2 = ax.twinx()
trtaufac = {}


trsizen = set()
def iplot(fig, ax,  k):
    v = datdict[k]
    taufac = float(taufHandler(k, pprint=None, nodefaultprint=False))
    trtaufac[taufac] = taufHandler(k, nodefaultprint=False)
    ls = lsen[taufac]

    densdf = v["density"]
    gsize = v["avgRadius"]

    time = densdf["time"]
    dens = densdf["density"]
    filt = (dens < 1) #& (time <= 3) #& (time*t0*1e3 < 1.2*t0*1e3)
    xdat = time[filt]
    ydat = dens[filt]

    lab = mkName_GG(k)
    # and "_tauf100_" in k
    if len(lab.replace(" ", "")) == 0: # too many autohandlers in between ma
        lab = "base"
    col = gg_colors[lab]
    mevery=2
    # somehow or other make color of same Dgb/Ds var same
    pl = ax.plot(xdat*t0 * 1e3, ydat, ls,
        color=col, alpha=0.5,
           markevery=mevery,
                 #markersize=10
                )


ik, ilab = [], []
for k in names_GG:
    if "dx0.1" in k and "r12" in k and "pack400" in k and "tauf10_" not in k
    "relaxf" not in k or "relaxf1_" in k) and "_D" in k:
        lab = mkName_GG(k)
        ik.append(k)
        ilab.append(lab)

tmpilab = ilab.copy()
tmpilab.append("base")
ulab = sorted(set(tmpilab), key=natkey)
gg_colors = {k: v for k, v in zip(ulab, coloursen)}
gg_colors["base"] = coloursen[-1]
ik = [x for _, x in sorted(zip(ilab, ik), key=natkey)]
```

```
iplot(fig, ax,  basesim)
iplot(fig, ax,  basesim1)

for lab, col in gg_colors.items():
    ax.plot([], [], "s", color=col, label=lab)


for k in ik:
    iplot(fig, ax,  k)

for taufac, lab in trtaufac.items():
    ls = lsen[taufac]
    ax.plot([], [], ls.replace("-", ""), color="black", label=lab)


ax.set_xlabel("time / ms")
ax.set_ylabel("density / -")

ax.legend(ncol=2)
fig.tight_layout()
```

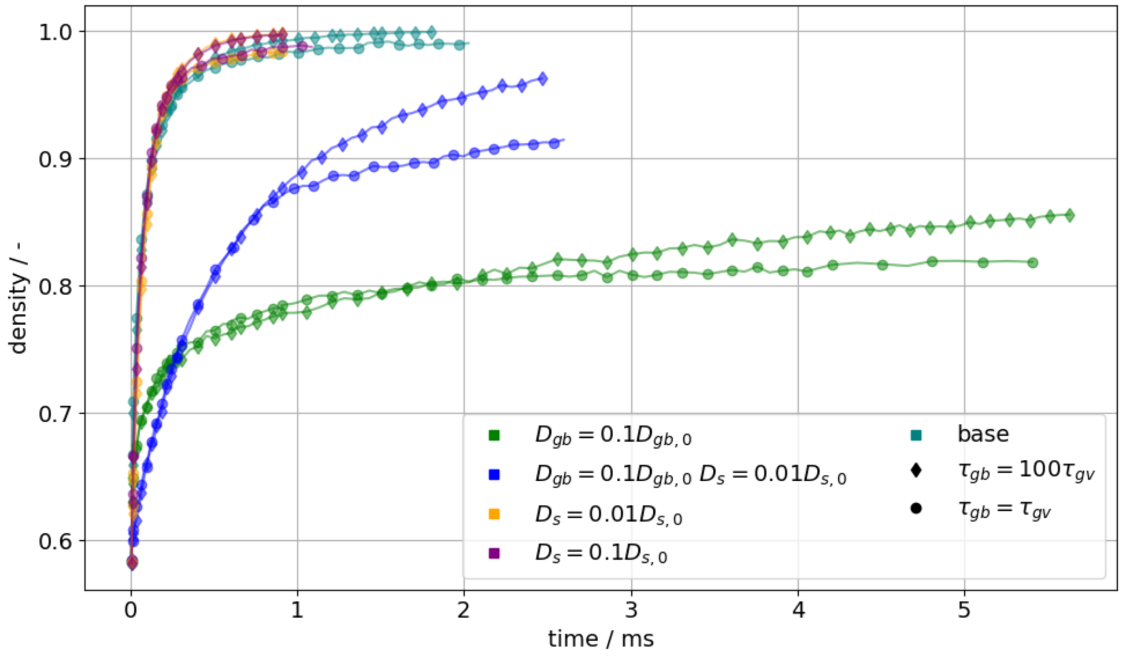

Due to grain growth's (GG) influence on the density evolution it is considered next, first in a linear plot of grain size vs. time. GG is observed for either sufficient time (green diamonds) or if no kinetic suppression of GG is done. Within the present model there is no thermodynamic way of stopping GG and hence a simple kinetic approach is chosen for simulating densification with and without GG.

In [14]:
```
fig, ax = plt.subplots()
#ax2 = ax.twinx()
trtaufac = {}
```

```
trsizen = set()
def iplot(fig, ax,  k):
    v = datdict[k]
    taufac = float(taufHandler(k, pprint=None, nodefaultprint=False))
    trtaufac[taufac] = taufHandler(k, nodefaultprint=False)
    ls = lsen[taufac]

    densdf = v["density"]
    #gsize = v["avgRadius"]
    rs = vol2r(v["volss"])
    dx = v["meta"]["dx"] #.split(" ")[-1])
    gsize = []
    gcount = []
    for frame in rs:
        filt = frame > 4
        gcount.append(np.count_nonzero(filt))
        gsize.append(np.average(frame[filt]))
    time = densdf["time"]
    dens = densdf["density"]
    filt = (dens < 1) #& (time <= 3) #& (time*t0*1e3 < 1.2*t0*1e3)
    xdat = time[filt] * t0 * 1e3
    ydat = dens[filt]

    lab = mkName_GG(k)
    # and "_tauf100_" in k
    if len(lab.replace(" ", "")) == 0: # too many autohandlers in between ma
        lab = "base"
    col = gg_colors[lab]
    mevery=2

    ydat = physSizeNm(np.array(gsize) * dx)
    ax.plot(xdat, ydat, ls.replace("-", "-"),
             color=col, alpha=0.5,
            markevery=mevery, zorder=10)


ik, ilab = [], []
for k in names_GG:
    if "dx0.1" in k and "r12" in k and "pack400" in k and "tauf10_" not in k
    "relaxf" not in k or "relaxf1_" in k) and "_D" in k:
        lab = mkName_GG(k)
        ik.append(k)
        ilab.append(lab)

tmpilab = ilab.copy()
tmpilab.append("base")
ulab = sorted(set(tmpilab), key=natkey)
gg_colors = {k: v for k, v in zip(ulab, coloursen)}
gg_colors["base"] = coloursen[-1]
ik = [x for _, x in sorted(zip(ilab, ik), key=natkey)]

iplot(fig, ax,  basesim)
iplot(fig, ax,  basesim1)

for lab, col in gg_colors.items():
    ax.plot([], [], "s", color=col, label=lab)
```

```
for k in ik:
    iplot(fig, ax, k)

for taufac, lab in trtaufac.items():
    ls = lsen[taufac]
    ax.plot([], [], ls.replace("-", ""), color="black", label=lab)

ax.set_xlabel("time / ms")
ax.set_ylabel("grain size / nm")
ax.legend(ncol=3, loc='lower center', bbox_to_anchor=(0.5, 1))

fig.tight_layout()
##fig.savefig("gg-Dvar.pdf", bbox_inches="tight")
```

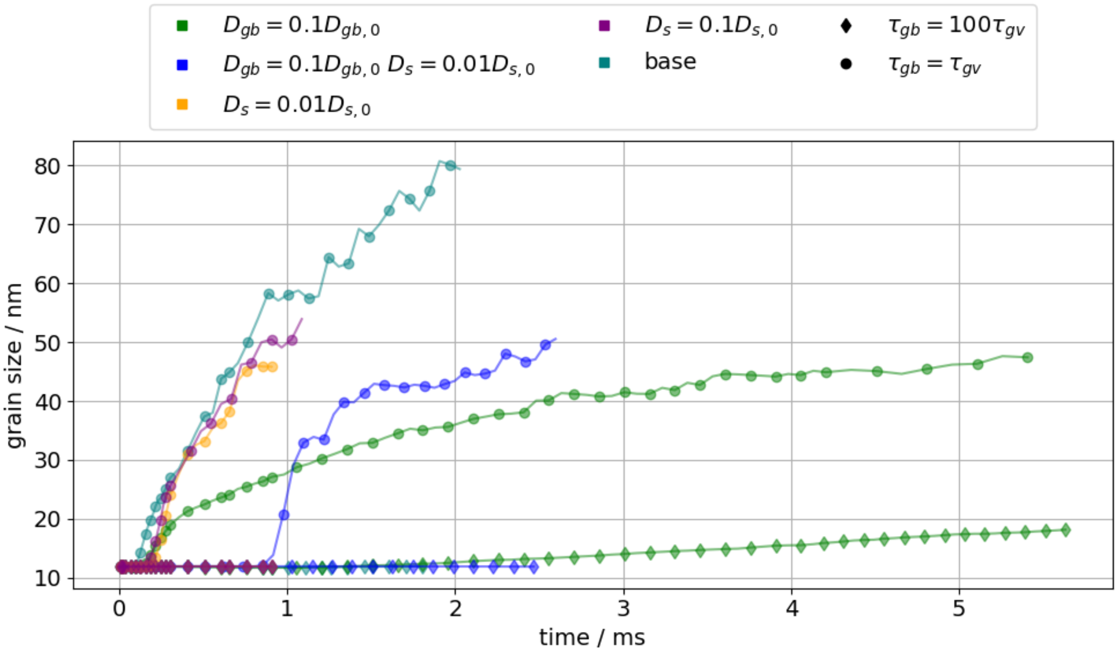

We estimate the grain growth law via log-log transform to get rid of the nonlinear fitting problem. The simulations with reduced surface diffusion generally are quite far away from slope (n) <= 0.5, with the base case (0.6) being quite a bit off as well. Reducing grain boundary diffusion puts the slope closer to 1/3, which is also sometimes observed in experiments, especially prior to the final stage of sintering.

As the grain size for filtering is increased (`minG`), the loglog-slope becomes closer to the theoretical/experimental range, but overall too few grains are contained to achieve steady-state.

In [15]:
```
mpl.rcParams.update({'font.size': 14})
fig, ax = plt.subplots(figsize=(10,5))
#ax2 = ax.twinx()
trtaufac = {}
minG = 13

trsizen = set()
```

```python
def iplot(fig, ax,  k):
    v = datdict[k]
    taufac = float(taufHandler(k, pprint=None, nodefaultprint=False))
    trtaufac[taufac] = taufHandler(k, nodefaultprint=False)
    ls = lsen[taufac]

    densdf = v["density"]
    #gsize = v["avgRadius"]
    rs = vol2r(v["volss"])
    dx = v["meta"]["dx"] #.split(" ")[-1])
    gsize = []
    gcount = []
    for frame in rs:
        filt = frame > 4
        gcount.append(np.count_nonzero(filt))
        gsize.append(np.average(frame[filt]))
    time = densdf["time"].values
    dens = densdf["density"].values
    filt = (dens < 1) #& (time <= 3) #& (time*t0*1e3 < 1.2*t0*1e3)
    xdat = time[filt] * t0 * 1e3
    ydat = dens[filt]

    lab = mkName_GG(k)
    # and "_tauf100_" in k
    if len(lab.replace(" ", "")) == 0: # too many autohandlers in between ma
        lab = "base"
    col = gg_colors[lab]
    mevery=2

    ydat = physSizeNm(np.array(gsize) * dx)
    ax.loglog(xdat, ydat, ls.replace("-", ""),
             color=col, alpha=0.5,
            markevery=mevery,)
    fitfilt = (filt) & (np.array(gsize) > minG) #& (dens > 0.95)
    if np.any(fitfilt):
        xlogdat, ylogdat = [np.log(x[fitfilt]) for x in [xdat, ydat]]
        slope, intercept, r_value, p_value, std_err = stats.linregress(xlogd
        #print(k, slope, r_value**2, p_value)
        print(lab, "has slope", slope, "with R^2", r_value**2)
        def poly(x, slope=slope, intercept=intercept):
            return x*slope + intercept

        minx, maxx = np.min(xlogdat), np.max(xlogdat)
        xxs = np.linspace(minx*0.95, maxx*1.05, xlogdat.shape[0])
        xxdat, yydat = np.exp(xxs), np.exp(poly(xxs))
        ax.plot(xxdat, yydat, "--", color=col)
        #print(lab, xdat.shape, ydat.shape, type(xdat), type(ydat))
        lpos = [xdat[-1], ydat[-1]]
        if lab == "$D_{s} = 0.1D_{s,0}$":
            lpos[0] -= 0.7
        elif lab == r"$D_{s} = 0.01D_{s,0}$" :
            lpos = [0.46, 27]
        ax.annotate(r"$n=%.2g$" % (slope), lpos, color=col)


ik, ilab = [], []
```

```
for k in names_GG:
    if "dx0.1" in k and "r12" in k and "pack400" in k and "tauf10_" not in k
    "relaxf" not in k or "relaxf1_" in k) and "_D" in k:
        lab = mkName_GG(k)
        ik.append(k)
        ilab.append(lab)

tmpilab = ilab.copy()
tmpilab.append("base")
ulab = sorted(set(tmpilab), key=natkey)
gg_colors = {k: v for k, v in zip(ulab, coloursen)}
gg_colors["base"] = coloursen[-1]
ik = [x for _, x in sorted(zip(ilab, ik), key=natkey)]

iplot(fig, ax,  basesim)
iplot(fig, ax,  basesim1)

for lab, col in gg_colors.items():
    ax.plot([], [], "s", color=col, label=lab)

# not necessary but copypasta hooray
#ilab = [x for x, _ in sorted(zip(ilab, ik), key=natkey)]
for k in ik:
    iplot(fig, ax, k)

for taufac, lab in trtaufac.items():
    ls = lsen[taufac]
    ax.plot([], [], ls.replace("-", ""), color="black", label=lab)


ax.set_xlabel("time / ms")
ax.set_ylabel("grain size / nm")


#ax.set_xlim(5e-3, 16)
#ax.set_ylim(10, 100)
ax.legend()
fig.tight_layout()

##fig.savefig("gg-loglog.pdf", bbox_inches="tight")
```

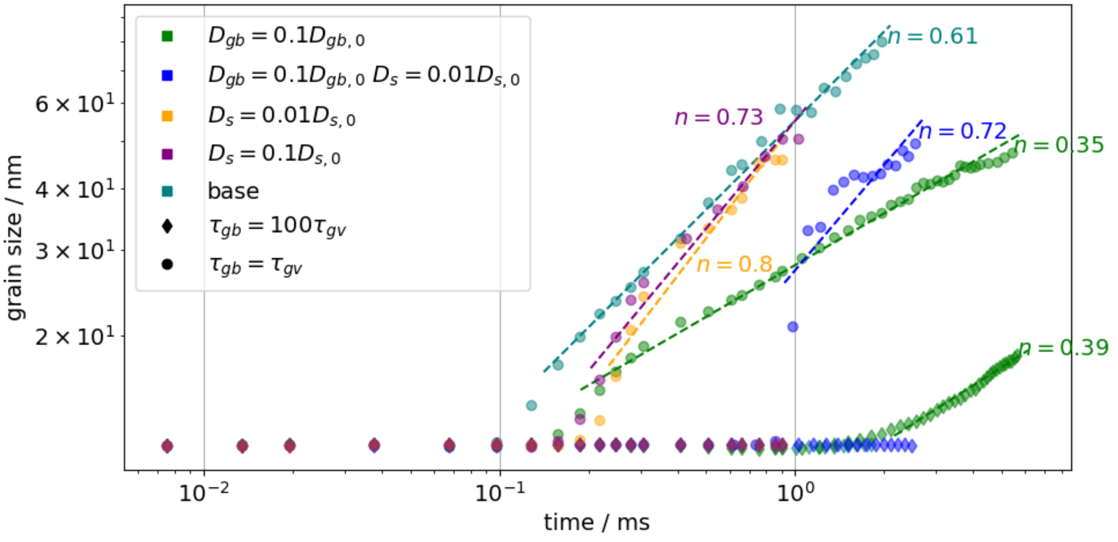


```
base has slope 0.6071411973015951 with R^2 0.9919421210658638
$D_{gb} = 0.1D_{gb,0}$ has slope 0.35291618688737547 with R^2 0.98931100819
53018
$D_{gb} = 0.1D_{gb,0}$ has slope 0.3875501079687276 with R^2 0.988939569729
605
$D_{gb} = 0.1D_{gb,0}$ $D_{s} = 0.01D_{s,0}$ has slope 0.7176250057937267 w
ith R^2 0.6745348582606038
$D_{s} = 0.01D_{s,0}$ has slope 0.7955119838526118 with R^2 0.9478704868002
227
$D_{s} = 0.1D_{s,0}$ has slope 0.7348696951429431 with R^2 0.96315015019020
76
```

One could make a detachment map from the next plot, but the sorry fact is that arbitrary parameter variation only resulted in simulations hitting straight into the detachment region or basically densifying completely prior to the onset of grain growth. The simulation with reduced GB diffusion and kinetically suppressed grain growth will also likely hit the detachment region, but since it's densifying so slowly the worth of continuing the simulation is questionable.

In [16]:
```
fig, ax = plt.subplots()
#ax2 = ax.twinx()
trtaufac = {}

trsizen = set()
def iplot(fig, ax,  k):
    v = datdict[k]
    taufac = float(taufHandler(k, pprint=None, nodefaultprint=False))
    trtaufac[taufac] = taufHandler(k, nodefaultprint=False)
    ls = lsen[taufac]

    densdf = v["density"]
    gsize = v["avgRadius"]

    time = densdf["time"]
    dens = densdf["density"]
    filt = (dens < 1) #& (time <= 3) #& (time*t0*1e3 < 1.2*t0*1e3)
    xdat = time[filt]
```

```
    ydat = dens[filt]

    lab = mkName_GG(k)
    # and "_tauf100_" in k
    if len(lab.replace(" ", "")) == 0: # too many autohandlers in between ma
        lab = "base"
    col = gg_colors[lab]
    mevery=2
    # somehow or other make color of same Dgb/Ds var same
    pl = ax.plot(ydat, gsize, ls,
        color=col,  alpha=0.5,
           markevery=mevery,
                )
           #label=lab)


ik, ilab = [], []
for k in names_GG:
    if "dx0.1" in k and "r12" in k and "pack400" in k and "tauf10_" not in k
    "relaxf" not in k or "relaxf1_" in k) and "_D" in k:
        lab = mkName_GG(k)
        ik.append(k)
        ilab.append(lab)

tmpilab = ilab.copy()
tmpilab.append("base")
ulab = sorted(set(tmpilab), key=natkey)
gg_colors = {k: v for k, v in zip(ulab, coloursen)}
gg_colors["base"] = coloursen[-1]
ik = [x for _, x in sorted(zip(ilab, ik), key=natkey)]

iplot(fig, ax,  basesim)
iplot(fig, ax,  basesim1)

for lab, col in gg_colors.items():
    ax.plot([], [], "s", color=col, label=lab)


for k in ik:
    iplot(fig, ax,  k)

for taufac, lab in trtaufac.items():
    ls = lsen[taufac]
    ax.plot([], [], ls.replace("-", ""), color="black", label=lab)



ax.set_xlabel("density / -")
ax.set_ylabel("grain size / nm")
ax.legend()
fig.tight_layout()
##fig.savefig("densi-gg-state-Dvar.pdf", bbox_inches="tight")
```

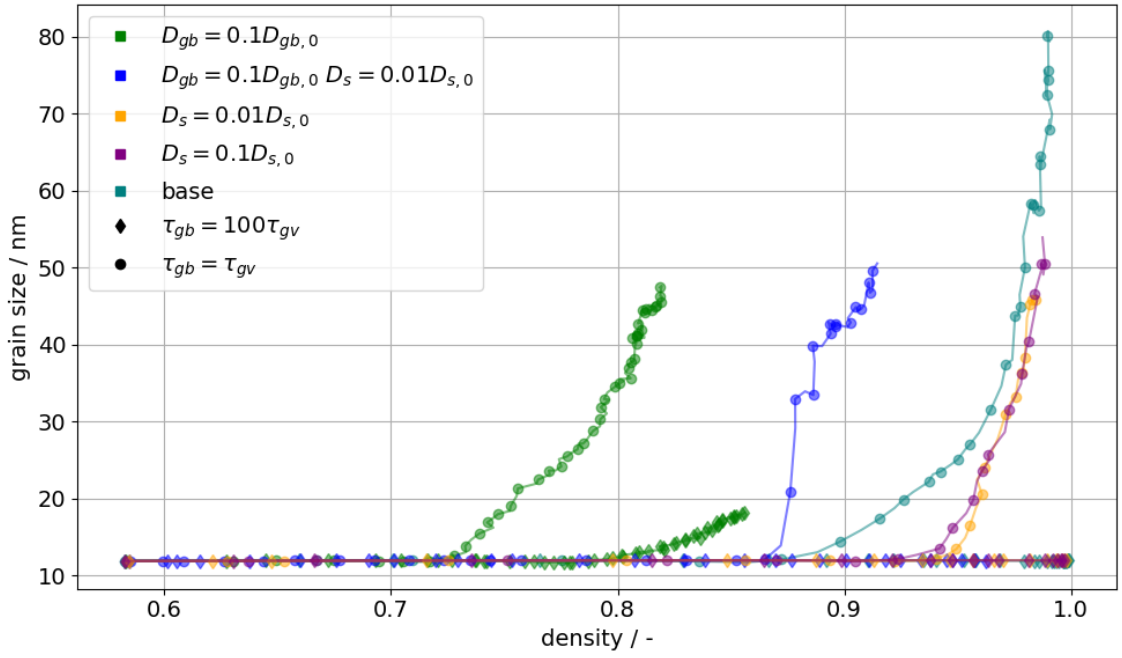

The onset of GG in earnest is easily seen in a GB area over time plot: Once the GB area reaches a maximum, massive grain growth starts. The delayed onset of GG for the blue curve is due to its GB area evolution being much slower than the rest of simulations. Since both interfacial diffusivities are reduced, mass transport to the neck is severely limited and hence the GB only grows slowly.

In [17]:
```
fig, ax = plt.subplots()
#ax2 = ax.twinx()
trtaufac = {}

trsizen = set()
def iplot(fig, ax,  k):
    #print(k)
    v = datdict[k]
    taufac = float(taufHandler(k, pprint=None, nodefaultprint=False))
    trtaufac[taufac] = taufHandler(k, nodefaultprint=False)
    ls = lsen[taufac]

    densdf = v["density"]
    gsize = v["avgRadius"]
    poro = v["isolated"]

    euler = poro["euler"]
    gba = poro["gbarea"]

    time = densdf["time"] * t0 *1e3
    dens = densdf["density"]
    filt = (dens < 1) #& (time <= 3) #& (time*t0*1e3 < 1.2*t0*1e3)
    xdat = time[filt] #time[filt]
    ydat = gba[filt] * (l0**2) * (1e6)**2 #dens[filt]

    lab = mkName_GG(k)
    # and "_tauf100_" in k
```

```
    if len(lab.replace(" ", "")) == 0: # too many autohandlers in between ma
        lab = "base"
    col = gg_colors[lab]
    mevery=1

    pl = ax.plot(xdat, ydat, ls,
        color=col,
           markevery=mevery,
                 alpha=0.5
                )
           #label=lab)


ik, ilab = [], []
for k in names_GG:
    if "dx0.1" in k and "r12" in k and "pack400" in k and "tauf10_" not in k
    "relaxf" not in k or "relaxf1_" in k) and "_D" in k:
        lab = mkName_GG(k)
        ik.append(k)
        ilab.append(lab)

tmpilab = ilab.copy()
tmpilab.append("base")
ulab = sorted(set(tmpilab), key=natkey)
gg_colors = {k: v for k, v in zip(ulab, coloursen)}
gg_colors["base"] = coloursen[-1]
ik = [x for _, x in sorted(zip(ilab, ik), key=natkey)]

iplot(fig, ax,  basesim)
iplot(fig, ax,  basesim1)

for lab, col in gg_colors.items():
    ax.plot([], [], "s", color=col, label=lab)

for k in ik:
    iplot(fig, ax,  k)

for taufac, lab in trtaufac.items():
    ls = lsen[taufac]
    ax.plot([], [], ls.replace("-", ""), color="black", label=lab)


ax.set_ylim(60, 1100) # for legend
ax.set_xlabel("time / ms")
ax.set_ylabel(r"$A_{gb}$ / $\mathrm{µm}^2$")
ax.legend(ncol=3, loc="lower center", bbox_to_anchor=(0.5, 1))
fig.tight_layout()
##fig.savefig("gbarea-time-Dvar.pdf", bbox_inches="tight")
```

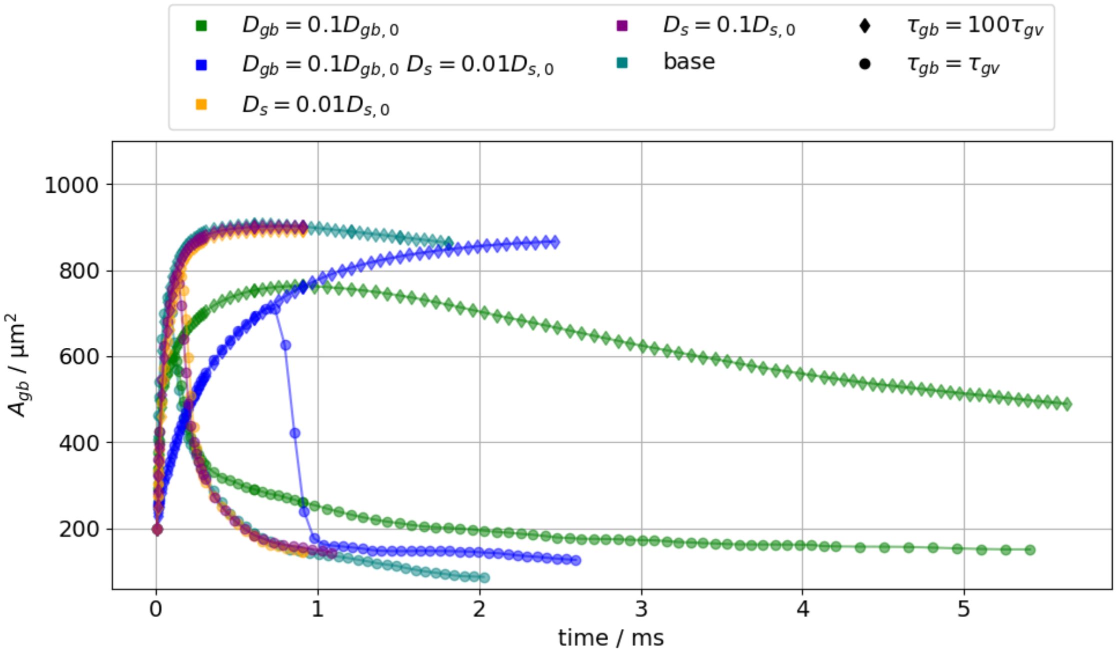

There seems to be a characteristic range of GB area divided by average coordination number (~75 to 80) after which grain growth occurs, as show below. This should not be confused with an average GB area, since that would be $A_{gb}/N(contacts)$ and not $A_{gb}/avg(contacts)$. $A_{gb}/N(contacts)$ in fact rises mostly monotonically (divide by `nzs` instead of `neighs_avg`). Why this occurs and whether this could be a simple function of particle radius is left to future work.

In [18]:
```
fig, ax = plt.subplots()
#ax2 = ax.twinx()
trtaufac = {}


trsizen = set()
def iplot(fig, ax,  k):
    #print(k)
    v = datdict[k]
    taufac = float(taufHandler(k, pprint=None, nodefaultprint=False))
    trtaufac[taufac] = taufHandler(k, nodefaultprint=False)
    ls = lsen[taufac]

    densdf = v["density"]
    gsize = v["avgRadius"]
    poro = v["isolated"]
    try:
        ssa = poro["surfarea"]
    except KeyError as e:
        print(e)
        print(k, "probably not done evaluating yet, skipping")
        return
    euler = poro["euler"]
    gba = poro["gbarea"]
```

```
    ns = v["neighss"]

    nzs = np.count_nonzero(ns, axis=1)
    neighs_avg = v["avgNeighs"]

    time = densdf["time"] * t0 *1e3
    dens = densdf["density"]
    filt = (dens < 1) #& (time <= 3) #& (time*t0*1e3 < 1.2*t0*1e3)
    xdat = time[filt] #time[filt]
    # all neighbours, including those of surface-near grains should be count
    # since gba is also based on *all* grain boundaries
    ydat = gba[filt] * (l0**2) * (1e6)**2  / neighs_avg #nzs #dens[filt]

    lab = mkName_GG(k)
    # and "_tauf100_" in k
    if len(lab.replace(" ", "")) == 0: # too many autohandlers in between ma
        lab = "base"
    col = gg_colors[lab]
    mevery=1

    pl = ax.plot(xdat, ydat, ls,
        color=col,
           markevery=mevery,
                 alpha=0.5
                )
           #label=lab)


ik, ilab = [], []
for k in names_GG:
    if "dx0.1" in k and "r12" in k and "pack400" in k and "tauf10_" not in k
    "relaxf" not in k or "relaxf1_" in k) and "_D" in k:
        lab = mkName_GG(k)
        ik.append(k)
        ilab.append(lab)

tmpilab = ilab.copy()
tmpilab.append("base")
ulab = sorted(set(tmpilab), key=natkey)
gg_colors = {k: v for k, v in zip(ulab, coloursen)}
gg_colors["base"] = coloursen[-1]
ik = [x for _, x in sorted(zip(ilab, ik), key=natkey)]

iplot(fig, ax,  basesim)
iplot(fig, ax,  basesim1)

for lab, col in gg_colors.items():
    ax.plot([], [], "s", color=col, label=lab)


for k in ik:
    iplot(fig, ax,  k)

for taufac, lab in trtaufac.items():
    ls = lsen[taufac]
    ax.plot([], [], ls.replace("-", ""), color="black", label=lab)
```

```
#ax.set_ylim(60, 1100) # for legend
ax.set_xlabel("time / ms")
ax.set_ylabel(r"$A_{gb}$ divided by avg($N_c$) / $\mathrm{µm}^2$")
ax.legend(ncol=3, loc="lower center", bbox_to_anchor=(0.5, 1))
fig.tight_layout()
##fig.savefig("gbarea-time-Dvar.pdf", bbox_inches="tight")
```

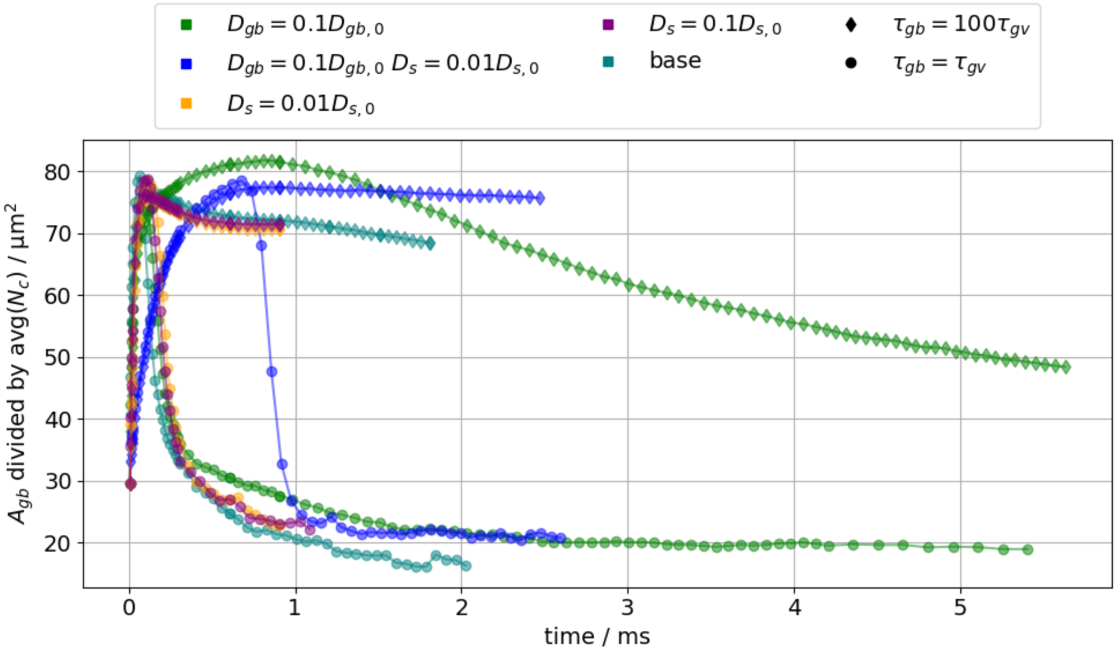

The difference between slow and fast surface diffusion when densification is slow can also be understood by observing the curvature at the neck region--- fast surface diffusion will reduce it much faster and therefore eliminate further driving force for densification. Based on the simulation observations, at the same density, a larger neck curvature is observed for when surface diffusion is slow as well as when grain boundary diffusion is high (cf. blue/purple)

The neck region is simply defined as where there is at least certain amount of surface phase $\phi_S = 4\phi_V(1 - \phi_V) > 0.01$ and if more than two grains are present --- i.e. it is a subregion of the surface region. The mean curvature H within this region is evaluated by the standard $\nabla \cdot \vec{n} = H$ formula, with the normal vector being that of the vapor phase-field ϕ_V. The average curvature is calculated with a weighting by ϕ_S. The choice of cutoff doesn't affect the shape of the curve, but rather mostly the values, which tend to be slightly more positive for higher cutoffs.

The neck curvature becoming more negative for a significant density regime is due to a decrease of neck surface area, shown in the next plot.

In [19]:
```
fig, ax = plt.subplots()
#ax2 = ax.twinx()
trtaufac = {}
```

```
# for switching curvature/radius plotting
plotk = True
minps = {}
trsizen = set()
def iplot(fig, ax,  k):
    v = datdict[k]
    taufac = float(taufHandler(k, pprint=None, nodefaultprint=False))
    trtaufac[taufac] = taufHandler(k, nodefaultprint=False)
    ls = lsen[taufac]

    df = v["ns"]
    densdf = v["density"]

    time = df["time"]
    dens = densdf["density"]
    # int(H*w, dV) / int(w, dV)
    kt = df["kt"] / df["wt"]
    filt = time < np.inf #(dens < 1) #& (time <= 3) #& (time*t0*1e3 < 1.2*t0
    xdat = dens[filt]
    ydat = kt[filt]
    minp = np.argmin(ydat)
    minps[k] = dens[minp]

    lab = mkName_GG(k)
    # and "_tauf100_" in k
    if len(lab.replace(" ", "")) == 0: # too many autohandlers in between ma
        lab = "base"
    col = gg_colors[lab]
    mevery=2

    if plotk:
        pl = ax.plot(xdat, ydat/(l0 * 1e9), ls,
            color=col, alpha=0.5,
               markevery=mevery,
                     #markersize=10
                    )

    else:
        pl = ax.plot(xdat, 1/ydat * (l0 * 1e9), ls,
            color=col, alpha=0.5,
               markevery=mevery,
                     #markersize=10
                    )


ik, ilab = [], []
for k in names_GG:
    if "dx0.1" in k and "r12" in k and "pack400" in k and "tauf10_" not in k
    "relaxf" not in k or "relaxf1_" in k) and "_D" in k:
        lab = mkName_GG(k)
        ik.append(k)
        ilab.append(lab)

tmpilab = ilab.copy()
tmpilab.append("base")
ulab = sorted(set(tmpilab), key=natkey)
```

```
gg_colors = {k: v for k, v in zip(ulab, coloursen)}
gg_colors["base"] = coloursen[-1]
ik = [x for _, x in sorted(zip(ilab, ik), key=natkey)]

iplot(fig, ax,  basesim)
iplot(fig, ax,  basesim1)

for lab, col in gg_colors.items():
    ax.plot([], [], "s", color=col, label=lab)


for k in ik:
    iplot(fig, ax,  k)

for taufac, lab in trtaufac.items():
    ls = lsen[taufac]
    ax.plot([], [], ls.replace("-", ""), color="black", label=lab)


ax.set_xlabel("density / -")
if plotk:
    ax.set_ylabel("average neck mean curvature / $(\mathrm{nm})^{-1}$")
else:
    ax.set_ylabel("effective radius of curvature at the neck / $\mathrm{nm}$

ax.legend(ncol=2)
fig.tight_layout()
#fig.savefig("neckcurvature.pdf", bbox_inches="tight")
```

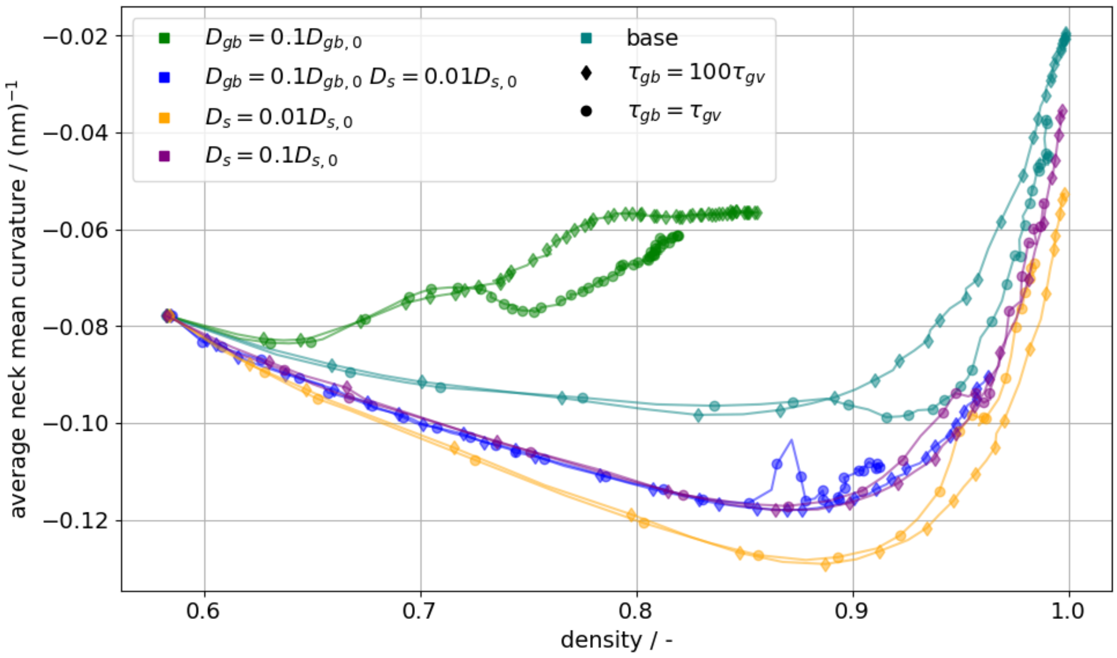

In [20]:
```
fig, ax = plt.subplots()
#ax2 = ax.twinx()
trtaufac = {}
trsizen = set()
def iplot(fig, ax,  k):
```

```
    v = datdict[k]
    taufac = float(taufHandler(k, pprint=None, nodefaultprint=False))
    trtaufac[taufac] = taufHandler(k, nodefaultprint=False)
    ls = lsen[taufac]

    df = v["ns"]
    densdf = v["density"]

    time = df["time"]
    dens = densdf["density"]
    kt = df["wt"] #/ df["wt"] # wt -> kt and the integral of (H*phi_s) is pl
    filt = time < np.inf #(dens < 1) #& (time <= 3) #& (time*t0*1e3 < 1.2*t0
    xdat = dens[filt]
    ydat = kt[filt]

    lab = mkName_GG(k)
    # and "_tauf100_" in k
    if len(lab.replace(" ", "")) == 0: # too many autohandlers in between ma
        lab = "base"
    col = gg_colors[lab]
    mevery=2

    # not dimensional here because strictly speaking the triple region is a
    # so there would be some geometric assumptions necessary to make this a
    # however, these would only affect the scale of the plot, not its trend
    pl = ax.plot(xdat, ydat, ls,
        color=col, alpha=0.5,
           markevery=mevery,
                 #markersize=10
                )


ik, ilab = [], []
for k in names_GG:
    if "dx0.1" in k and "r12" in k and "pack400" in k and "tauf10_" not in k
    "relaxf" not in k or "relaxf1_" in k) and "_D" in k:
        lab = mkName_GG(k)
        ik.append(k)
        ilab.append(lab)

tmpilab = ilab.copy()
tmpilab.append("base")
ulab = sorted(set(tmpilab), key=natkey)
gg_colors = {k: v for k, v in zip(ulab, coloursen)}
gg_colors["base"] = coloursen[-1]
ik = [x for _, x in sorted(zip(ilab, ik), key=natkey)]

iplot(fig, ax,  basesim)
iplot(fig, ax,  basesim1)

for lab, col in gg_colors.items():
    ax.plot([], [], "s", color=col, label=lab)


for k in ik:
    iplot(fig, ax,  k)
```

```
for taufac, lab in trtaufac.items():
    ls = lsen[taufac]
    ax.plot([], [], ls.replace("-", ""), color="black", label=lab)


ax.set_xlabel("density / -")

ax.set_ylabel("neck surface area / -")

ax.legend(ncol=2)
fig.tight_layout()
##fig.savefig("necksurfarea.pdf", bbox_inches="tight")
```

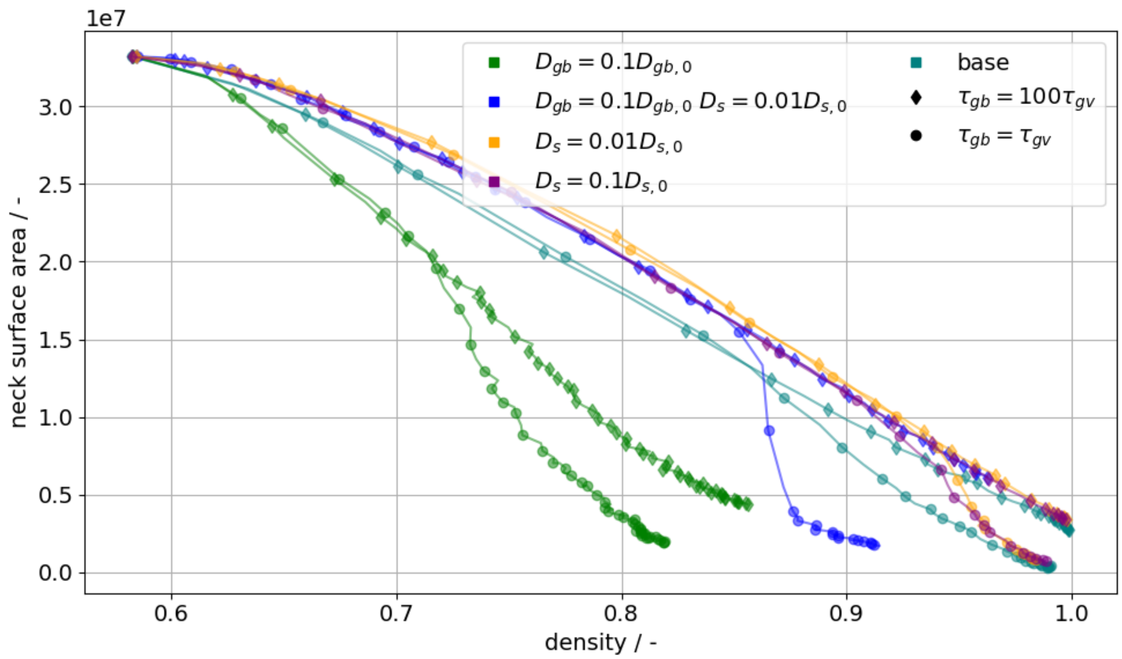

And in general, the free energy of the simulations is monotonically decreasing. The free energy is simply the evaluation of the functional given in the derivation.

In [21]:
```
fig, ax = plt.subplots()
#ax2 = ax.twinx()
trtaufac = {}
trsizen = set()
def iplot(fig, ax,  k):
    try:
        v = datdict[k]
        taufac = float(taufHandler(k, pprint=None, nodefaultprint=False))
        trtaufac[taufac] = taufHandler(k, nodefaultprint=False)
        ls = lsen[taufac]

        df = v["ns"]
        densdf = v["density"]

        time = df["time"]
        dens = densdf["density"]
```

```
        edf = v["energy"]
        energy = edf["energy"]
        time = edf["time"]
        filt = time < np.inf #(dens < 1) #& (time <= 3) #& (time*t0*1e3 < 1.
        xdat = dens[filt] # change this to time[filt] to plot over time
        ydat = energy[filt]

        lab = mkName_GG(k)
        # and "_tauf100_" in k
        if len(lab.replace(" ", "")) == 0: # too many autohandlers in betwee
            lab = "base"
        col = gg_colors[lab]
        mevery=2

        de = np.diff(ydat)
        if np.any(de > 0):
            print(k, " increases free energy at at least one point")
        pl = ax.plot(xdat, ydat, ls,
            color=col, alpha=0.5,
               markevery=mevery,
                     #markersize=10
                 )
    except:
        pass



ik, ilab = [], []
for k in names_GG:
    if "dx0.1" in k and "r12" in k and "pack400" in k and "tauf10_" not in k
    "relaxf" not in k or "relaxf1_" in k) and "_D" in k:
        lab = mkName_GG(k)
        ik.append(k)
        ilab.append(lab)

tmpilab = ilab.copy()
tmpilab.append("base")
ulab = sorted(set(tmpilab), key=natkey)
gg_colors = {k: v for k, v in zip(ulab, coloursen)}
gg_colors["base"] = coloursen[-1]
ik = [x for _, x in sorted(zip(ilab, ik), key=natkey)]

iplot(fig, ax,  basesim)
iplot(fig, ax,  basesim1)

for lab, col in gg_colors.items():
    ax.plot([], [], "s", color=col, label=lab)


for k in ik:
    iplot(fig, ax,  k)

for taufac, lab in trtaufac.items():
    ls = lsen[taufac]
    ax.plot([], [], ls.replace("-", ""), color="black", label=lab)
```

```
ax.set_xlabel("density / -")
ax.set_ylabel("free energy / - ")

ax.legend(ncol=2)
fig.tight_layout()
#fig.savefig("energy.pdf", bbox_inches="tight")
```

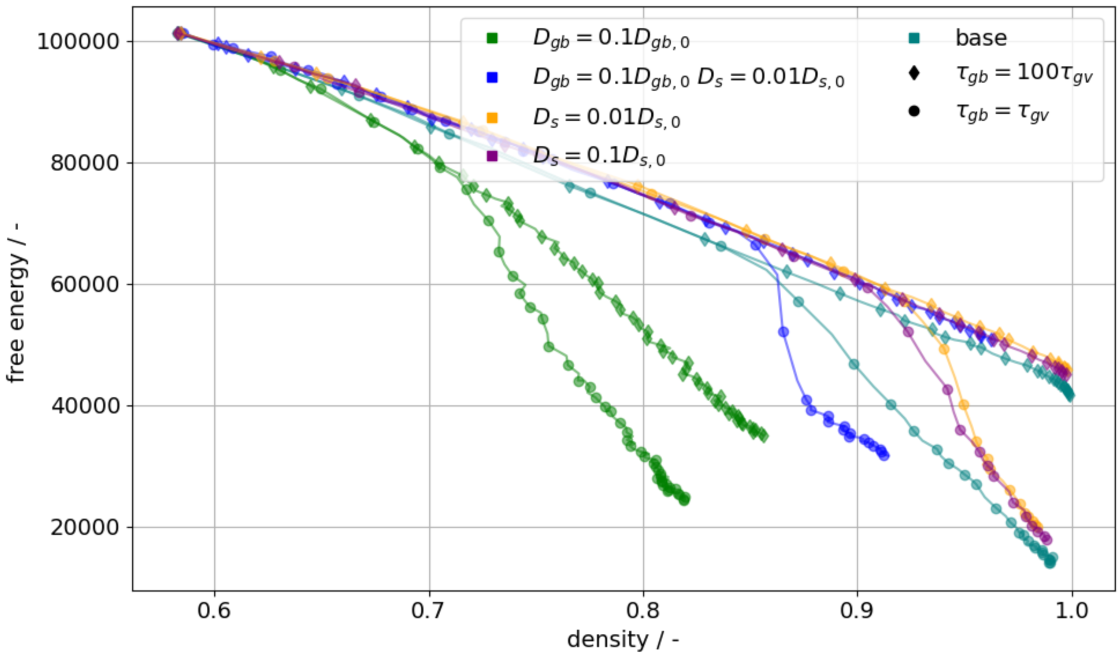

This coordination number - density plot nicely shows when grain growth becomes affected by finite size effects, as the coordination number should increase monotonically with density.

NB: This filters out surface-near grains, but not grain contacts with particularly small contact areas. Whether these represent "true" neighbours or not is not considered and hence they are simply counted as well.

In [22]:
```
fig, ax = plt.subplots()
#ax2 = ax.twinx()
trtaufac = {}


trsizen = set()
def iplot(fig, ax,  k):
    v = datdict[k]
    taufac = float(taufHandler(k, pprint=None, nodefaultprint=False))
    trtaufac[taufac] = taufHandler(k, nodefaultprint=False)
    ls = lsen[taufac]

    densdf = v["density"]
    gsize = v["avgRadius"]

    ns = v["neighss"]
    coms = v["comss"]
    inner_ns = removeSurfaceGrains(ns, coms)
```

```
    # since it's an object array, can't do it nicely
    neighs_avg = []
    for fr in range(inner_ns.shape[0]):
        ins = inner_ns[fr]
        neighs_avg.append(np.average(ins))
    neighs_avg = np.array(neighs_avg)
    #nsum = np.sum(neighs[gsize>4], axis=1)

    time = densdf["time"] * t0 *1e3
    dens = densdf["density"]
    filt = (dens < 1) #& (time <= 3) #& (time*t0*1e3 < 1.2*t0*1e3)
    xdat = dens[filt] #gsize[filt] #time[filt] #time[filt]
    ydat = neighs_avg[filt] #(gba / nsum)[filt] * (l0**2) * (1e6)**2 #dens[f

    lab = mkName_GG(k)
    # and "_tauf100_" in k
    if len(lab.replace(" ", "")) == 0: # too many autohandlers in between ma
        lab = "base"
    col = gg_colors[lab]
    mevery=1

    pl = ax.plot(xdat, ydat, ls,
        color=col,
           markevery=mevery,
                 alpha=0.5
                )
           #label=lab)


ik, ilab = [], []
for k in names_GG:
    if "dx0.1" in k and "r12" in k and "pack400" in k and "tauf10_" not in k
    "relaxf" not in k or "relaxf1_" in k) and "_D" in k:
        lab = mkName_GG(k)
        ik.append(k)
        ilab.append(lab)

tmpilab = ilab.copy()
tmpilab.append("base")
ulab = sorted(set(tmpilab), key=natkey)
gg_colors = {k: v for k, v in zip(ulab, coloursen)}
gg_colors["base"] = coloursen[-1]
ik = [x for _, x in sorted(zip(ilab, ik), key=natkey)]

iplot(fig, ax,  basesim)
iplot(fig, ax,  basesim1)

for lab, col in gg_colors.items():
    ax.plot([], [], "s", color=col, label=lab)


for k in ik:
    iplot(fig, ax,  k)

for taufac, lab in trtaufac.items():
    ls = lsen[taufac]
```

```
    ax.plot([], [], ls.replace("-", ""), color="black", label=lab)


densen = np.linspace(0.57, 1, 50)
ncs = 2+11*densen**2
ax.plot(densen, ncs, label="German's fit", linewidth=5, alpha=0.25, color="b
ax.set_xlabel("density / -")
ax.set_ylabel(r"avg. $N_c$ / -") # coord number

ax.legend(ncol=2)
fig.tight_layout()
```

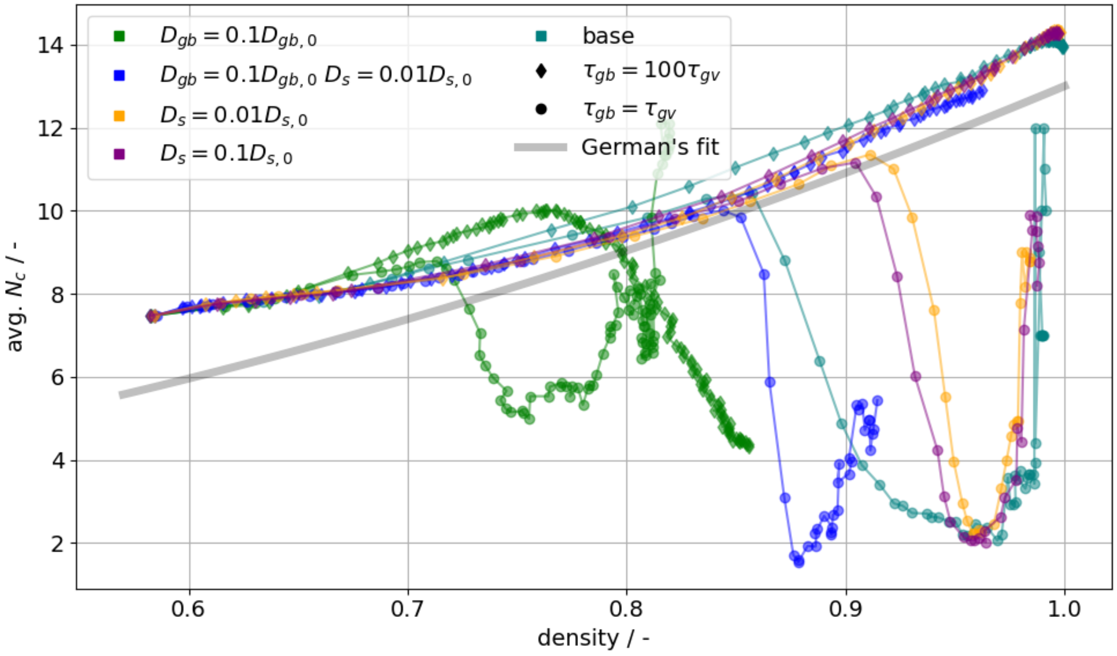

The fraction of detached porosity for slow densification shows some odd behaviour, in that the fraction *decreases* with density. However, since isolated porosity is still being generated as some pores detach this is still sensible.

In [23]:
```
mpl.rcParams.update()
fig, ax = plt.subplots()

trtaufac = {}

trsizen = set()
def iplot(fig, ax,  k):
    v = datdict[k]
    taufac = float(taufHandler(k, pprint=None, nodefaultprint=False))
    trtaufac[taufac] = taufHandler(k, nodefaultprint=False)
    ls = lsen[taufac]

    densdf = v["density"]
    gsize = v["avgRadius"]
    poro = v["isolated"]
    try:
        ssa = poro["surfarea"]
```

```
    except KeyError as e:
        print(e)
        print(k, "probably not done evaluating yet, skipping")
        return
    euler = poro["euler"]
    isum = datdict[k]["density"]["isum"] # sum(isolated) via CCM of phiv > 0
    # poro = 1-dens
    # poro = isolated + open
    # isolated = 1 - sum(grain) / (sum(grain) + sum(isolated_pores))
    tporo = 1-densdf["density"]
    iporo = 1-densdf["gsum"] / (densdf["gsum"] + densdf["isum"])
    oporo = tporo-iporo
    dporo = poro["dropped"] / poro["isolated"]
    #iporo = 1-(densdat["gsum"]/(densdat["gsum"]+densdat["isum"]))
    #oporo = poro-iporo


    time = densdf["time"]
    dens = densdf["density"]
    filt = (dens < 1) #& (time <= 3) #& (time*t0*1e3 < 1.2*t0*1e3)
    xdat = (dens)[filt] #dens[filt] #time[filt]
    #xdat = time[filt]
    ydat = euler[filt] #dens[filt]

    lab = mkName_GG(k)
    # and "_tauf100_" in k
    if len(lab.replace(" ", "")) == 0: # too many autohandlers in between ma
        lab = "base"
    col = gg_colors[lab]
    mevery=2

    ax.plot(xdat, (dporo)[filt], ls, color=col, alpha=0.5, markevery=5)
    #ax.plot(xdat, (oporo)[filt], ls, color=col, alpha=0.5, markevery=5 )


ik, ilab = [], []
for k in names_GG:
    if "dx0.1" in k and "r12" in k and "pack400" in k and "tauf1_" in k and
    "relaxf" not in k or "relaxf1_" in k) and "_D" in k:
        lab = mkName_GG(k)
        ik.append(k)
        ilab.append(lab)

tmpilab = ilab.copy()
tmpilab.append("base")
ulab = sorted(set(tmpilab), key=natkey)
gg_colors = {k: v for k, v in zip(ulab, coloursen)}
gg_colors["base"] = coloursen[-1]
ik = [x for _, x in sorted(zip(ilab, ik), key=natkey)]

#iplot(fig, ax,  basesim)
iplot(fig, ax,  basesim1)

for lab, col in gg_colors.items():
    ax.plot([], [], "s", color=col, label=lab)
```

```
for k in ik:
    iplot(fig, ax, k)

for taufac, lab in trtaufac.items():
    ls = lsen[taufac]
    ax.plot([], [], ls.replace("-", ""), color="black", label=lab)



ax.set_xlim(0.57,1.01)
ax.set_xlabel("density / -")
ax.set_ylabel(r"frac. detached porosity / -")

ax.legend(ncol=2)
fig.tight_layout()
```

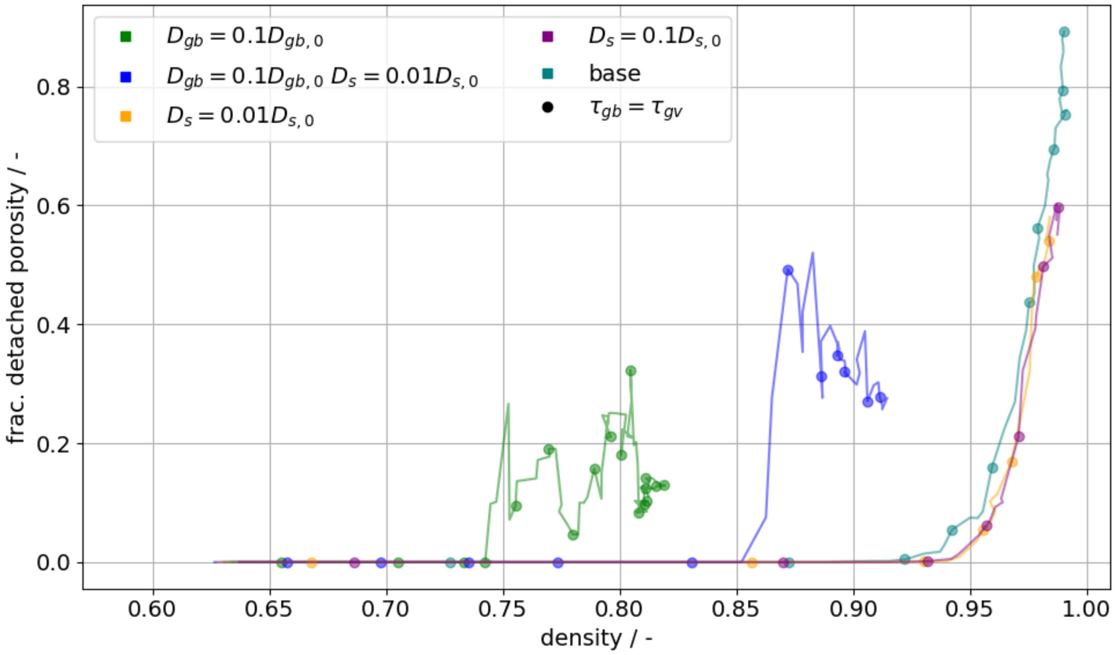

Next up are the comparisons with generally observed relationships during sintering: Grain size should be predictable via $1/\sqrt{P}$ (porosity $P = 1 - \rho$) and surface area should decrease linearly with density.

The following excludes simulations without sufficient grain growth defined via $G < 13$ nm. Hence all the diamonds, except for the green one, vanish. The simulation with reduced grain boundary diffusion was run sufficiently long to show grain growth even with kinetic suppression.

In [24]:
```
mpl.rcParams.update({'font.size': 14})
fig, ax = plt.subplots(figsize=(10,6))
#ax2 = ax.twinx()
trtaufac = {}
```

```python
trsizen = set()
def iplot(fig, ax,  k):
    v = datdict[k]
    taufac = float(taufHandler(k, pprint=None, nodefaultprint=False))


    densdf = v["density"]
    gsize = v["avgRadius"]

    time = densdf["time"]
    dens = densdf["density"]
    filt = (dens < 0.99) #& (time <= 3) #& (time*t0*1e3 < 1.2*t0*1e3)
    fitfilt = filt & (gsize > 13)
    if np.all(~fitfilt):
        return
    trtaufac[taufac] = taufHandler(k, nodefaultprint=False)
    ls = lsen[taufac]

    lab = mkName_GG(k)
    # and "_tauf100_" in k
    if len(lab.replace(" ", "")) == 0: # too many autohandlers in between ma
        lab = "base"
    col = gg_colors[lab]
    mevery=1
    xdat_all = 1/np.sqrt(1-dens)
    ydat_all = gsize
    slope, intercept, r_value, p_value, std_err = stats.linregress(xdat_all[
    #print(k, slope, r_value**2, p_value)
    print(lab, "has slope", slope, "with R^2", r_value**2)
    def poly(x, slope=slope, intercept=intercept):
        return x*slope + intercept

    minx, maxx = np.min(xdat_all[fitfilt]), np.max(xdat_all[fitfilt])
    xxs = np.linspace(minx*0.98, maxx*1.02+0.1, xdat_all.shape[0])


    # somehow or other make color of same Dgb/Ds var same
    pl = ax.plot(xdat_all[filt], gsize[filt], ls.replace("-", ""),
        color=col,  alpha=0.4,
           markevery=mevery,
                )
           #label=lab)
    ax.plot(xxs, poly(xxs), "--", color=col)


ik, ilab = [], []
for k in names_GG:
    #and "tauf100_" not in k
    if "dx0.1" in k and "r12" in k and "pack400" in k  and (
    "relaxf" not in k or "relaxf1_" in k) and "_D" in k:
        lab = mkName_GG(k)
        ik.append(k)
        ilab.append(lab)

tmpilab = ilab.copy()
```

```
tmpilab.append("base")
ulab = sorted(set(tmpilab), key=natkey)
gg_colors = {k: v for k, v in zip(ulab, coloursen)}
gg_colors["base"] = coloursen[-1]
ik = [x for _, x in sorted(zip(ilab, ik), key=natkey)]

iplot(fig, ax,  basesim)
iplot(fig, ax,  basesim1)

for lab, col in gg_colors.items():
    ax.plot([], [], "s", color=col, label=lab)


for k in ik:
    iplot(fig, ax,  k)

for taufac, lab in trtaufac.items():
    ls = lsen[taufac]
    ax.plot([], [], ls.replace("-", ""), color="black", label=lab)



ax.set_xlabel(r"$\frac{1}{\sqrt{P}}$ / -" )
ax.set_ylabel("grain size / nm")
ax.legend(ncol=2)
fig.tight_layout()

##fig.savefig("porosity-grainsize.pdf", bbox_inches="tight")
```

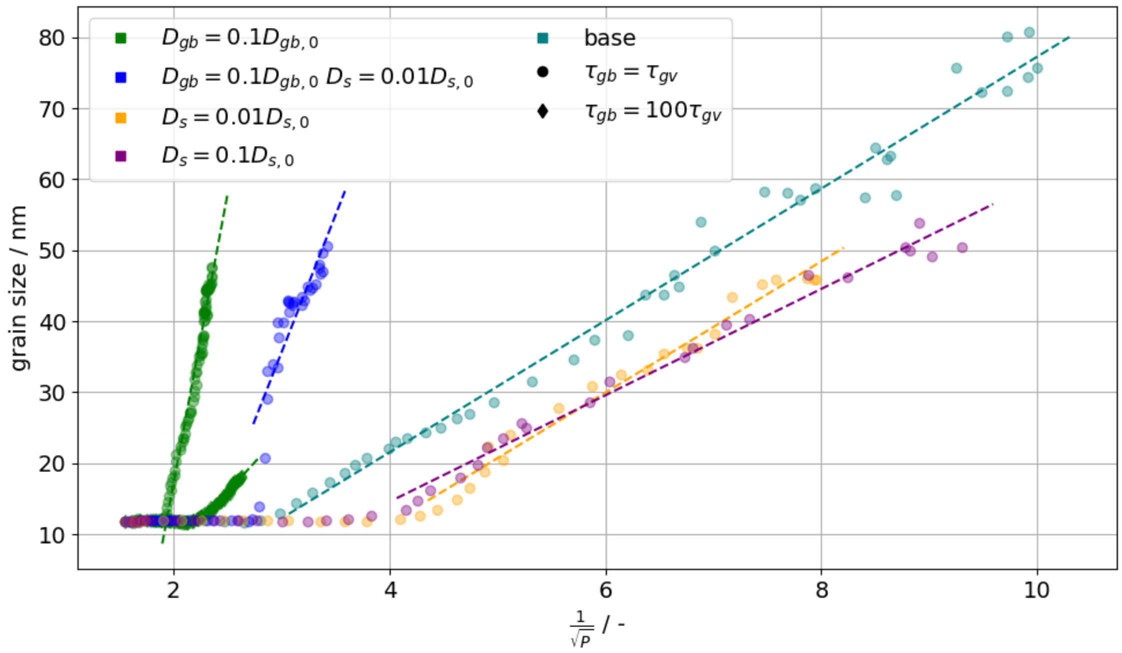

```
base has slope 9.28770760050491 with R^2 0.9832855452553578
$D_{gb} = 0.1D_{gb,0}$ has slope 81.43672949181142 with R^2 0.9703048631241
811
$D_{gb} = 0.1D_{gb,0}$ has slope 16.5772737331943 with R^2 0.97465411851794
87
$D_{gb} = 0.1D_{gb,0}$ $D_{s} = 0.01D_{s,0}$ has slope 38.65924147604448 wi
th R^2 0.7528165919811572
$D_{s} = 0.01D_{s,0}$ has slope 9.242302508088294 with R^2 0.97515919049329
14
$D_{s} = 0.1D_{s,0}$ has slope 7.495337426634094 with R^2 0.982595036416822
6
```

We can compare the trends from the previous plot to experimental data (https://doi.org/10.1007/s11837-015-1795-8 and https://doi.org/10.1016/j.scriptamat.2007.03.030) for various materials and processing conditions. Some of these match quite well and thus the model is capable of producing realistic sintering trajectories.

In [25]:
```
# digitized data from German2016 and Granger2007
german_copper_gs = pd.read_csv("data/copper-trajectory-german-poro-grainsize
                              delim_whitespace=True)
german_zirconia_gs = pd.read_csv("data/zirconia-trajectory-german-poro-grain
                              delim_whitespace=True)
al = pd.read_csv("data/alumina01-trajectory-german-poro-grainsize.dat",
                              delim_whitespace=True)
fe = pd.read_csv("data/femixed-trajectory-german-poro-grainsize.dat",
                              delim_whitespace=True)
steel = pd.read_csv("data/steel-trajectory-german-poro-grainsize.dat",
                              delim_whitespace=True)

y2o3_granger = pd.read_csv("data/y2o3-trajectory-granger-poro-gransize.dat",
```

In [27]:
```
#mpl.rcParams.update({'font.size': 24})
fig, ax = plt.subplots() #(figsize=(12,6))
#ax2 = ax.twinx()
trtaufac = {}
tfacs = [1, 10, 100]
tfacs_ls = ["-o", "-s", "-d"]
lsen = {float(k): v for k,v in zip(tfacs, tfacs_ls)}

#coloursen.reverse() # leibniz

trsizen = set()
def iplot(fig, ax,  k):
    v = datdict[k]
    taufac = float(taufHandler(k, pprint=None, nodefaultprint=False))


    densdf = v["density"]
    gsize = v["avgRadius"]

    time = densdf["time"]
    dens = densdf["density"]
    filt = (dens < 0.99) #& (time <= 3) #& (time*t0*1e3 < 1.2*t0*1e3)
```

```
    fitfilt = filt & (gsize > 13)
    if np.all(~fitfilt):
        return
    trtaufac[taufac] = taufHandler(k, nodefaultprint=False)
    ls = lsen[taufac]

    lab = mkName_GG(k)
    # and "_tauf100_" in k
    if len(lab.replace(" ", "")) == 0: # too many autohandlers in between ma
        lab = "base"
    col = gg_colors[lab]
    mevery=1
    xdat_all = 1/np.sqrt(1-dens)
    gsize = gsize / gsize[0]
    ydat_all = gsize
    slope, intercept, r_value, p_value, std_err = stats.linregress(xdat_all[
    #print(k, slope, r_value**2, p_value)
    print(lab, "has slope", slope, "with R^2", r_value**2)
    def poly(x, slope=slope, intercept=intercept):
        return x*slope + intercept

    minx, maxx = np.min(xdat_all[fitfilt]), np.max(xdat_all[fitfilt])
    xxs = np.linspace(minx*0.98, maxx*1.02+0.1, xdat_all.shape[0])


    # somehow or other make color of same Dgb/Ds var same
    #pl = ax.plot(xdat_all[filt], gsize[filt], ls.replace("-", ""),
    #    color=col,  alpha=0.4,
    #       markevery=mevery,
    #            )
    #       #label=lab)
    ax.plot(xxs, poly(xxs), "--", color=col)


ik, ilab = [], []
for k in names_GG:
    #and "tauf100_" not in k
    if "dx0.1" in k and "r12" in k and "pack400" in k  and (
    "relaxf" not in k or "relaxf1_" in k) and "_D" in k:
        lab = mkName_GG(k)
        ik.append(k)
        ilab.append(lab)

tmpilab = ilab.copy()
tmpilab.append("base")
ulab = sorted(set(tmpilab), key=natkey)
gg_colors = {k: v for k, v in zip(ulab, coloursen)}
gg_colors["base"] = coloursen[-1]
ik = [x for _, x in sorted(zip(ilab, ik), key=natkey)]

iplot(fig, ax,  basesim)
iplot(fig, ax,  basesim1)

for lab, col in gg_colors.items():
    ax.plot([], [], "s", color=col, label=lab)
```

```
# not necessary but copypasta hooray
#ilab = [x for x, _ in sorted(zip(ilab, ik), key=natkey)]
for k in ik:
    iplot(fig, ax,  k)

for taufac, lab in trtaufac.items():
    ls = lsen[taufac]
    ax.plot([], [], ls.replace("-", ""), color="black", label=lab)
#ax2.plot([], [], "-", color="black", label="density", fillstyle="none")
#ax2.plot([], [], "--", color="black", label="grain size", fillstyle="none")

#ax2.set_ylabel("grain size / nm")
#ax2.legend(loc="upper right", ncol=2)
for dat, lab in zip([german_copper_gs, german_zirconia_gs, al, fe, steel,
                     y2o3_granger], ["Cu", "ZrO_2", "Al_2O_3", "Fe", "steel"
                                                        "Y_2O_3"]):
    s0 = 80*len("Cu")
    s = s0 * len(lab) / len("Cu")
    maxpts = 8
    npts = dat["invporo"].shape[0]
    idat = dat.copy()
    g0 = dat["grainsize"][0]
    # NB we don't plot all points because these overlap too much, but instea
    # we don't care about the exact point selection because the model is goo
    if npts > maxpts:
        idat = dat.sample(n=maxpts)
    ax.scatter(idat["invporo"], idat["grainsize"] / g0,
               marker=r'$\mathrm{%s}$' % (lab), s=s, color="black", label=la
              alpha=0.5)

#ax.set_xlabel(r"$1/sqrt(1-rho)$ / -")
ax.set_xlabel(r"$\frac{1}{\sqrt{P}}$ / -" )
ax.set_ylabel(r"$\frac{G(t)}{G(0)}$ / -")
#ax.legend(ncol=1, loc='center left', bbox_to_anchor=(1.15, 0.5))
#ax.legend(ncol=3, loc='upper center', bbox_to_anchor=(0.5, 1.25))
#ax.legend(ncol=2)
fig.tight_layout()
#ax.grid(False)
#fig.savefig("porosity-grainsize-normalized-exp.pdf", bbox_inches="tight")
```

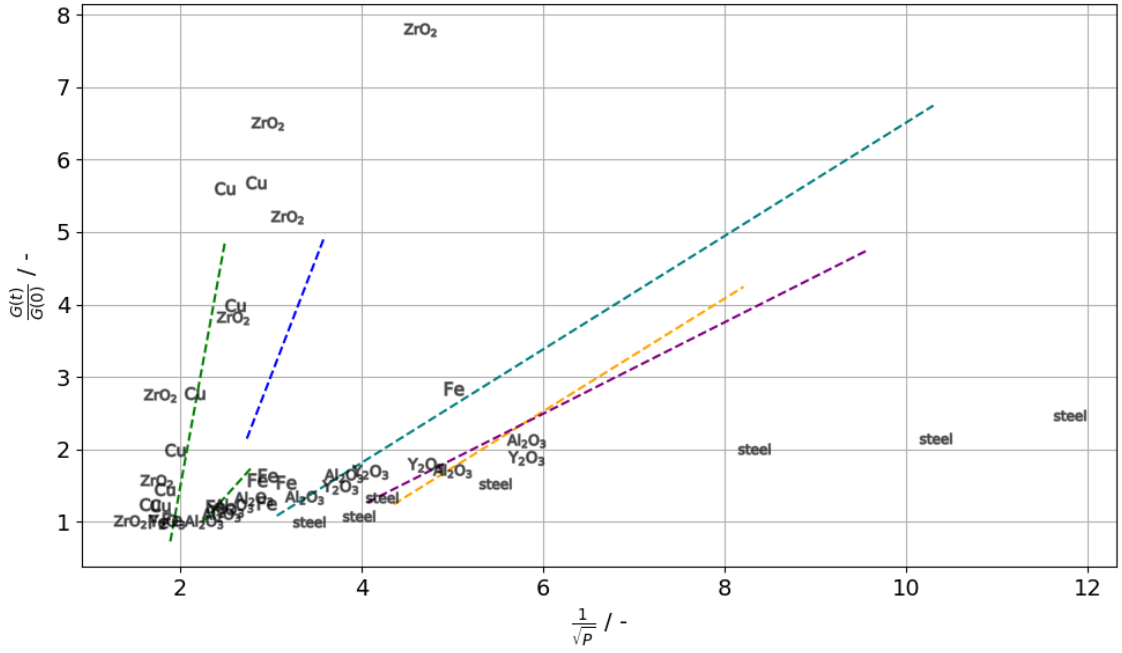


```
base has slope 0.7827987152404743 with R^2 0.983285545255358
$D_{gb} = 0.1D_{gb,0}$ has slope 6.863756909843977 with R^2 0.9703048631241
806
$D_{gb} = 0.1D_{gb,0}$ has slope 1.3971874588115487 with R^2 0.974654118517
9496
$D_{gb} = 0.1D_{gb,0}$ $D_{s} = 0.01D_{s,0}$ has slope 3.2583287352816432 w
ith R^2 0.7528165919811572
$D_{s} = 0.01D_{s,0}$ has slope 0.7789718238763268 with R^2 0.9751591904932
92
$D_{s} = 0.1D_{s,0}$ has slope 0.6317318288039176 with R^2 0.98259503641682
28
```

The surface area decreases linearly with density as expected.

In [28]:

```python
fig, ax = plt.subplots()
#ax2 = ax.twinx()
trtaufac = {}


trsizen = set()
def iplot(fig, ax,  k):
    v = datdict[k]
    taufac = float(taufHandler(k, pprint=None, nodefaultprint=False))
    trtaufac[taufac] = taufHandler(k, nodefaultprint=False)
    ls = lsen[taufac]

    densdf = v["density"]
    gsize = v["avgRadius"]
    poro = v["isolated"]
    try:
        ssa = poro["surfarea"]
    except KeyError as e:
        print(e)
        print(k, "probably not done evaluating yet, skipping")
        return
```

```python
    euler = poro["euler"]

    time = densdf["time"]
    dens = densdf["density"]
    filt = (dens < 1) #& (time <= 3) #& (time*t0*1e3 < 1.2*t0*1e3)
    xdat = dens[filt] #time[filt]
    ydat = ssa[filt] * (l0**2) * (1e6)**2 #dens[filt]
    #/ ssa[filt][0] #

    lab = mkName_GG(k)
    # and "_tauf100_" in k
    if len(lab.replace(" ", "")) == 0: # too many autohandlers in between ma
        lab = "base"
    col = gg_colors[lab]
    mevery=1

    slope, intercept, r_value, p_value, std_err = stats.linregress(xdat, yda

    print(lab, "has R^2", r_value**2)
    def poly(x, slope=slope, intercept=intercept):
        return x*slope + intercept

    ax.plot(xdat, poly(xdat), "--", color=col)

    pl = ax.plot(xdat, ydat, ls.replace("-", ""),
        color=col,
           markevery=mevery,
                 alpha=0.5
                )
           #label=lab)


ik, ilab = [], []
for k in names_GG:
    if "dx0.1" in k and "r12" in k and "pack400" in k and "tauf10_" not in k
    "relaxf" not in k or "relaxf1_" in k) and "_D" in k:
        lab = mkName_GG(k)
        ik.append(k)
        ilab.append(lab)

tmpilab = ilab.copy()
tmpilab.append("base")
ulab = sorted(set(tmpilab), key=natkey)
gg_colors = {k: v for k, v in zip(ulab, coloursen)}
gg_colors["base"] = coloursen[-1]
ik = [x for _, x in sorted(zip(ilab, ik), key=natkey)]

iplot(fig, ax,  basesim)
#iplot(fig, ax,  basesim1)

for lab, col in gg_colors.items():
    ax.plot([], [], "s", color=col, label=lab)


for k in ik:
    iplot(fig, ax,  k)
```

```
for taufac, lab in trtaufac.items():
    ls = lsen[taufac]
    ax.plot([], [], ls.replace("-", ""), color="black", label=lab)



ax.set_xlabel("density / -")
#ax.set_ylabel(r"$A_s$ / $A_{s,0}$ / -")
ax.set_ylabel(r"$A_s$ / $\mathrm{µm}^2$")
ax.legend()
fig.tight_layout()
###fig.savefig("surf-densi-nogg.pdf", bbox_inches="tight")
```

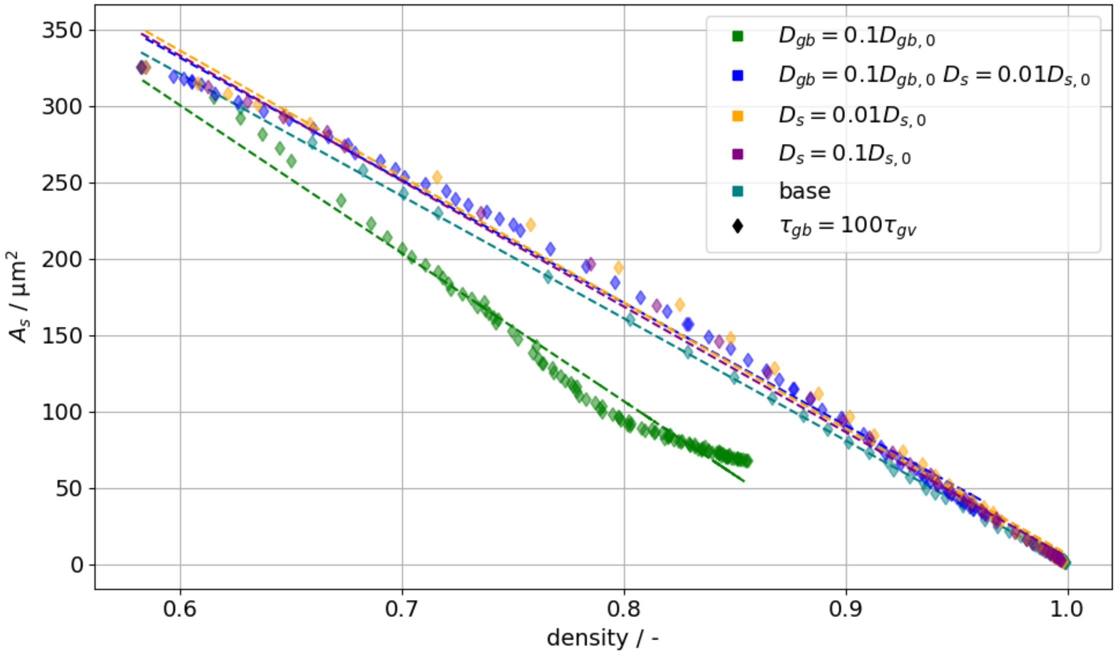


```
base has R^2 0.9996148950457886
$D_{gb} = 0.1D_{gb,0}$ has R^2 0.9758003528943158
$D_{gb} = 0.1D_{gb,0}$ $D_{s} = 0.01D_{s,0}$ has R^2 0.9940655902809522
$D_{s} = 0.01D_{s,0}$ has R^2 0.9924364403272505
$D_{s} = 0.1D_{s,0}$ has R^2 0.9958530473152493
```

In [29]:
```
fig, ax = plt.subplots()
#ax2 = ax.twinx()
trtaufac = {}



trsizen = set()
def iplot(fig, ax,  k):
    v = datdict[k]
    taufac = float(taufHandler(k, pprint=None, nodefaultprint=False))
    trtaufac[taufac] = taufHandler(k, nodefaultprint=False)
    ls = lsen[taufac]

    densdf = v["density"]
    gsize = v["avgRadius"]
```

```
    poro = v["isolated"]
    try:
        ssa = poro["surfarea"]
    except KeyError as e:
        print(e)
        print(k, "probably not done evaluating yet, skipping")
        return
    euler = poro["euler"]

    time = densdf["time"]
    dens = densdf["density"]
    filt = (dens < 1) #& (time <= 3) #& (time*t0*1e3 < 1.2*t0*1e3)
    xdat = dens[filt] #time[filt]
    ydat = ssa[filt] * (l0**2) * (1e6)**2#dens[filt]


    lab = mkName_GG(k)
    # and "_tauf100_" in k
    if len(lab.replace(" ", "")) == 0: # too many autohandlers in between ma
        lab = "base"
    col = gg_colors[lab]
    mevery=1
    slope, intercept, r_value, p_value, std_err = stats.linregress(xdat, yda
    #print(r_value**2, p_value)
    print(lab, "has R^2", r_value**2)
    def poly(x, slope=slope, intercept=intercept):
        return x*slope + intercept

    ax.plot(xdat, poly(xdat), "--", color=col)

    pl = ax.plot(xdat, ydat, ls.replace("-", ""),
        color=col,
           markevery=mevery,
                 alpha=0.5
                )
           #label=lab)


ik, ilab = [], []
for k in names_GG:
    if "dx0.1" in k and "r12" in k and "pack400" in k and "tauf10_" not in k
    "relaxf" not in k or "relaxf1_" in k) and "_D" in k:
        lab = mkName_GG(k)
        ik.append(k)
        ilab.append(lab)

tmpilab = ilab.copy()
tmpilab.append("base")
ulab = sorted(set(tmpilab), key=natkey)
gg_colors = {k: v for k, v in zip(ulab, coloursen)}
gg_colors["base"] = coloursen[-1]
ik = [x for _, x in sorted(zip(ilab, ik), key=natkey)]

#iplot(fig, ax,  basesim)
iplot(fig, ax,  basesim1)
```

```
for lab, col in gg_colors.items():
    ax.plot([], [], "s", color=col, label=lab)


for k in ik:
    iplot(fig, ax,  k)

for taufac, lab in trtaufac.items():
    ls = lsen[taufac]
    ax.plot([], [], ls.replace("-", ""), color="black", label=lab)



ax.set_xlabel("density / -")
ax.set_ylabel(r"$A_s$ / $\mathrm{µm}^2$")
ax.legend()
fig.tight_layout()
##fig.savefig("surf-densi-gg.pdf", bbox_inches="tight")
```

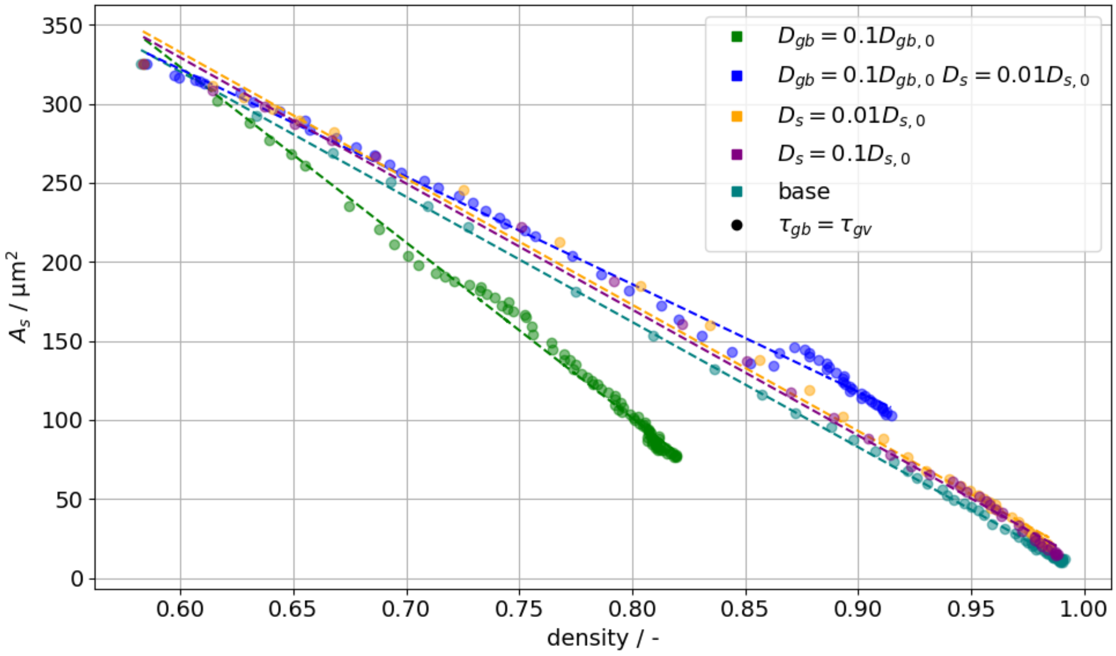


```
base has R^2 0.9993964597180112
$D_{gb} = 0.1D_{gb,0}$ has R^2 0.9918098798928191
$D_{gb} = 0.1D_{gb,0}$ $D_{s} = 0.01D_{s,0}$ has R^2 0.994944458551804
$D_{s} = 0.01D_{s,0}$ has R^2 0.9951427497916387
$D_{s} = 0.1D_{s,0}$ has R^2 0.9966996404935186
```

Next the Euler characteristic as well as the differentiation of open/closed porosity are shown. The open porosity at later stages is likely overestimated due to how density is calculated, since surface roughness reduces the measured density.

The measurement of isolated porosity is exact since it is based on the exact measurement of total grain volume (`gsum`) and isolated pore volume (`isum`). The isolated pore volume is easily calculated after identifying the isolated pore space via connected components.

The two-part plot is to ensure alignment of the x-axis; execute both cells below.

In [30]:

```
#fsize
fig, axx = plt.subplots(2,1, figsize=(fsize[0]+3,fsize[1]*2))
ax = axx[0]
#ax2 = ax.twinx()
trtaufac = {}
maxps = {}
trsizen = set()
def iplot(fig, ax,   k):
    #print(k)
    v = datdict[k]
    taufac = float(taufHandler(k, pprint=None, nodefaultprint=False))
    trtaufac[taufac] = taufHandler(k, nodefaultprint=False)
    ls = lsen[taufac]

    densdf = v["density"]
    gsize = v["avgRadius"]
    poro = v["isolated"]
    try:
        ssa = poro["surfarea"]
    except KeyError as e:
        print(e)
        print(k, "probably not done evaluating yet, skipping")
        return
    euler = poro["euler"]
    isum = datdict[k]["density"]["isum"] # sum(isolated) via CCM of phiv > 0

    poro = 1-densdf["density"]
    iporo = 1-densdf["gsum"] / (densdf["gsum"] + densdf["isum"])
    oporo = poro-iporo



    time = densdf["time"]
    dens = densdf["density"]
    filt = (dens < 1) #& (time <= 3) #& (time*t0*1e3 < 1.2*t0*1e3)
    xdat = (dens)[filt] #dens[filt] #time[filt]
    #xdat = time[filt]
    ydat = euler[filt] #dens[filt]
    maxp = np.argmax(ydat)
    maxps[k] = dens[maxp]

    lab = mkName_GG(k)
    # and "_tauf100_" in k
    if len(lab.replace(" ", "")) == 0: # too many autohandlers in between ma
        lab = "base"
    col = gg_colors[lab]
    mevery=1

    pl = ax.plot(xdat, ydat, ls,
        color=col, alpha=0.5,
           markevery=1,
                )
           #label=lab)
```

```
ik, ilab = [], []
for k in names_GG:
    if "dx0.1" in k and "r12" in k and "pack400" in k and "tauf10_" not in k
    "relaxf" not in k or "relaxf1_" in k) and "_D" in k:
        lab = mkName_GG(k)
        ik.append(k)
        ilab.append(lab)

tmpilab = ilab.copy()
tmpilab.append("base")
ulab = sorted(set(tmpilab), key=natkey)
gg_colors = {k: v for k, v in zip(ulab, coloursen)}
gg_colors["base"] = coloursen[-1]
ik = [x for _, x in sorted(zip(ilab, ik), key=natkey)]

iplot(fig, ax,  basesim)
iplot(fig, ax,  basesim1)

for lab, col in gg_colors.items():
    ax.plot([], [], "s", color=col, label=lab)


for k in ik:
    iplot(fig, ax,  k)

for taufac, lab in trtaufac.items():
    ls = lsen[taufac]
    ax.plot([], [], ls.replace("-", ""), color="black", label=lab)


ax.set_xlim(0.57,1.01)
#ax.set_xlabel("density / -")
ax.set_ylabel(r"Euler char. / -")
ax.legend(ncol=3, loc="lower center", bbox_to_anchor=(0.5, 1))
fig.tight_layout()
###fig.savefig("euler-density.pdf", bbox_inches="tight")
```

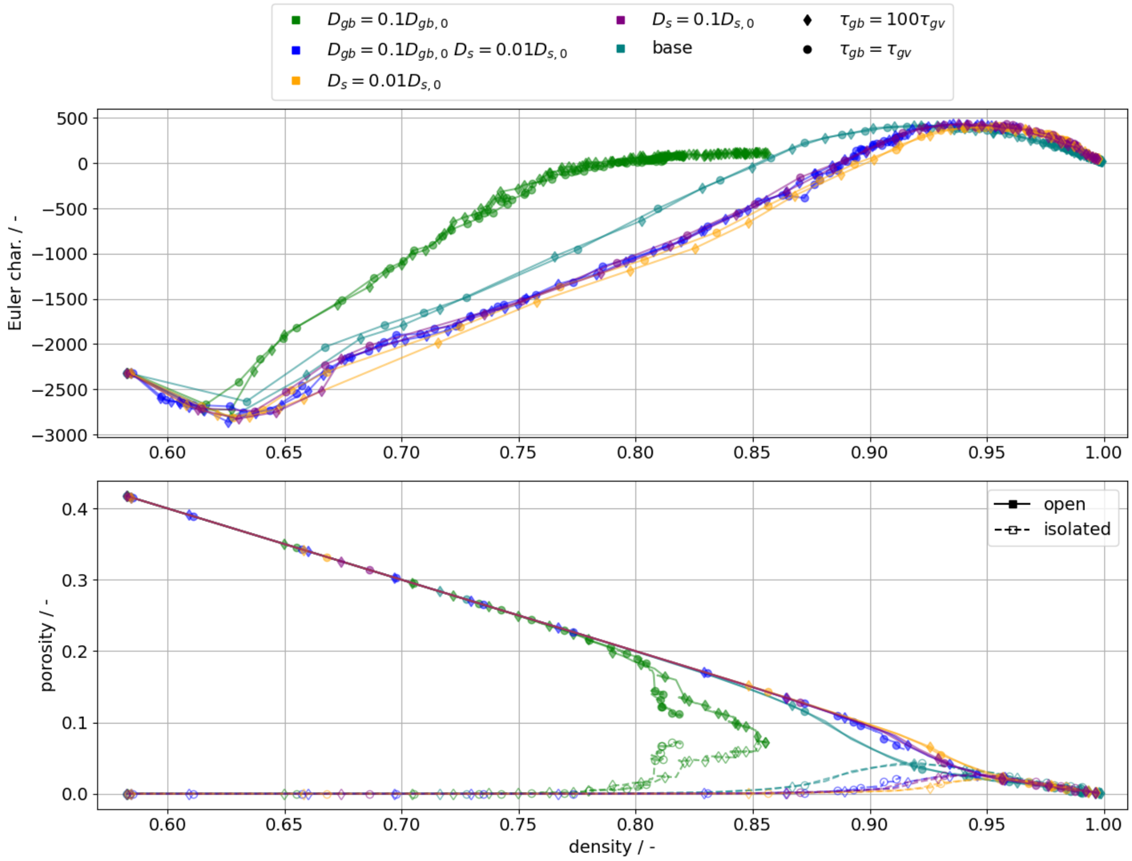

In [31]:
```
#fig, ax = plt.subplots()
ax = axx[1]

#ax2 = ax.twinx()
trtaufac = {}


trsizen = set()
def iplot(fig, ax,  k):
    v = datdict[k]
    taufac = float(taufHandler(k, pprint=None, nodefaultprint=False))
    trtaufac[taufac] = taufHandler(k, nodefaultprint=False)
    ls = lsen[taufac]

    densdf = v["density"]
    gsize = v["avgRadius"]
    poro = v["isolated"]
    try:
        ssa = poro["surfarea"]
    except KeyError as e:
        print(e)
        print(k, "probably not done evaluating yet, skipping")
        return
    euler = poro["euler"]
    isum = datdict[k]["density"]["isum"] # sum(isolated) via CCM of phiv > 0

    poro = 1-densdf["density"]
    iporo = 1-densdf["gsum"] / (densdf["gsum"] + densdf["isum"])
```

```
    oporo = poro-iporo



    time = densdf["time"]
    dens = densdf["density"]
    filt = (dens < 1) #& (time <= 3) #& (time*t0*1e3 < 1.2*t0*1e3)
    xdat = (dens)[filt] #dens[filt] #time[filt]
    #xdat = time[filt]
    ydat = euler[filt] #dens[filt]

    lab = mkName_GG(k)
    # and "_tauf100_" in k
    if len(lab.replace(" ", "")) == 0: # too many autohandlers in between ma
        lab = "base"
    col = gg_colors[lab]
    mevery=2

    ax.plot(xdat, (iporo)[filt], ls.replace("-", "--"), color=col, alpha=0.5
    ax.plot(xdat, (oporo)[filt], ls, color=col, alpha=0.5, markevery=5 )


ik, ilab = [], []
for k in names_GG:
    if "dx0.1" in k and "r12" in k and "pack400" in k and "tauf10_" not in k
    "relaxf" not in k or "relaxf1_" in k) and "_D" in k:
        lab = mkName_GG(k)
        ik.append(k)
        ilab.append(lab)

tmpilab = ilab.copy()
tmpilab.append("base")
ulab = sorted(set(tmpilab), key=natkey)
gg_colors = {k: v for k, v in zip(ulab, coloursen)}
gg_colors["base"] = coloursen[-1]
ik = [x for _, x in sorted(zip(ilab, ik), key=natkey)]

iplot(fig, ax,  basesim)
iplot(fig, ax,  basesim1)


for k in ik:
    iplot(fig, ax, k)




ax.plot([], [], "-s", color="black", label="open")
ax.plot([], [], "--s", color="black", label="isolated", fillstyle="none")

ax.set_xlim(0.57,1.01)
ax.set_xlabel("density / -")
ax.set_ylabel(r"porosity / -")

ax.legend()
fig.tight_layout()
##fig.savefig("euler-density-porosity.pdf", bbox_inches="tight")
```

Following are mainly plots concerning the invariants. When investigating a single invariant, which one can be changed via `Omidx`, with the object type being changeable via `domaintype`. `g` is for grains, `i` for isolated porosity and `d` for detached porosity.

The standard deviation can be visualized with an errorbar by adding `yerr=std_y` to the ax.errorbar call. Though as mentioned in the paper, the large size of the standard deviation overshadows the average evolution.

Time overview of the evolution of the third invariant Ω_3, limited to early times since the long time evolution of reduced grain boundary diffusion makes the behaviour of the other simulations somewhat hard to discern. Comment out the ax.set_xlim call to show the entire time range.

In [32]:
```
fig, ax = plt.subplots()
#ax2 = ax.twinx()
trtaufac = {}

Omidx = 2
trsizen = set()
domaintype = "i"
def iplot(fig, ax,  k):
    v = datdict[k]
    taufac = float(taufHandler(k, pprint=None, nodefaultprint=False))
    trtaufac[taufac] = taufHandler(k, nodefaultprint=False)
    ls = lsen[taufac]
    try:
        invdict = v["invariants"][domaintype]
    except KeyError as e:
        print(e)
        print(k, "probably not finished yet, skipping")
        return
    # because [:-1] derp for now, did phasecount instead of phasecount-1
    if domaintype == "g":
        moments = invdict["mom"][:,:-1,:,:]
        sizen = invdict["vol"][:,:-1]
    else:
        moments = invdict["mom"]
        sizen = invdict["vol"]

    densdf = v["density"]
    neighs = (v["neighss"])
    # maybe i should go back to masked arrays ...


    lab = mkName_GG(k)
    # and "_tauf100_" in k
    if len(lab.replace(" ", "")) == 0: # too many autohandlers in between ma
        lab = "base"
    col = gg_colors[lab]
    xdat = densdf["time"] * t0 * 1e3
    avg_y = []
    std_y = []
    for i in range(sizen.shape[0]):
```

```
        nonzerofilt = (sizen[i] > 5**3)
        # objects only consisting of interface generally don't approximate t
        # hence a size filter is employed here
        nonzerocount = np.count_nonzero(nonzerofilt)
        if (nonzerocount < 5):
            avg_y.append(np.nan)
            std_y.append(np.nan)
            continue
        invs, volfilt = filteredInvariantCalc(moments[i].astype(np.float64),
                                             sizen[i].astype(np.float64),
                                             nonzerofilt)

        for j in range(3):
            invs[j] /= Oms_ref["sphere"][j]

        #print(invs[Omidx].shape, invs[Omidx].dtype)
        filt2 = np.isfinite(invs[Omidx])

        avg_y.append(np.mean(invs[Omidx][filt2]))
        #print(invs.shape, invs[2])
        std_y.append(np.std(invs[Omidx][filt2]))
        #invlist.append(invs)
    #ax.plot(xdat, avg_y, ls,
    #       color=col,alpha=0.5, )
    #print(j, avg_y, std_y)
    if np.all(np.isnan(std_y)):
        return
    #yerr=std_y,
    ax.errorbar(xdat, avg_y, fmt=ls,
    color=col,alpha=0.5, capsize=3,
               )



ik, ilab = [], []
for k in names_GG:
    #and "tauf100_" not in k
    #and "Dsf0.1" not in k
    if "dx0.1" in k and "r12" in k and "pack400" in k and (
    "relaxf" not in k or "relaxf1_" in k) and "_D" in k:
        lab = mkName_GG(k)
        ik.append(k)
        ilab.append(lab)

tmpilab = ilab.copy()
tmpilab.append("base")
ulab = sorted(set(tmpilab), key=natkey)
gg_colors = {k: v for k, v in zip(ulab, coloursen)}
gg_colors["base"] = coloursen[-1]
ik = [x for _, x in sorted(zip(ilab, ik), key=natkey)]

iplot(fig, ax,  basesim)
iplot(fig, ax,  basesim1)

for lab, col in gg_colors.items():
    ax.plot([], [], "s", color=col, label=lab)
```

```
# not necessary but copypasta hooray
#ilab = [x for x, _ in sorted(zip(ilab, ik), key=natkey)]
for k in ik:
    iplot(fig, ax,  k)

for taufac, lab in trtaufac.items():
    ls = lsen[taufac]
    ax.plot([], [], ls.replace("-", ""), color="black", label=lab)


#ax.set_xlabel(r"$1/sqrt(1-rho)$ / -")
ax.set_xlabel(r"time / ms" )
ax.set_ylabel(r"$\Omega_%d$ / -" % (Omidx+1))
#ax.set_xlim(-0.1, 20)
if domaintype == "g":
    ax.axhline(Oms_ref["trunc_octa"][Omidx]/Oms_ref["sphere"][Omidx], color=
#ax.set_ylim(0.1, 1.1)
#ax.legend(ncol=1, loc='center left', bbox_to_anchor=(1.15, 0.5))
#ax.legend(ncol=3, loc='upper center', bbox_to_anchor=(0.5, 1.25))
ax.set_xlim(-0.1, 2) # remove this if you want to see the entire time line
#ax.set_xscale("log")
ax.legend(ncol=2, title=domainnames[domaintype])
#, title=fullnames[domaintype]
fig.tight_layout()
#ax2.grid(False)
###fig.savefig("om%d_%s.pdf" % (Omidx+1, fullnames[domaintype]), bbox_inches
```

```
/tmp/ipykernel_2578220/1284260526.py:1: RuntimeWarning: More than 20 figure
s have been opened. Figures created through the pyplot interface (`matplotl
ib.pyplot.figure`) are retained until explicitly closed and may consume too
much memory. (To control this warning, see the rcParam `figure.max_open_war
ning`). Consider using `matplotlib.pyplot.close()`.
  fig, ax = plt.subplots()
```

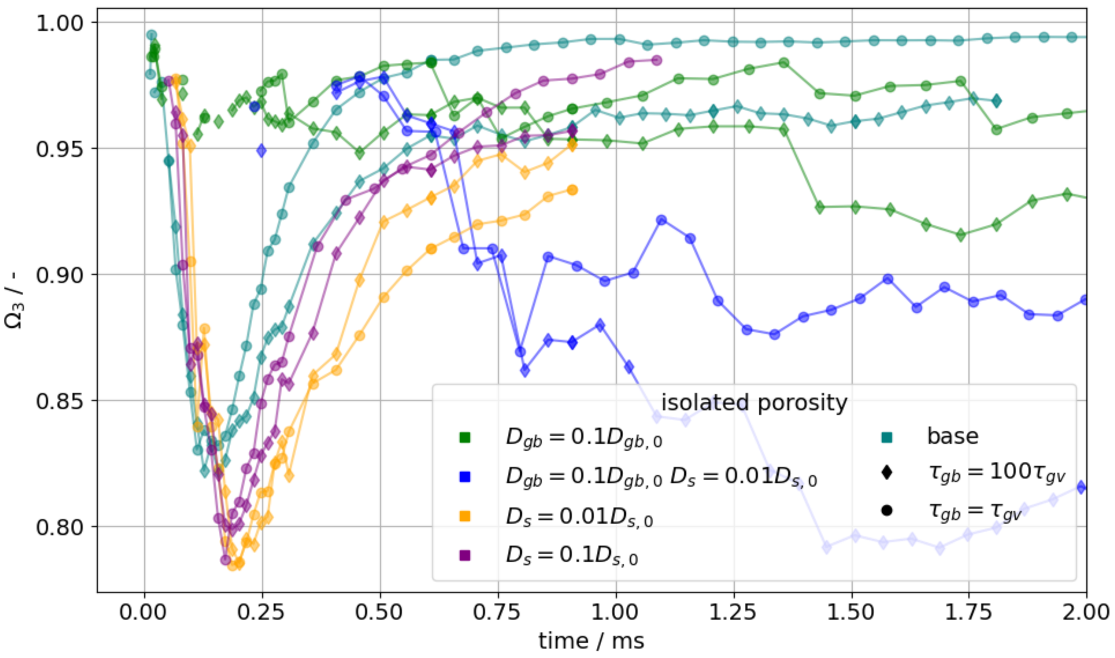

Set `loop=True` below to generate PDFs of all combinations automatically.

In [33]:
```
# loop for generating all combinations of Omidx and domaintype
Oms = [0, 1, 2]
domains = list(domainnames.keys())
loop = False # for generating PDFs of all combinations
for Omidx2 in Oms:
    for domain in domains:
        if loop:
            Omidx = Omidx2
            domaintype = domain
        else:
            # change Omidx to which invariant to plot
            Omidx = 2
            # and domaintype to your desired structure
            domaintype = "i"

        fig, ax = plt.subplots()
        #if domaintype != "g":
        #    fig, ax = plt.subplots(figsize=(6,5))
        #else:
        #    fig, ax = plt.subplots(figsize=(12, 5))
        #ax2 = ax.twinx()
        trtaufac = {}

        trsizen = set()

        nanarr = np.nan * np.ones(3)
        def iplot(fig, ax,  k):
            v = datdict[k]
            taufac = float(taufHandler(k, pprint=None, nodefaultprint=False)
            trtaufac[taufac] = taufHandler(k, nodefaultprint=False)
            ls = lsen[taufac]
            try:
                invdict = v["invariants"][domaintype]
            except KeyError as e:
                print(e)
                print(k, "probably not finished yet, skipping")
                return
            # because [:-1] derp for now, did phasecount instead of phasecou
            if domaintype == "g":
                moments = invdict["mom"][:,:-1,:,:]
                sizen = invdict["vol"][:,:-1]
            else:
                moments = invdict["mom"]
                sizen = invdict["vol"]

            densdf = v["density"]
            neighs = (v["neighss"])
            # maybe i should go back to masked arrays ...


            lab = mkName_GG(k)
            # and "_tauf100_" in k
            if len(lab.replace(" ", "")) == 0: # too many autohandlers in be
```

```
            lab = "base"
        col = gg_colors[lab]
        #xdat = densdf["time"] #* t0 * 1e3
        xdat = densdf["density"]
        avg_y = []
        std_y = []
        for i in range(sizen.shape[0]):
            nonzerofilt = (sizen[i] > 5**3)

            nonzerocount = np.count_nonzero(nonzerofilt)
            if (nonzerocount < 5):
                avg_y.append(np.nan)
                std_y.append(np.nan)
                continue
            invs, volfilt = filteredInvariantCalc(moments[i].astype(np.f
                                                  sizen[i].astype(np.flo
                                                  nonzerofilt)

            for j in range(3):
                invs[j] /= Oms_ref["sphere"][j]

            #print(invs[Omidx].shape, invs[Omidx].dtype)
            filt2 = np.isfinite(invs[Omidx])

            avg_y.append(np.mean(invs[Omidx][filt2]))
            #print(invs.shape, invs[2])
            std_y.append(np.std(invs[Omidx][filt2]))
            #invlist.append(invs)
        #ax.plot(xdat, avg_y, ls,
        #       color=col,alpha=0.5, )
        #print(j, avg_y, std_y)
        if np.all(np.isnan(std_y)):
            return
        #yerr=std_y,
        ax.errorbar(xdat, avg_y, fmt=ls.replace("-", ""),
        color=col,alpha=0.5, capsize=3,
              )

    ik, ilab = [], []
    for k in names_GG:
        #and "tauf100_" not in k
        #and "Dsf0.1" not in k
        if "dx0.1" in k and "r12" in k and "pack400" in k and (
        "relaxf" not in k or "relaxf1_" in k) and "_D" in k:
            lab = mkName_GG(k)
            ik.append(k)
            ilab.append(lab)

    tmpilab = ilab.copy()
    tmpilab.append("base")
    ulab = sorted(set(tmpilab), key=natkey)
    gg_colors = {k: v for k, v in zip(ulab, coloursen)}
    gg_colors["base"] = coloursen[-1]
    ik = [x for _, x in sorted(zip(ilab, ik), key=natkey)]

    iplot(fig, ax,  basesim)
```

```
        iplot(fig, ax,  basesim1)

        for lab, col in gg_colors.items():
            ax.plot([], [], "s", color=col, label=lab)

        for k in ik:
            iplot(fig, ax,  k)

        for taufac, lab in trtaufac.items():
            ls = lsen[taufac]
            ax.plot([], [], ls.replace("-", ""), color="black", label=lab)

        ax.set_xlabel(r"density / -" )
        ax.set_ylabel(r"$\Omega_%d$ / -" % (Omidx+1))
        if domaintype == "g":
            truncp = 0.5 * ( (Oms_ref["trunc_octa"][Omidx]) + Oms_ref["cube"
            diff = truncp - Oms_ref["cube"][Omidx] / Oms_ref["sphere"][Omidx
            ax.axhline(Oms_ref["trunc_octa"][Omidx]/Oms_ref["sphere"][Omidx]
            ax.axhline(Oms_ref["cube"][Omidx]/Oms_ref["sphere"][Omidx], ls="
            #ax.annotate
            tpos = (0.625, truncp )
                    #Oms_ref["trunc_octa"][Omidx]/Oms_ref["sphere"][Omidx]-0
            ax.annotate("trunc. octahedron", xytext=tpos,
                        xy=(tpos[0], Oms_ref["trunc_octa"][Omidx]/Oms_ref["s
                        arrowprops=dict(arrowstyle='->, head_width=0.1', col
                        ha='center'
                       )
            tpos = (0.625, Oms_ref["cube"][Omidx]/Oms_ref["sphere"][Omidx]-0
            ax.annotate("cube", xytext=tpos,
                        xy=(tpos[0], Oms_ref["cube"][Omidx]/Oms_ref["sphere"
                        arrowprops=dict(arrowstyle='->, head_width=0.1', col
                        ha='center'
                       )
        elif domaintype != "g" and Omidx == 2:
            ax.set_ylim(0.7, 1.15)
        ax.legend(ncol=2, title=domainnames[domaintype])
        fig.tight_layout()
        #ax2.grid(False)
        if loop:
            #fig.savefig("om%d_%s.pdf" % (Omidx+1, domainnames[domaintype]),
        if not loop:
            break
        else:
            plt.close()
            del fig, ax
    if not loop:
        break
# disgusting but it works
```

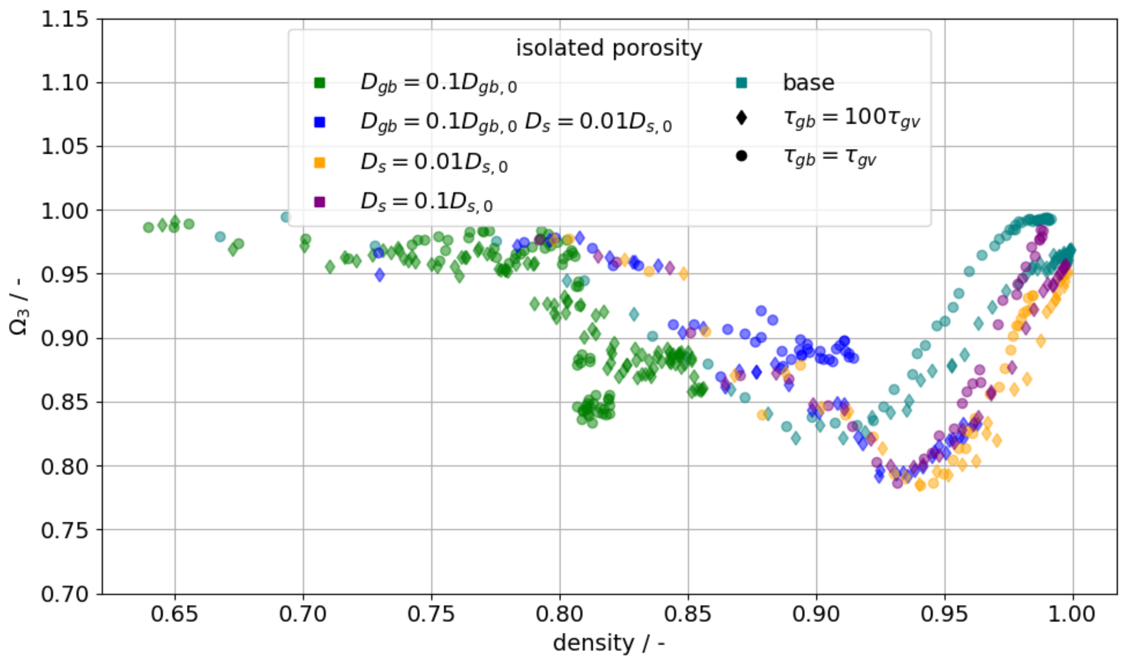

The lumped invariant is shown below as well.

In [34]:
```
Oms = [0, 1, 2]
domains = list(domainnames.keys())
mpl.rcParams.update({'font.size': 14})

domaintype = "g"
fig, ax = plt.subplots(figsize=(10, 6))
#if domaintype != "g":
#    fig, ax = plt.subplots(figsize=(6,5))
#else:
#    fig, ax = plt.subplots(figsize=(12, 5))
#ax2 = ax.twinx()
trtaufac = {}

#Omidx = 2
trsizen = set()

nanarr = np.nan * np.ones(3)
def iplot(fig, ax,  k):
    v = datdict[k]
    taufac = float(taufHandler(k, pprint=None, nodefaultprint=False))
    trtaufac[taufac] = taufHandler(k, nodefaultprint=False)
    ls = lsen[taufac]
    try:
        invdict = v["invariants"][domaintype]
    except KeyError as e:
        print(e)
        print(k, "probably not finished yet, skipping")
        return
    # because [:-1] derp for now, did phasecount instead of phasecount-2
    #if domaintype == "g":
    #    moments = invdict["mom"][:,:-1,:,:]
    #    sizen = invdict["vol"][:,:-1]
```

```
    #else:
    #    moments = invdict["mom"]
    #    sizen = invdict["vol"]

    moments = invdict["mom"][:,:-1,:,:]
    sizen = invdict["vol"][:,:-1]

    densdf = v["density"]
    neighs = (v["neighss"])
    # maybe i should go back to masked arrays ...


    lab = mkName_GG(k)
    # and "_tauf100_" in k
    if len(lab.replace(" ", "")) == 0: # too many autohandlers in between ma
        lab = "base"
    col = gg_colors[lab]
    xdat = densdf["time"] * t0 * 1e3
    xdat = densdf["density"]
    avg_y = []
    std_y = []
    for i in range(sizen.shape[0]):
        nonzerofilt = (sizen[i] > 5**3)
        nonzerocount = np.count_nonzero(nonzerofilt)
        if (nonzerocount < 5):
            avg_y.append(np.nan)
            std_y.append(np.nan)
            continue
        invs, volfilt = filteredInvariantCalc(moments[i].astype(np.float64),
                                              sizen[i].astype(np.float64),
                                              nonzerofilt)

        for j in range(3):
            invs[j] /= Oms_ref["sphere"][j]

        #print(invs[Omidx].shape, invs[Omidx].dtype)
        filt2 = np.all(np.isfinite(invs), axis=0)
        invvar_mean = np.linalg.norm(invs[:,filt2], ord=2, axis=0)
        invvar_stds = np.linalg.norm(invs[:,filt2], ord=2, axis=0)

        invmeans = np.mean(invvar_mean)
        invstd =   np.std(invvar_mean)

        #invmeans = np.mean(invs[:,filt2], axis=1)
        #invstd =   np.std(invs[:,filt2], axis=1)

        #invvar_mean = np.linalg.norm(invmeans, ord=2)
        #invvar_stds = np.linalg.norm(invstd, ord=2)

        avg_y.append(invmeans)
        std_y.append(invstd)
        #print(invs.shape, invs[2])

        #invlist.append(invs)
    #ax.plot(xdat, avg_y, ls,
    #print(invs.shape)
```

```
    #       color=col,alpha=0.5, )
    #print(j, avg_y, std_y)
    if np.all(np.isnan(std_y)):
        return
    #yerr=std_y,
    ax.errorbar(xdat, avg_y/np.sqrt(3), fmt=ls.replace("-", ""),
    color=col,alpha=0.5, capsize=3,
          )
    return invs

ik, ilab = [], []
for k in names_GG:
    #and "tauf100_" not in k
    #and "Dsf0.1" not in k
    if "dx0.1" in k and "r12" in k and "pack400" in k and (
    "relaxf" not in k or "relaxf1_" in k) and "_D" in k:
        lab = mkName_GG(k)
        ik.append(k)
        ilab.append(lab)

tmpilab = ilab.copy()
tmpilab.append("base")
ulab = sorted(set(tmpilab), key=natkey)
gg_colors = {k: v for k, v in zip(ulab, coloursen)}
gg_colors["base"] = coloursen[-1]
ik = [x for _, x in sorted(zip(ilab, ik), key=natkey)]

iplot(fig, ax,  basesim)
iplot(fig, ax,  basesim1)

for lab, col in gg_colors.items():
    ax.plot([], [], "s", color=col, label=lab)

# not necessary but copypasta hooray
#ilab = [x for x, _ in sorted(zip(ilab, ik), key=natkey)]
for k in ik:
    iplot(fig, ax,  k)

for taufac, lab in trtaufac.items():
    ls = lsen[taufac]
    ax.plot([], [], ls.replace("-", ""), color="black", label=lab)
#ax2.plot([], [], "-", color="black", label="density", fillstyle="none")
#ax2.plot([], [], "--", color="black", label="grain size", fillstyle="none")

#ax2.set_ylabel("grain size / nm")
#ax2.legend(loc="upper right", ncol=2)


#ax.set_xlabel(r"$1/sqrt(1-rho)$ / -")
ax.set_xlabel(r"density / -" )
#ax.set_ylabel(r"$\frac{||\Omega||}{\sqrt{3}}$ / -")
ax.set_ylabel(r"$\Omega_E$ / -")
#ax.set_xlim(-0.1, 20)
truncval = np.linalg.norm(Oms_ref["trunc_octa"] / Oms_ref["sphere"], ord=2)
cubval = np.linalg.norm(Oms_ref["cube"] / Oms_ref["sphere"], ord=2) /np.sqrt
if domaintype == "g":
```

```
    truncp = 0.5 * (truncval + cubval) #0.5 * ( (Oms_ref["trunc_octa"][Omidx
    diff = truncp - cubval # / Oms_ref["sphere"][Omidx]
    ax.axhline(truncval, color="black")
    ax.axhline(cubval, ls="--", color="black")

    tpos = (0.625, truncp )
            #Oms_ref["trunc_octa"][Omidx]/Oms_ref["sphere"][Omidx]-0.06)
    ax.annotate("trunc. octahedron", xytext=tpos,
                xy=(tpos[0], truncval),
                arrowprops=dict(arrowstyle='->, head_width=0.1', color='blac
                ha='center'
               )
    tpos = (0.625, cubval-diff)
    ax.annotate("cube", xytext=tpos,
                xy=(tpos[0], cubval),
                arrowprops=dict(arrowstyle='->, head_width=0.1', color='blac
                ha='center'
               )
elif domaintype != "g" and Omidx == 2:
    ax.set_ylim(0.7, 1.025)
#ax.set_ylim(0.1, 1.1)
#ax.legend(ncol=1, loc='center left', bbox_to_anchor=(1.15, 0.5))
#ax.legend(ncol=3, loc='upper center', bbox_to_anchor=(0.5, 1.25))
ax.set_xlim(0.57, 1.01)
#ax.set_ylim(0.6, 1.025)
#ax.set_xscale("log")
ax.legend(ncol=2, title=domainnames[domaintype])
fig.tight_layout()
#ax2.grid(False)
##fig.savefig("om_lumped_%s.pdf" % (fullnames[domaintype]), bbox_inches="tig
```

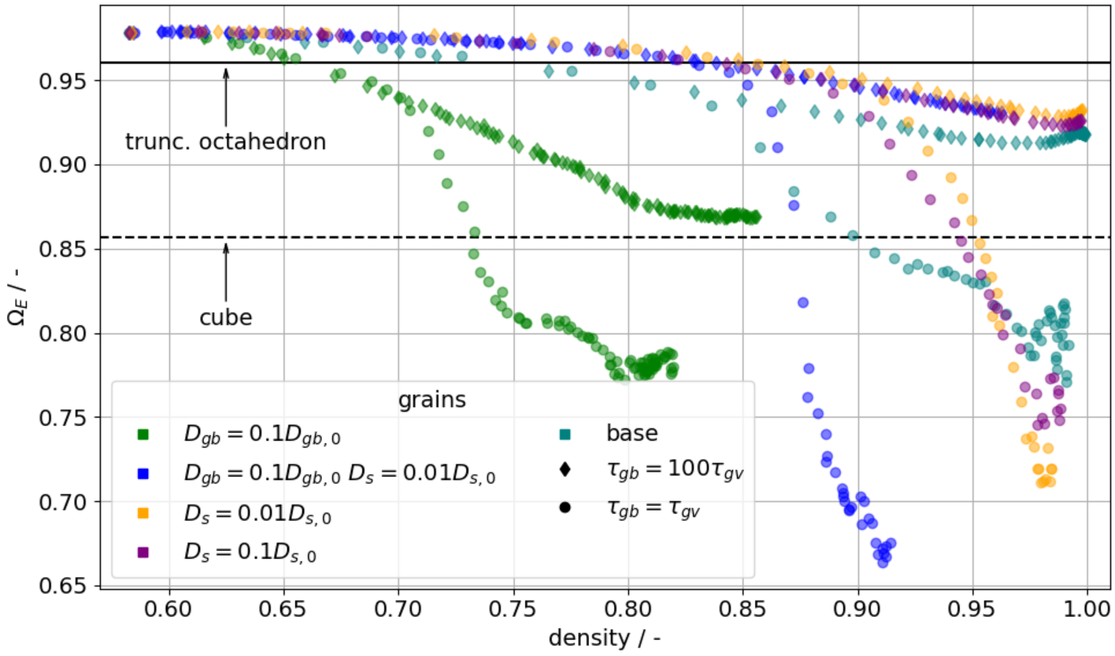

In [ ]:
