# Supplementary material for: Revealing process and material parameter effects on densification via phase-field studies
Source: Sci Rep. 2024 Mar 4;14:5350. doi: 10.1038/s41598-024-51915-w (PMC10912692; doi:10.1038/s41598-024-51915-w)
Supplement: Supplementary file 1 — Supplementary Information. [file 41598_2024_51915_MOESM1_ESM.zip › supmat/scalinglaw.pdf]

In this notebook Herring's scaling law is applied to sintering simulations of a green body approximating a cube with different domain sizes and grain sizes. The goal is to show that the scaling law applies and that thus the results are extendable to larger grain sizes, given the assumptions for Herring's scaling laws are still valid.

The scaling law reads

$$\frac{\Delta t_2}{\Delta t_1} = (\frac{R_2}{R_1})^n \tag{1}$$

with the time to reach a certain state Δt_i for different constant grain sizes R_i. Based on scaling considerations and the dominant diffusion path, $n \in [3, 4]$ usually.

We define the state to be reached as a certain density ρ, with Δt then being measured relative to the simulation start. Thus we would like to have a function $t(\rho)$ which tells us the time to reach the target density ρ. Since the functional form is a priori unknown, $t(\rho)$ will be interpolated based on data available. Note that this only holds if the grain size G is constant during this state transition, which will be verified as well.

Once $t(\rho)$ is available, one can write

$$t_2(\rho) = t_1(\rho)(\frac{R_2}{R_1})^n \tag{2}$$

and with (t_2, t_1, R_1, R_2) known for a range of densities, one can find n as the best-fit which minimizes the error in eq. 2.

We will use all three of the largest simulations ($(800\mathrm{nm})^3$) as reference simulations and take the arithmetic average of the results for each of these to determine n as a whole.

In [1]:
```
%matplotlib notebook
# notebook backend doesn't work on jupyterlab and widgets seems to die
# exclusively on binder, but works fine locally
# hence if you want interactivity in the plots:
# restart kernel, change the inline of the above to notebook and
# go to the notebook view via changing the url from
# https://hub.ovh2.mybinder.org/whatever/lab?
# to
# https://hub.ovh2.mybinder.org/whatever/tree/
# NB that the domain can be different depending on whereever you land.
# the important part is replacing lab with tree to get the notebook view
import matplotlib as mpl
import matplotlib.pyplot as plt
import os
import sys
import matplotlib.patches as patches
import glob
import pandas as pd
from scipy      import optimize, interpolate, stats
# which function is used for fitting is somewhat inconsistent
```

```
import numpy as np
import natsort
import warnings
from pathlib import Path
from helper import *
import re
import matplotlib.patches as patches
from matplotlib.offsetbox import OffsetImage, AnnotationBbox
#mpl.rcParams.update({'font.size': 16})
mpl.rcParams.update({'font.size': 14}) # 18 is in creep
fsize = (10,5)
mpl.rcParams['figure.figsize'] = (10,6)
mpl.rcParams.update({"axes.grid" : True})

# volss: [frame, grain volumes], neighss: [frame, neighbours per grain], com
# all in nondim. cell units
bnen = ["volss"]
npydats = {k: "_"+k+".npy" for k in bnen}

# moment data is also in npy format, but requires a bit of special handling

natkey = natsort.natsort_keygen()


tfacs = [1, 10, 100]
tfacs_ls = ["-o", "-s", "-d"]
lsen = {float(k): v for k,v in zip(tfacs, tfacs_ls)}

densi_end = "_density.dat"

def mkName_noGG_nomu(s):
    handlers = [packHandler, rHandler]
    return mkName(s, handlers=handlers)
def mkName_noGG(s):
    handlers = [packHandler, rHandler, muHandler]
    return mkName(s, handlers=handlers)
def mkName_GG(s, handlers=None):
    # allow some flexibility here since we might want to look at only subspa
    #packHandler, rHandler not since only one box size + particle size is co
    if handlers is None:
        handlers = [taugkfHandler, DgbfHandler, DsfHandler, relaxfHandler]
    return mkName(s, handlers=handlers)
mpl.rcParams['axes.prop_cycle'] = mpl.cycler(color=coloursen)
packs = [200, 400, 800]
ppacks = [r"$(%d \mathrm{nm})^3$" % (x) for x in packs]
packs = [r"$%d^3$" % (x) for x in packs]
npacks = {k:v for k, v in zip(packs, ppacks)}
```

In [2]:

```
def readDat(densitypath, datdict, names, readNpy=True):
    p = Path(densitypath)
    name = p.name.replace(densi_end, "")
    names.append(name)

    idict = {}

    df = pd.read_csv(densitypath, sep=" ")
```

```
    # density is with MC+convex hull
    # gsum/isum are direct sums of (1-phiv) (entire domain) and phi_i (only
    # thus gsum / (gsum+isum) gives an accurate estimate of isolated porosit
    # potentially after gsum/(gsum+isum) went through a global minimum only
    # and hence could be used for an accurate estimate of total porosity at
    # however, this will need to be investigated separately
    df["time"] = df["time"] - df["time"][0] # zero to start
    time = df["time"]
    dens = df["density"]
    #diffts = np.diff(time, prepend=time[0])
    drhodt = np.gradient(dens, time)
    # NB: there are some with very low diffts, don't trust them -> turn to r
    df.loc[:,'densirate'] = pd.Series(drhodt, index=df.index)
    #df["densirate"] = drhodt
    #print(df["densirate"].shape)

    idict["density"] = df.copy()

    # no eval on these immediately, just read in
    dxval = float(redx.search(name).groups()[0])
    if readNpy:
        for k, v in npydats.items():
            #npath = str(p.parent)+"/" + p.name.replace("density.dat", v)
            npath = str(p.parent)+"/" + p.name.replace(densi_end, v)
            dat = np.load(npath)
            idict[k] = dat.copy()

        # avgs for data which is often used
        gv = idict["volss"]
        frames, _ = gv.shape
        rs = np.zeros(frames)
        for f in range(frames):
            tmp = gv[f]
            rv = vol2r(tmp)
            # once r < epsilon, pretty much only interface remains and hence
            avg = physSizeNm ( np.average(rv[rv > 4])*dxval )
            #avg = physSizeNm(vol2r((np.average(tmp[tmp != 0])))*dxval) # vo
            rs[f] = avg
        idict["avgRadius"] = rs #.copy()


    idict["meta"] = {}
    idict["meta_num"] = {}
    # nondimensionalized base values
    tauf0 = 0.08
    Dsf0 = 169
    Dgbf0 = 55
    for identifier, handler in zip(["tauf", "Dsf", "Dgbf"],
                                   [taufHandler, DsfHandler, DgbfHandler]):
        idict["meta_num"][identifier] = eval(identifier+"0")*float(handler(r

    idict["meta"]["dx"] = dxval #

    for identifier, handler in glob_handlers.items():
        val = handler(name) # so you don't have to know the formatting...
        idict["meta"][identifier] = val
```

```
    idict["meta"]["Rg"] = float(idict["meta"]["R"].split(" ")[-2]) # hacky a

    datdict[name] = idict.copy()
```

In [ ]:

In [5]:
```
datdict = {}
names = []
# todo update
bpath = "dataVarG/"
pat = "*density.dat"
#pat = "pack200_dx0.1_r12_mu0_weno_md_musurfPositive_mpt_*density_combined.c
#pat = "pack400_dx0.1_r12*_GG_tauf100_relaxf1_Dgbf0.1*density.dat"
paths = natsort.natsorted(glob.glob(bpath+pat))
for p in paths:
    # if you only want to look at density/isolated pores stuff, don't read i
    # that's mainly per-grain information which isn't relevant for those but
    #print(p)
    readDat(p, datdict, names, readNpy=True)
```

First we'll make sure the grain size has not changed appreciably by plotting it over time. As can be seen from the figure below, there is hardly any change in it; there is a slight reduction which is mostly due to the effect described in

https://doi.org/10.1016/j.jcp.2006.11.020 (Pengtao Yue, Chunfeng Zhou, James J. Feng, Spontaneous shrinkage of drops and mass conservation in phase-field simulations, Journal of Computational Physics, Volume 223, Issue 1, 2007, Pages 1-9)

i.e. that the volume of a single phase need not be conserved against a surrounding phase due to capillary action, even if the phases are coupled with a conserved field.

In [6]:
```
fig, ax = plt.subplots()
trpacks = set()
trsizen = set()
maxdensen = []
mindensen = []
for k in names:
    v = datdict[k]
    meta = v["meta"]
    pack = (meta["pack"])
    R = meta["R"]
    trsizen.add(R)
    trpacks.add(pack)
    col = coloursR[R]
    ls = ls_pack[pack]

    densdf = v["density"]
    time = densdf["time"]
    gsize = v["avgRadius"]
    maxdensen.append(np.max(densdf["density"]))
    mindensen.append(np.min(densdf["density"]))
    time = time-time[0] # from the respawn
    filt = time < np.inf
```

```
    xdat = time[filt]
    ydat = gsize[filt]
    #lab = mkName_noGG_nomu(k)


    mevery=2
    ax.plot(xdat*t0 * 1e3, ydat,ls,
                color=col,
           markevery=mevery)

# track the min/max max/min densities to know the space over which we can ir
minmaxdens = np.min(maxdensen)
maxmindens = np.max(mindensen)

trsizen = natsort.natsorted(list(trsizen))
trpacks = natsort.natsorted(list(trpacks))
for size in trsizen:
    ax.plot([], [], coloursR[size], label=size)
for pack in trpacks:
    ax.plot([], [], ls_pack[pack], color="black", label=npacks[pack])

#title="MDM",
ax.legend(ncol=2)
ax.set_xlabel("time / ms")
ax.set_ylabel("avg. grain size / nm")
```

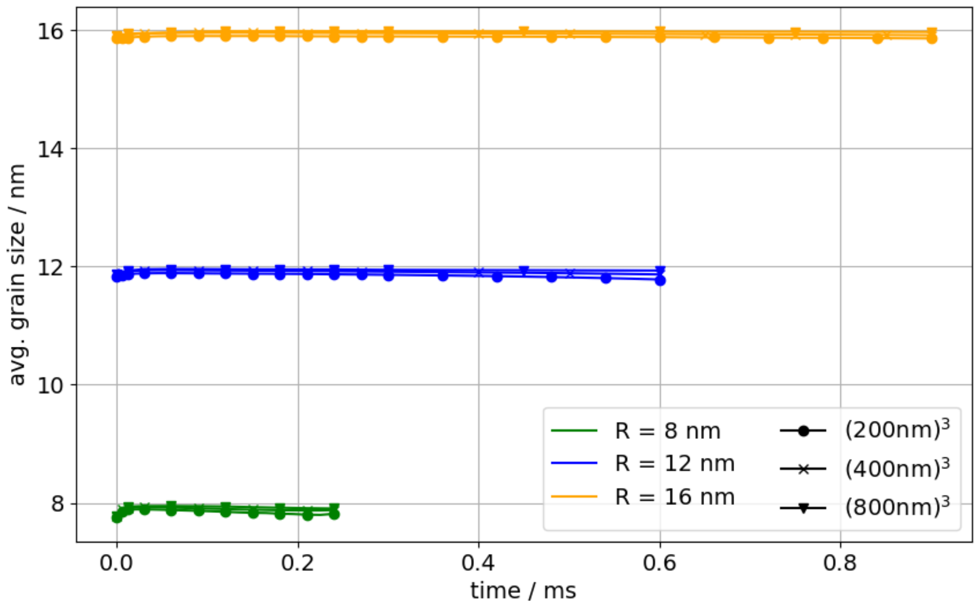

Out[6]: Text(0, 0.5, 'avg. grain size / nm')

In [7]:
```
#refsim = "pack800_dx0.1_r8_mu0_weno_md_musurfPositive_mpt"
#refsim = "pack800_dx0.1_r12_mu0_weno_md_musurfPositive_mpt"
refsims = ["pack800_dx0.1_r%d_mu0_weno_md_musurfPositive_mpt" % x for x in [
```

In [8]:
```
densspace = np.linspace(maxmindens, minmaxdens, 50)
```

In [9]:
```
ns = []
for refsim in refsims:
    # tref, tk = t_2, t_1
    # we take R2, R1 from the initial grain size since it is approximately u
    refdf = datdict[refsim]["density"]
    refts = refdf["time"]
    refdens = refdf["density"]
    Rref = datdict[refsim]["meta"]["Rg"]
    tref = interpolate.CubicSpline(refdens[:],refts[:])
    trefs = tref(densspace)
    for k in names:
        Rk = datdict[k]["meta"]["Rg"]
        if k == refsim or Rk == Rref:
            continue
        kdf = datdict[k]["density"]
        kts = kdf["time"]
        kdens = kdf["density"]
        try:
            tk = interpolate.CubicSpline(kdens[:],kts)
        except ValueError as e:
            continue
        tks = (tk(densspace))

        Rdiv = Rref / Rk
        def herringlog(t, n):
            return t * (Rdiv)**n
        popt, pcov = optimize.curve_fit( herringlog, tks, trefs)
        ns.append(popt[0])
```

In [10]:
```
navg = np.average(ns)
navg
```

Out[10]: 3.270953010318862

Thus

$$t_2(\rho) = t_1(\rho)(\frac{R_1}{R_2})^{3.27} \tag{3}$$

should yield good predictions for the time-to-density of other grain sizes. The easiest way to show this graphically is not to directly apply the equation, but rather to simply rescale time by R^n as is done below to show the excellent overlap for all results, excepting the smallest simulation which is not representative as described in https://doi.org/10.1007/s10853-023-08859-9 .

In [11]:
```
fig, ax = plt.subplots()
trpacks = set()
trsizen = set()
maxdensen = []
mindensen = []
for k in names:
    v = datdict[k]
    meta = v["meta"]
    pack = (meta["pack"])
```

```
    R = meta["R"]
    RR = meta["Rg"]
    trsizen.add(R)
    trpacks.add(pack)
    col = coloursR[R]
    ls = ls_pack[pack]

    densdf = v["density"]
    time = densdf["time"]
    density = densdf["density"]
    time = time
    filt = time < np.inf
    xdat = time[filt]
    ydat = density[filt]
    #lab = mkName_noGG_nomu(k)


    mevery=2
    ax.plot(xdat / RR**navg, ydat,ls,
                color=col,
           markevery=mevery)

trsizen = natsort.natsorted(list(trsizen))
trpacks = natsort.natsorted(list(trpacks))
for size in trsizen:
    ax.plot([], [], coloursR[size], label=size)
for pack in trpacks:
    ax.plot([], [], ls_pack[pack], color="black", label=npacks[pack])

#title="MDM",
ax.legend(ncol=2)
ax.set_xlabel("time / $R^{%.2f}$ / (-)" % (navg))
ax.set_ylabel("density / -")
```

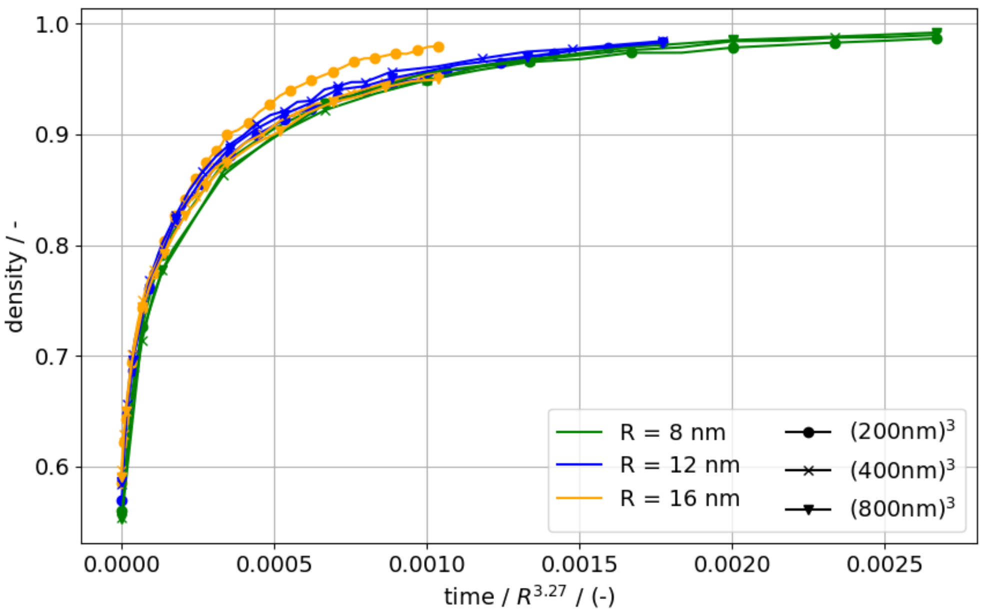

Out[11]: Text(0, 0.5, 'density / -')

In [12]:
```
ddf = datdict["pack800_dx0.1_r8_mu0_weno_md_musurfPositive_mpt"]["density"]
t2rho = ddf["time"][np.argmax(ddf["density"] > 0.99)] * t0 # first time wher
tpred = t2rho * (1/0.008)**(3.27) # scaling law, result in s
tpred
```

Out[12]: 1726.263017103989

We can use the obtained scaling law to see whether we can reach a reasonable approximation of experimental results --- albeit with different process parameters. In https://doi.org/10.1016/j.matlet.2008.05.036 a copper sample with 1µm particles could be sintered to >99% density after 6 minutes by spark plasma sintering. A sintering temperature of 750°C = 1023K was used, with a heating rate of 80°C/min, and a holding pressure of 50MPa was applied. Ignoring the differences in the process for now, the present data would predict a time of $t(\rho = 0.99)(\frac{1\mu\mathrm{m}}{0.008\mu\mathrm{m}})^{3.27} = 1726\mathrm{s}$ or roughly 30 minutes.

The difference of course comes from the fact that the process wasn't replicated, since this is a simple after-the-fact comparison. The higher temperature and pressure suggest much quicker densification, but the "slow" heating relative to the simulation, which instantly obtains its sintering temperature, can counteract this effect.

In [ ]:
