# Supplementary material for: Revealing process and material parameter effects on densification via phase-field studies
Source: Sci Rep. 2024 Mar 4;14:5350. doi: 10.1038/s41598-024-51915-w (PMC10912692; doi:10.1038/s41598-024-51915-w)
Supplement: Supplementary file 1 — Supplementary Information. [file 41598_2024_51915_MOESM1_ESM.zip › supmat/displacement_model.pdf]

Supplementary material to "Revealing process and material parameter effects on densification via phase-field studies"

This notebook shows that the model as presented is capable of reproducing non-uniform densification resulting from a gradient in grain size. For this, a simple square arrangement of particles is considered with a gradient in grain size in one direction. The strain is shown to be variable in the direction of the gradient, which implies non-uniform densification.

References:

- Seiz2023b: https://doi.org/10.1007/s10853-023-08859-9

In [1]:
```
%matplotlib notebook
# notebook backend doesn't work on jupyterlab and widgets seems to die
# exclusively on binder, but works fine locally
# hence if you want interactivity in the plots:
# restart kernel, change the inline of the above to notebook and
# go to the notebook view via changing the url from
# https://hub.ovh2.mybinder.org/whatever/lab?
# to
# https://hub.ovh2.mybinder.org/whatever/tree/
import matplotlib.pyplot as plt
import matplotlib as mpl
import numpy as np
import glob
import matplotlib.patches as patches
import scipy
import scipy.sparse.linalg as linalg
import scipy.sparse as sparse
import random
import re
mpl.rcParams.update({'font.size': 16})
mpl.rcParams['figure.figsize'] = (8,4)
X, Y = 0, 1
dirnames = {X: "X", Y: "Y"}
from helpers_dispmodel import *
```

What follows is a bit of setup. For geometry variation, adjust the parameters passed to makeGradiatedPacking.

In [2]:
```
def makeGradiatedPacking(rmin, nmax, steps, layercount):
    """
    Makes a rectangular packing, possibly with a gradient in grain size in y

    rmin: starting radius
    nmax: number of particles to set in the smallest particle layer
    steps: number of particle radii to use; sizes will be r ~ rmin * 2**(n-1
    layercount: number of layers to set for each unique particle radius

    """
    if nmax < 2**(steps-1):
        print("increase nmax or you will be missing steps!")
```

```python
        return np.nan, np.nan, np.nan
    nump = 0
    scalef = 2 # produces weird results for n!=2, generalization pending
    for i in range(steps):
        nump += layercount * nmax * (scalef)**(-i)
        # half the number of particles per step
    #layer0x = np.linspace(0, rmin*nmax, nmax)
    #layer0y = np.zeros(nmax)
    nump = int(np.ceil(nump))
    rad = np.zeros(nump)
    pos = np.zeros((DIM, nump))
    pcount = 0
    yoff = 0
    xoff = 0
    for i in range(steps):
        sizefac = scalef**i
        for lc in range(layercount):
            for k in range(nmax // int(sizefac)):
                radius = rmin * sizefac
                px = k * 2 * radius + xoff
                py = yoff

                rad[pcount] = radius * 1.1 # ensure contacts
                pos[:,pcount] = [px, py]
                pcount += 1
            yoff +=  radius * 2
        yoff += radius
        xoff += radius
    return rad, pos, nump
```

In [3]:
```python
DIM= 2
rad, pos, totp = makeGradiatedPacking(1, 20, 3, 3) # rectangular, gradiated
#rad, pos, totp = makeGradiatedPacking(1, 16, 1, 9) # rectangular packing of
```

In [4]:
```python
# quick 2D visualization of the packings w/ overlap
# this has limited applicability to the 3D packings, so it's above reading i
fig, ax = plt.subplots(figsize=(6,6))

#ax.scatter(pos[0], pos[1])
xmin, xmax = np.min(pos[0,:])-np.max(rad)*1.01, np.max(pos[0,:])+np.max(rad)
ymin, ymax = np.min(pos[1,:])-np.max(rad)*1.01, np.max(pos[1,:])+np.max(rad)
ax.set_xlim(xmin, xmax)
ax.set_ylim(ymin, ymax)
note = False # True: show particle number
pps = range(totp) #range(totp) if totp < 200 else np.random.randint(0,totp,s
for p in pps:
    #print(posen[0][p], posen[1][p], raden[p])
    pa = patches.Circle((pos[0][p], pos[1][p]), radius=rad[p], fill=False)
    ax.add_patch(pa)
    if note:
        ax.annotate(str(p+1), (pos[0][p], pos[1][p])
               # , fontsize=30
                   )
fig.tight_layout()
```

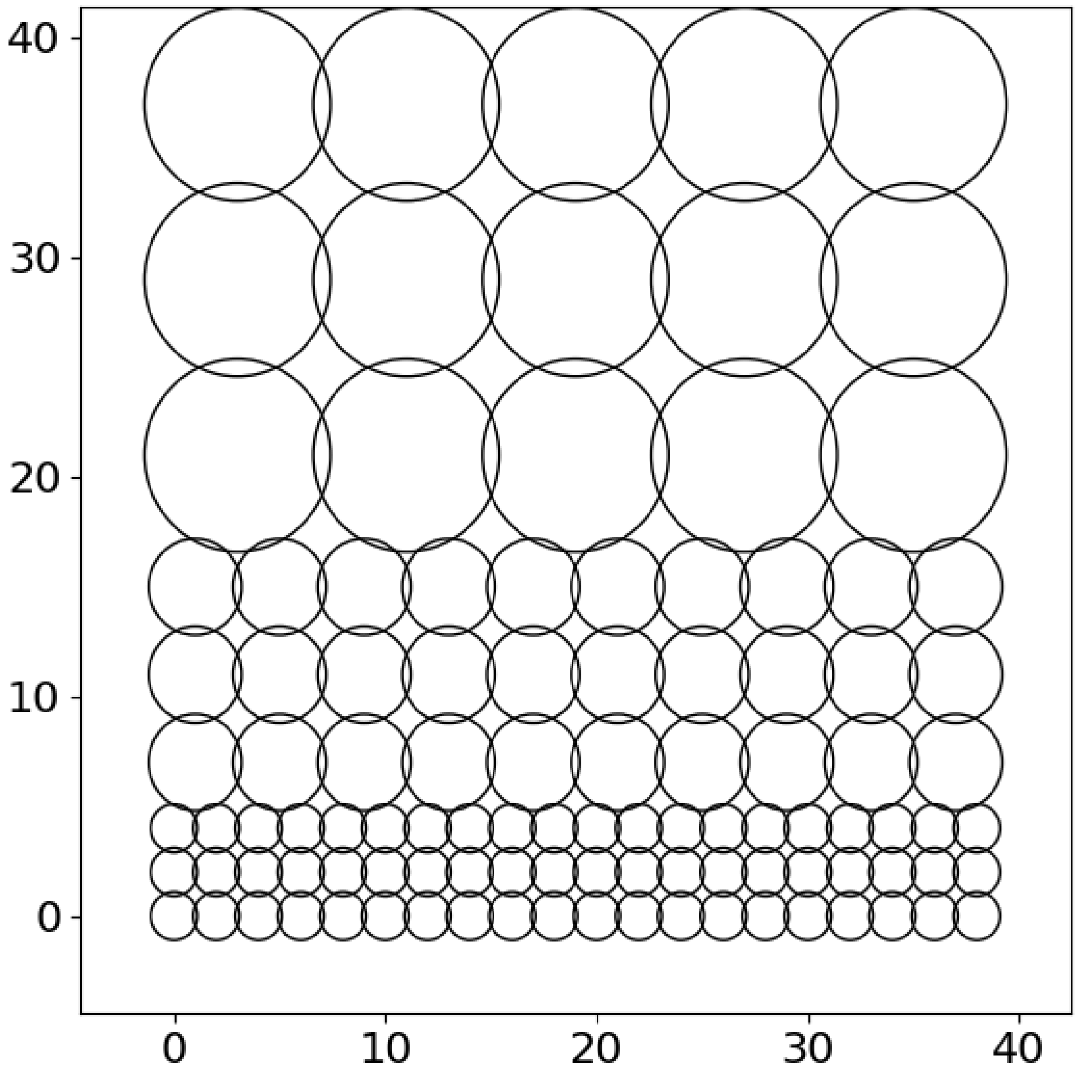

In [5]:
```
contacts, kd = getContacts_kd(pos, rad, varrad=True)
```

In [6]:
```
try:
    if testConnectivity(contacts):
        print ("ok to proceed")
    else:
        print("might be missing some connections, so some displacements will
except Exception as e:
    print(e)
```

```
ok to proceed
```

In [7]:
```
sigma = 0*0.1 # 0 standard dev to remove random influence
mu=0.05 # mu set such that particles move "reasonably", i.e. less than a par
posdep = lambda x: 0 #0
rdep = lambda r: mu/r # curvature # one could in take this to be gamma/r
```

In [8]:
```
def buildSystem_rdep(pos, rad, contacts, rhsmid=1.0, rhsspread=0.25, posdep=
    """
    Builds the system Cu=\Delta u as described in Seiz2023b, now with a radi
```

```
    \Delta u = rhs is assumed to be a normal distribution, with the directio
    rhsmid, rhsspread are the position (mean) and scale (stddev) of the norm
    posdep allows passing a single-argument function to give a spatial bias 
    Its argument is assumed to be, for simplicity, only the x position of th

    Returns the system matrix C as a list of sparse matrices per dimension a
    """
    dim, nump = pos.shape
    dokmats = []
    gbcounter = 0
    gbcount = getGBcount(contacts)
    rhsen = np.zeros((dim, gbcount))
    rhsscale = 1  # arbitrary number which scales the random distribution

    if posdep == None:  # avoid mutable default argument troubles
        posdep = lambda x: 0

    if rdep == None:  # avoid mutable default argument troubles
        rdep = lambda x: 0

    for d in range(dim):
        dokmats.append(sparse.dok_matrix((gbcount, 1 * nump)))
    for p in range(nump):
        for gbp in contacts[p]:
            # we assume that the grain boundary normal is given by the conne
            # this is just for the notebook, whereas in the PF code the grai
            # is found by averaging grad(phia)-grad(phib) over each ab-inter
            gbtot = pos[:, p] - pos[:, gbp]
            norm = np.linalg.norm(gbtot, 2)
            gbtot /= norm

            rnd = np.random.normal(rhsmid, rhsspread)
            # a bias by the position *will* cause the fit solution to deviat
            # a nonlinear profile
            rnd += posdep(pos[0, p])  # bias by 0th position = x
            rnd += rdep(0.5*(rad[p] + rad[gbp])) # take it as average betwee
            for d in range(dim):
                gbn = gbtot[d]
                dokmats[d][gbcounter, p] = -1 * np.sign(gbn)
                dokmats[d][gbcounter, gbp] = 1 * np.sign(gbn)

                rhsen[d][gbcounter] = rnd * rhsscale * np.abs(gbtot[d])
            gbcounter += 1
    cscmats = [x.tocsc() for x in dokmats]
    return cscmats, rhsen
```

In [ ]:

In [9]:

```
cmat, r = buildSystem_rdep(pos, rad, contacts, rhsmid=mu, rhsspread=sigma, p
                                 rdep = rdep)
inis = ini_pos(pos)
```

We can now solve for the displacement in the gradiated structure. Note that an explicit dependence of the RHS (= displacement jump magnitude between grains) on the particle

radius was employed to approximate the effect of a smaller capillary pressure at larger particle radii.

In the first plot below we can see that as one would basically expect, larger particles move less (smaller arrows). The differential effect can be shown better by plotting both the reference and displaced configuration together.

In [10]:
```
displ = np.zeros((2, totp))
for sdir in [X, Y]:
    ds = linalg.lsqr(cmat[sdir], r[sdir], x0=inis[sdir])[0]
    ds -= np.average(ds, weights=np.pi * rad**2)
    displ[sdir,:] = ds

    xmin, xmax = np.min(pos[sdir]), np.max(pos[sdir])
    bincount = 10
    binedges = np.linspace(xmin, xmax, bincount)


fig, ax = plt.subplots()
ax.set_xlabel("x position / -")
ax.set_ylabel("y position / -")
ax.scatter(pos[0], pos[1], s=rad**2+10, alpha=0.5)
# NB the circles here are *not* to scale, just to give the idea how large th
pps = range(totp) if totp < 200 else np.random.randint(0,totp,size=200)
# comment out the if part of the above assignment to see all arrows
for p in pps:
    dx = displ[0,p]
    dy = displ[1,p]
    ax.arrow(pos[0][p], pos[1][p], dx*0.9, dy*0.9, width=.01)
fig.tight_layout()
```

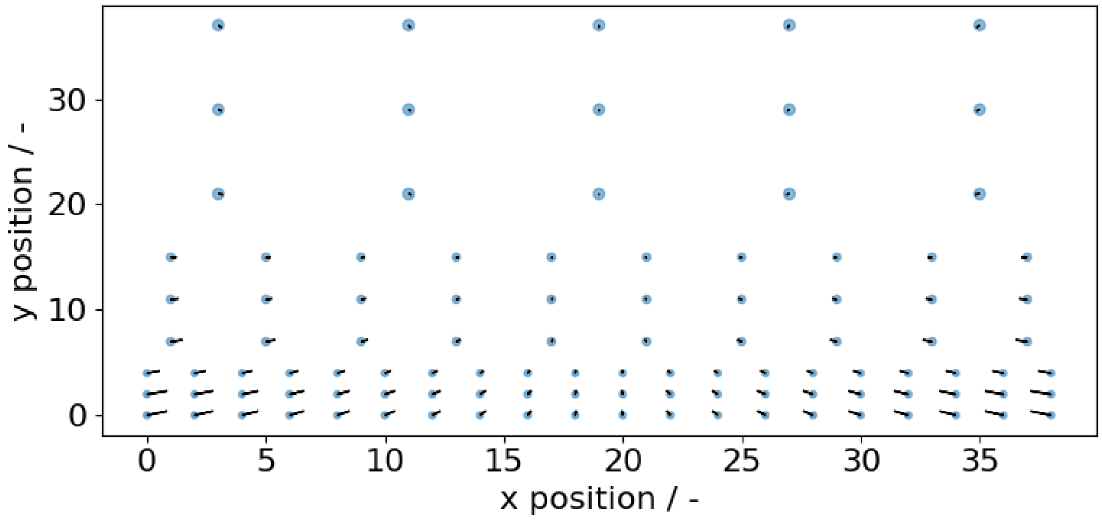

The reference and displaced configurations are compared in two ways: First is simply plotting both in two parallel boxes, second is overlapping them in the same box with different colours (black = reference, red = displaced). In both cases differential densification is evident.

In [11]:

```
# quick 2D visualization of the packings w/ overlap
# this has limited applicability to the 3D packings, so it's above reading i
fig, axen = plt.subplots(1,2, figsize=(10,5))
axen[0].set_title("reference configuration")
axen[1].set_title("displaced configuration")
#ax.scatter(pos[0], pos[1])
ifac = 1.01
xmin, xmax = np.min(pos[0,:])-0.5*np.max(rad)*ifac, np.max(pos[0,:])+0.5*np.
ymin, ymax = np.min(pos[1,:])-np.max(rad)*ifac, np.max(pos[1,:])+np.max(rad)
for i, ax in enumerate(axen):
    dispbool = i == 1
    ax.set_xlim(xmin, xmax)
    ax.set_ylim(ymin, ymax)
    note = False # True: show particle number
    pps = range(totp) #range(totp) if totp < 200 else np.random.randint(0,to
    for p in pps:
        #print(posen[0][p], posen[1][p], raden[p])
        pa = patches.Circle((pos[0][p]+displ[0,p]*dispbool, pos[1][p]+displ[
                            radius=rad[p], fill=False)
        ax.add_patch(pa)
        if note:
            ax.annotate(str(p+1), (pos[0][p], pos[1][p])
                    # , fontsize=30
                       )
fig.tight_layout()
fig.savefig("ref-displ.pdf", bbox_inches="tight")
```

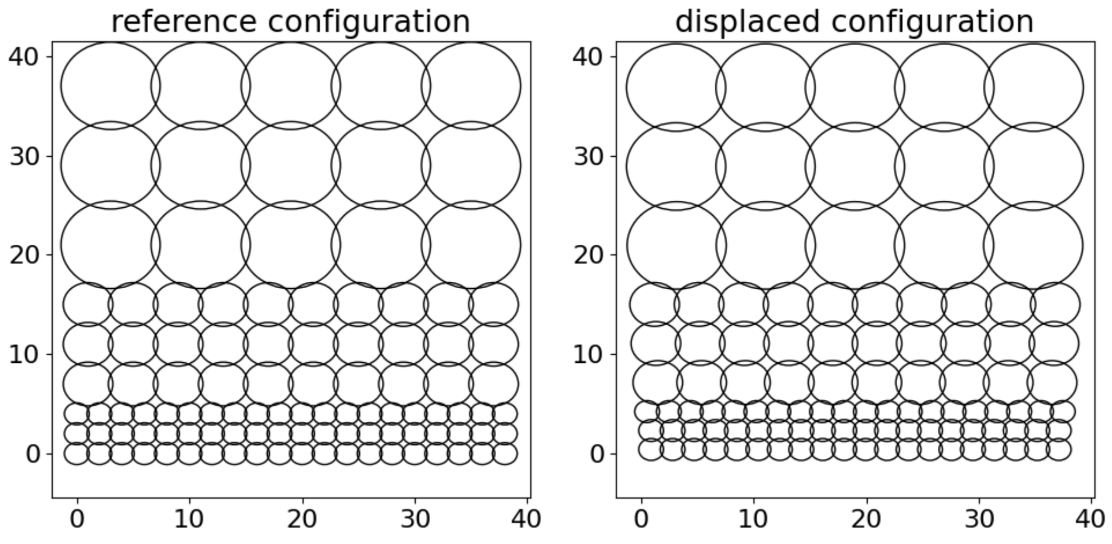

In [12]:

```
# quick 2D visualization of the packings w/ overlap
# this has limited applicability to the 3D packings, so it's above reading i
fig, ax = plt.subplots(figsize=(6,6))

#ax.scatter(pos[0], pos[1])
xmin, xmax = np.min(pos[0,:])-np.max(rad)*0.5, np.max(pos[0,:])+np.max(rad)*
ymin, ymax = np.min(pos[1,:])-np.max(rad)*1.01, np.max(pos[1,:])+np.max(rad)
ifac = 1.01
xmin, xmax = np.min(pos[0,:])-0.5*np.max(rad)*ifac, np.max(pos[0,:])+0.5*np.
```

```python
ymin, ymax = np.min(pos[1,:])-np.max(rad)*ifac*0.5, np.max(pos[1,:])+np.max(

ax.set_xlim(xmin, xmax)
ax.set_ylim(ymin, ymax)



note = False # True: show particle number
pps = range(totp) #range(totp) if totp < 200 else np.random.randint(0,totp,s
for p in pps:
    #print(posen[0][p], posen[1][p], raden[p])
    pa = patches.Circle((pos[0][p], pos[1][p]), radius=rad[p], fill=False)
    pa2 = patches.Circle((pos[0][p]+displ[0,p], pos[1][p]+displ[1,p]),
                         radius=rad[p], fill=False, color="red")
    ax.add_patch(pa)
    ax.add_patch(pa2)
#ax.plot([], [], color="black", label="reference")
#ax.plot([], [], color="red", label="displaced")
#fig.legend()
fig.tight_layout()
fig.savefig("ref-displ.pdf", bbox_inches="tight")
```

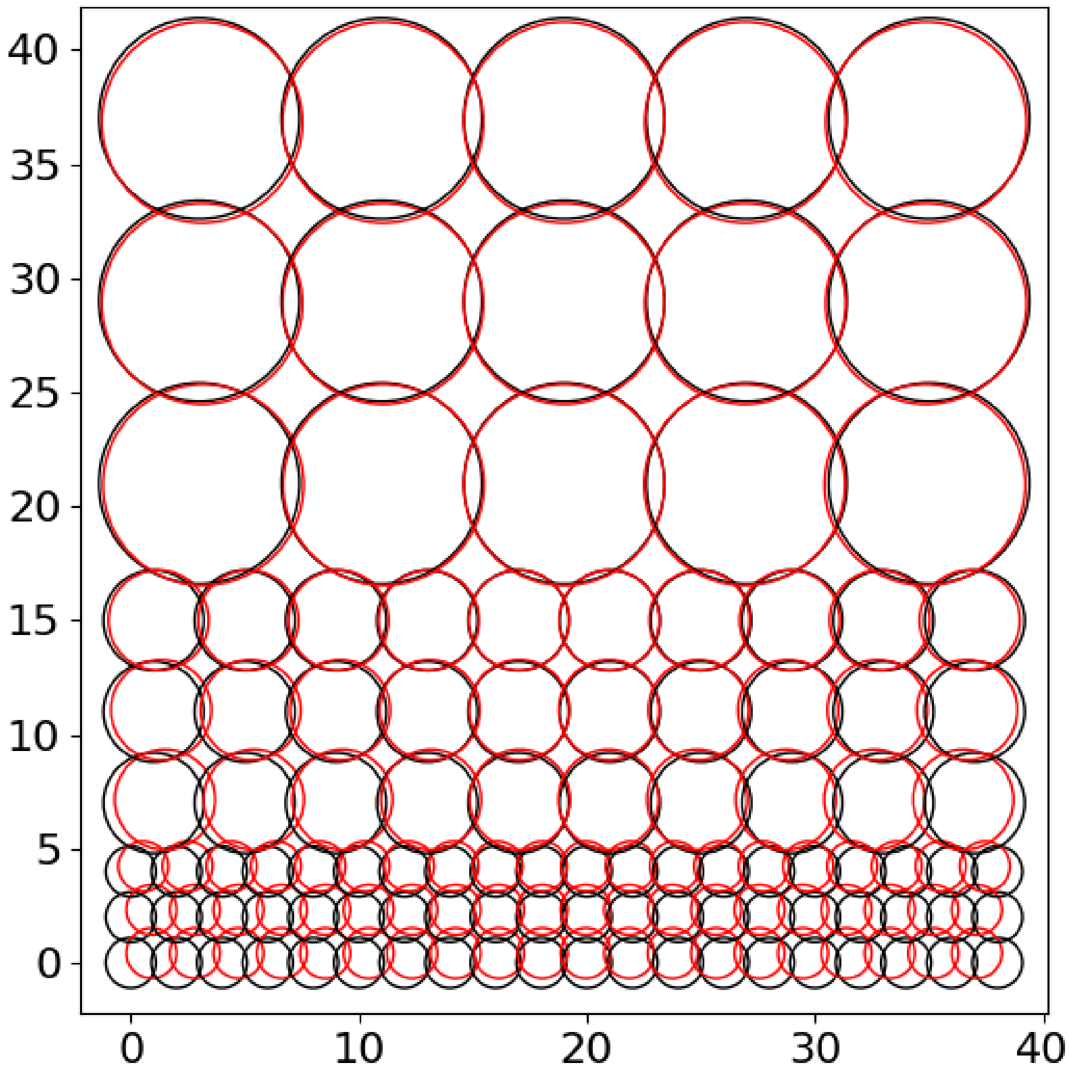

To make this more quantitative the displacement and strains are considered next. A zero order approximation is to simply look at the maximum displacement on the lowest and highest layer (smallest and largest particles) and compare their displacements.

In [13]:
```
uniqy = np.unique(pos[1,:])
miny, maxy = np.min(uniqy), np.max(uniqy)
us = []
for ypos in [miny, maxy]:
    filt = pos[1,:] == ypos
    ldispl = np.max(np.abs(displ[0,filt]))
    us.append(ldispl)
    print(ypos, ldispl)
```

```
0.0 0.9068181819319644
37.0 0.12272727272462536
```

In [14]:
```
us[0] / us[1]
```

Out[14]: 7.3888888899753935

And we can observe a factor of ~7.4 more displacement in the bottom layer, as a result of two doublings of particle size. To make this a bit better spatially resolved, a discrete field is filled by the observed displacements which gives us a general idea of the displacement distribution in space.

In [15]:
```
# sample displacement into discrete field
# would be nicer to get a continuous field instead of jumps
xmin, xmax = np.min(pos, axis=1), np.max(pos, axis=1)
deltax = 0.1
isize = ((xmax-xmin)/deltax).astype(np.int32)
fieldx = np.zeros(isize)
fieldy = np.zeros(isize)
def particle2field(x, y):
    return int(x/deltax), int(y/deltax)
def field2particle(x, y):
    return x*delta, y*deltax
```

In [16]:
```
def fillCircle(field, cx, cy, r, value):
    cent = np.array([cx, cy])
    mins = cent-r
    maxs = cent+r
    rsq = r**2
    sh = field.shape
    # sanity checks
    for i in range(2):
        if mins[i] < 0 :
            mins[i] = 0
        if maxs[i] >= sh[i]:
            maxs[i] = sh[i]
    for x in range(mins[0], maxs[0]):
        for y in range(mins[1], maxs[1]):
            dloc = (x-cx)**2 + (y-cy)**2
            if (dloc <= rsq):
                field[x,y] = value
```

In [17]:
```
for i in range(totp):
    # disgusting O(n * r^2) complexity
    px, py = pos[:,i]
    fr = int(rad[i]/deltax)
    fx, fy = particle2field(px, py)
    fillCircle(fieldx, fx, fy, fr, displ[0,i])
    fillCircle(fieldy, fx, fy, fr, displ[1,i])
```

In the following displacement map a gradient in y (right) is quite evident, though whether it is linear or not is not so visible; hence a cut is made to show the actual profile.

In [18]:
```
# gradient is somewhat visible here, but becomes clearer in a cut
fig, ax = plt.subplots()
ax.imshow(fieldy)
```

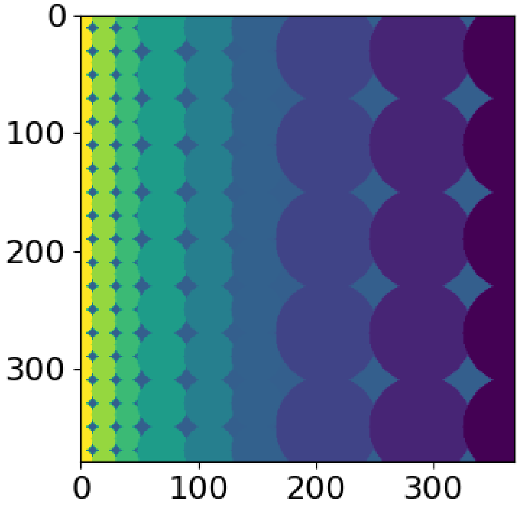

Out[18]: <matplotlib.image.AxesImage at 0x7f8269187a60>

The displacement in (x, y), when plotted over the y-coordinate, show a position dependence. This is the result of adding the radius dependence to the system, as the RHS is now dependent on it and hence the magnitude of the displacement jump.

NB: The plot is over the field coordinate which is the particle coordinate divided by the field spacing (deltax). Furthermore, the jumps to 0 come from the discrete representation of the displacement field --- where there are no particles, the displacement is undefined and set to zero.

Note that when plotted over the x-coordinate the gradient in y vanishes, because particles within a layer have no displacement jump in y. Contrariwise, they do have a displacement jump in x, but it results in a linear profile.

In [19]:
```
# displacements over the y-coordinate = direction where the grain size gradi
# this yields a gradient in both displacements
fig, ax = plt.subplots()
ax.plot(fieldx[40,:], label="x")
ax.plot(fieldy[40,:], label="y")
ax.legend()
ax.set_xlabel("y position (field) / -")
ax.set_ylabel("displacement / -")
fig.tight_layout()
```

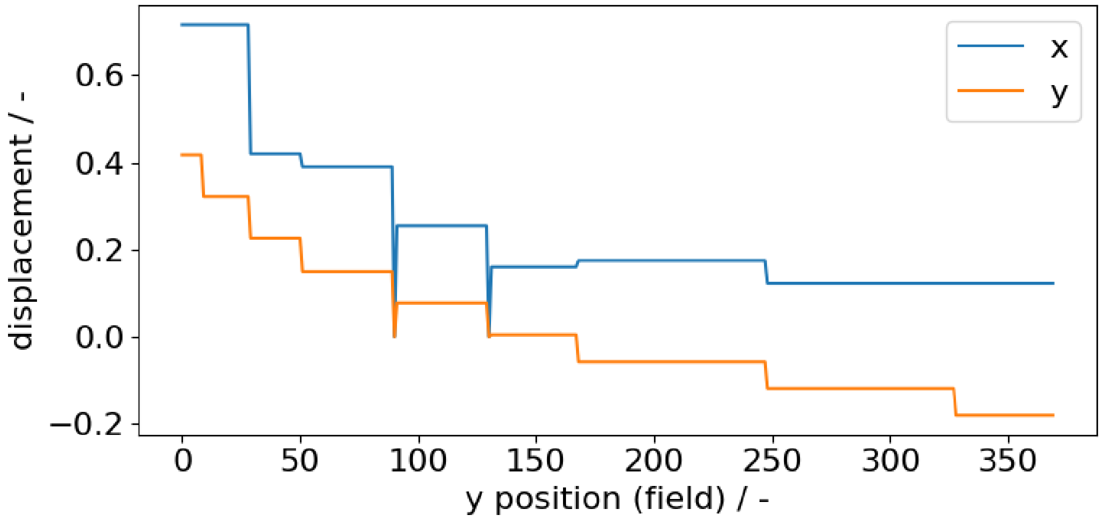

In [20]:
```
# displacements over the x-coordinate = direction where the grain size is co
# constant displacement in y (no sliding) but linear function in x = constar
fig, ax = plt.subplots()
ax.plot(fieldx[:,40], label="x")
ax.plot(fieldy[:,40], label="y")
ax.legend()
ax.set_xlabel("x position (field) / -")
ax.set_ylabel("displacement / -")
fig.tight_layout()
```

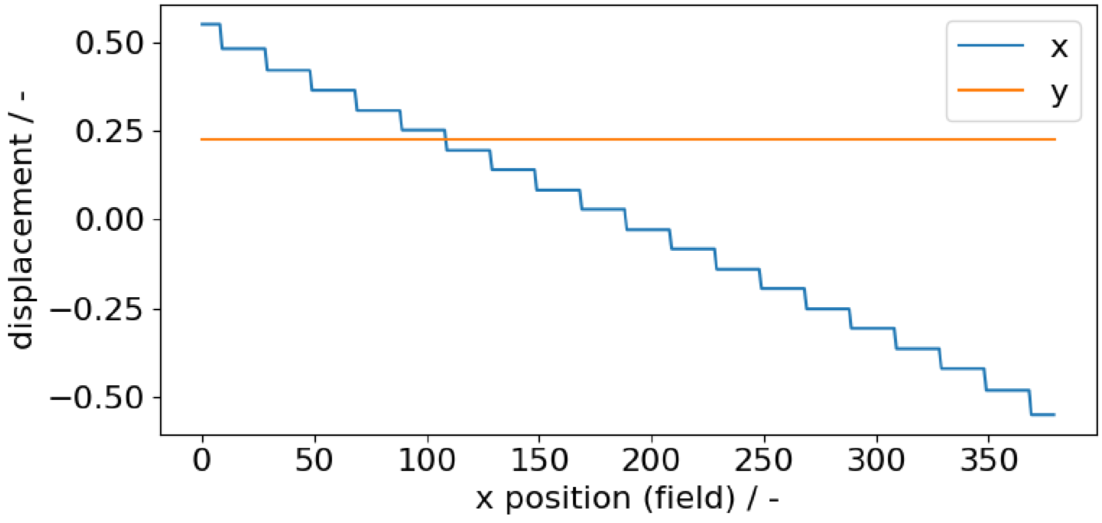

Since the field calculation necessarily have regions of uniform displacement with jumps between them, which makes the calculation of a strain as a gradient of displacement of little value, we move back to the particle picture for the strain calculations.

The first method exploits the layering in the y direction (and to a lesser extent x): Within each y-layer, particles of uniform size are found. Since y=const. only the strain normal to this

(x) is calculated. The same applies for x-"layers", but in this case the layering is more implicit than explicit and the strain in y is calculated.

In [21]:
```
# divide domain into "layers" according to the unique particle positions
# this really only works for the y direction since this is the layering dire
# but by including the particle size information one can also make a stateme
uniqx = np.unique(pos[0,:])
uniqy = np.unique(pos[1,:])
rx, ry = [], []
epseny = []
for y in uniqy:
    filt = pos[1,:] == y
    fdispl = displ[0,filt]
    dudx = np.gradient(fdispl, pos[0,filt])
    epseny.append(np.average(dudx))
    ry.append(np.average(rad[filt]))
epsenx = []
for x in uniqx:
    filt = pos[0,:] == x
    fdispl = displ[1,filt]
    dudx = np.gradient(fdispl, pos[1,filt])
    epsenx.append(np.average(dudx))
    rx.append(np.average(rad[filt]))
rx, ry = [np.array(x) for x in [rx, ry]]
```

In the following scatterplots of the strain over the layer positions are shown. The marker size is scaled by the particle radius on that layer. What is generally observed is that larger particles experience less strain just as one would expect.

In [22]:
```
fig, ax = plt.subplots()
ax.scatter(uniqy, epseny, s=ry**2+20)
ax.set_xlabel("layer position (y) / -")
ax.set_ylabel("avg. $\epsilon_{xx}$ in layer / -")
#ax.scatter(uniqx, epsenx)
ax.grid(True)
fig.tight_layout()
```

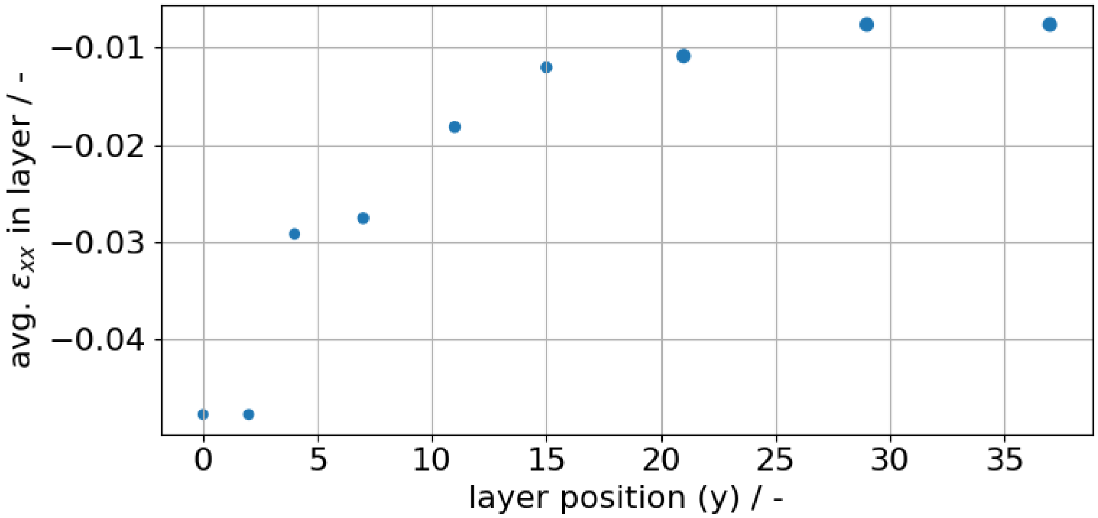

In [23]:

```
fig, ax = plt.subplots()
# again, smaller particles experience higher strain = differential densifica
# note the constant strain levels for each particle size:
# since there is no grain size gradient in x, each x-"layer" densifies unifo
# in contrast to the non-uniform densification resulting from the strain gra
ax.scatter(uniqx, epsenx, s=rx**2+20)
ax.set_xlabel("layer position (x) / -")
ax.set_ylabel("avg. $\epsilon_{yy}$ in layer / -")
ax.grid(True)
#ax.scatter(uniqx, epsenx)
fig.tight_layout()
```

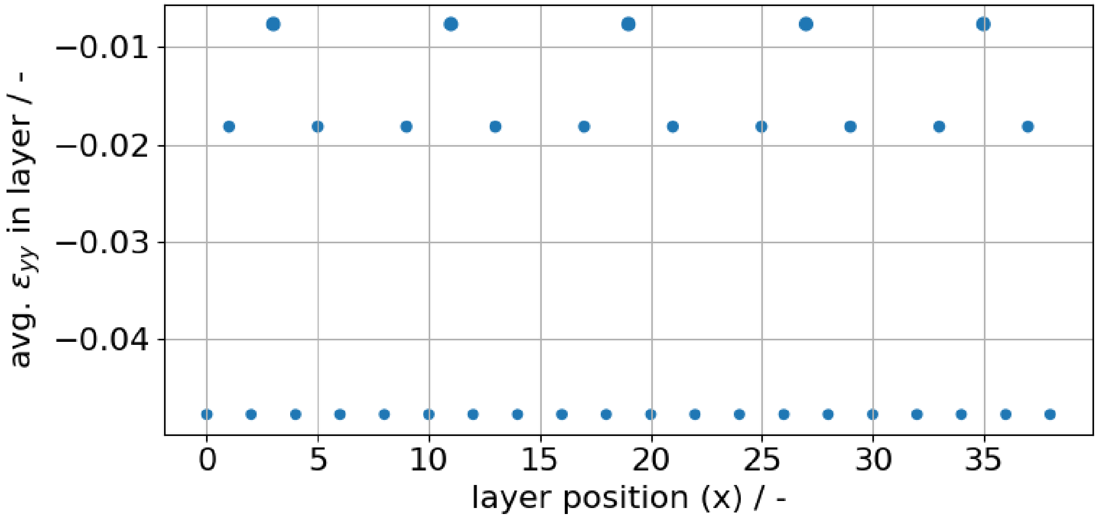

Another method exploiting the layering is to simply take forward differences between the particles, as they are ordered linearly starting from the smallest. This requires filtering out out-of-layer connections, which is actually simply ensuring that there is a nonzero positive difference in the layer direction between the particle's positions (dx > 0). The positivity requirement comes from wrapping from one layer to the next.

In [24]:
```
# forward differences between particle i, i+1 as local strain gauges
# this will jump at the end of each layer
dx = np.diff(pos[0,:])
dy = np.diff(pos[1,:])
# filters remove the jumps and the zero-diffs due to being on the same x or
filt = dx > 0
filt2 = dy > 0

du = np.diff(displ[0,:])
dv = np.diff(displ[1,:])
eps = du[filt] / dx[filt]
epsy = dv[filt2] / dy[filt2]
```

In [25]:
```
# again, a strain gradient is evident here
fig, ax = plt.subplots()
#ax.scatter(pos[0,1:][filt], eps, label="$\epsilon_{xx}$") # not plotted due
# ^ shows roughly constant strain in each layer before going to the next
ax.scatter(pos[1,1:][filt], eps, label="$\epsilon_{xx}$") # not plotted due
ax.scatter(pos[1,1:][filt2], epsy, label="$\epsilon_{yy}$")
ax.set_xlabel("y position / -")
ax.set_ylabel("normal strain / -")
ax.grid(True)
ax.legend()
fig.tight_layout()
fig.savefig("straingradient.pdf", bbox_inches="tight")
```

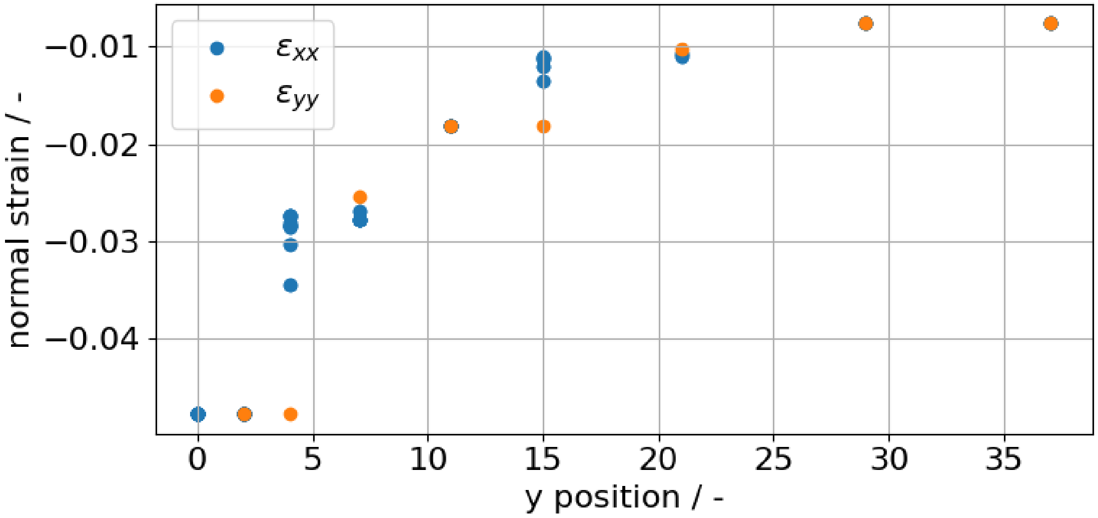

Both calculation methods agree on the magnitude of the strain and its dependence on the y position and therefore grain size. Thus the model is capable of representing differential densification.

It should be noted however that the phase-field implementation uses an approximation method for solving the system, which is solved here by the LSQR algorithm. This comes from two points which are handily ignored in the present notebook:

- The RHS and contact matrix are determined during the phase-field calculation, which is done in parallel on many processes.
- The size of the contact matrix (and associated $O(N^2)$ quantities) explodes if naively stored on each process.

The second point is easily understood to be problematic even for modest system sizes of about 4000 particles: For each pair 40 byte of data needs to be stored, which results in a whopping 640MB memory usage. Not terrible if it's on one process, but if it's mirrored on each process then one is likely to use half the system memory of modern HPC systems for redundant data storage. The data locally calculated during point 1 also needs to be transferred to actually build the matrix and RHS, with a similar amount of communication per process then being necessary, turning the problem into one completely bound by communication.

In order to avoid this, the necessary data for building the matrix/RHS is kept purely locally by assigning a set of grains $[a, a+k]$ to each process and all updates to concerning the matrix/RHS entries for these grains only get transferred to this single process. The process-local storage is kept sparse as well and thus the memory/communication cost is effectively constant in a weak scaling sense. This however makes solving the equation with a standard solver quite troublesome, as each process only has access to its own segmented set of grain information. While there are ways to parallelize this calculation, the expected communication overhead was deemed to be too large (cf. Seiz2023b). Hence a simple linear ansatz is taken for the displacement field --- if it's actually linear, the match is quite good as can be seen in Seiz2023b. However, if it is not, the solution has little to do with the actual displacement field.

In [ ]:
```

```
