# Supplementary figures and images for: Revealing process and material parameter effects on densification via phase-field studies
Source: Sci Rep. 2024 Mar 4;14:5350. doi: 10.1038/s41598-024-51915-w (PMC10912692; doi:10.1038/s41598-024-51915-w)

average neck mean curvature / (nm)<sup>-1</sup>

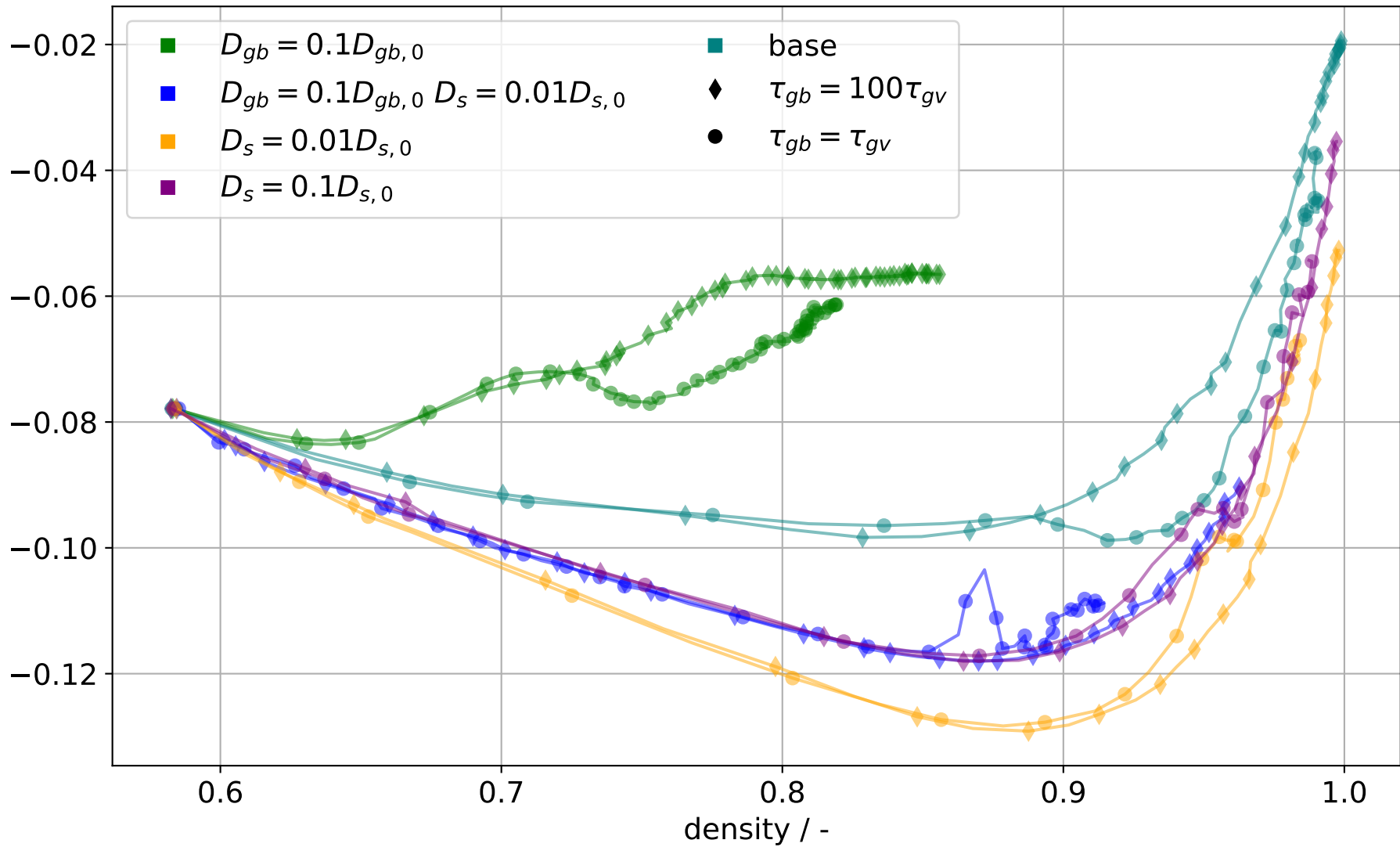

Supplement: Supplementary file 1 — Supplementary Information. [file 41598_2024_51915_MOESM1_ESM.zip › supmat/neckcurvature.pdf]

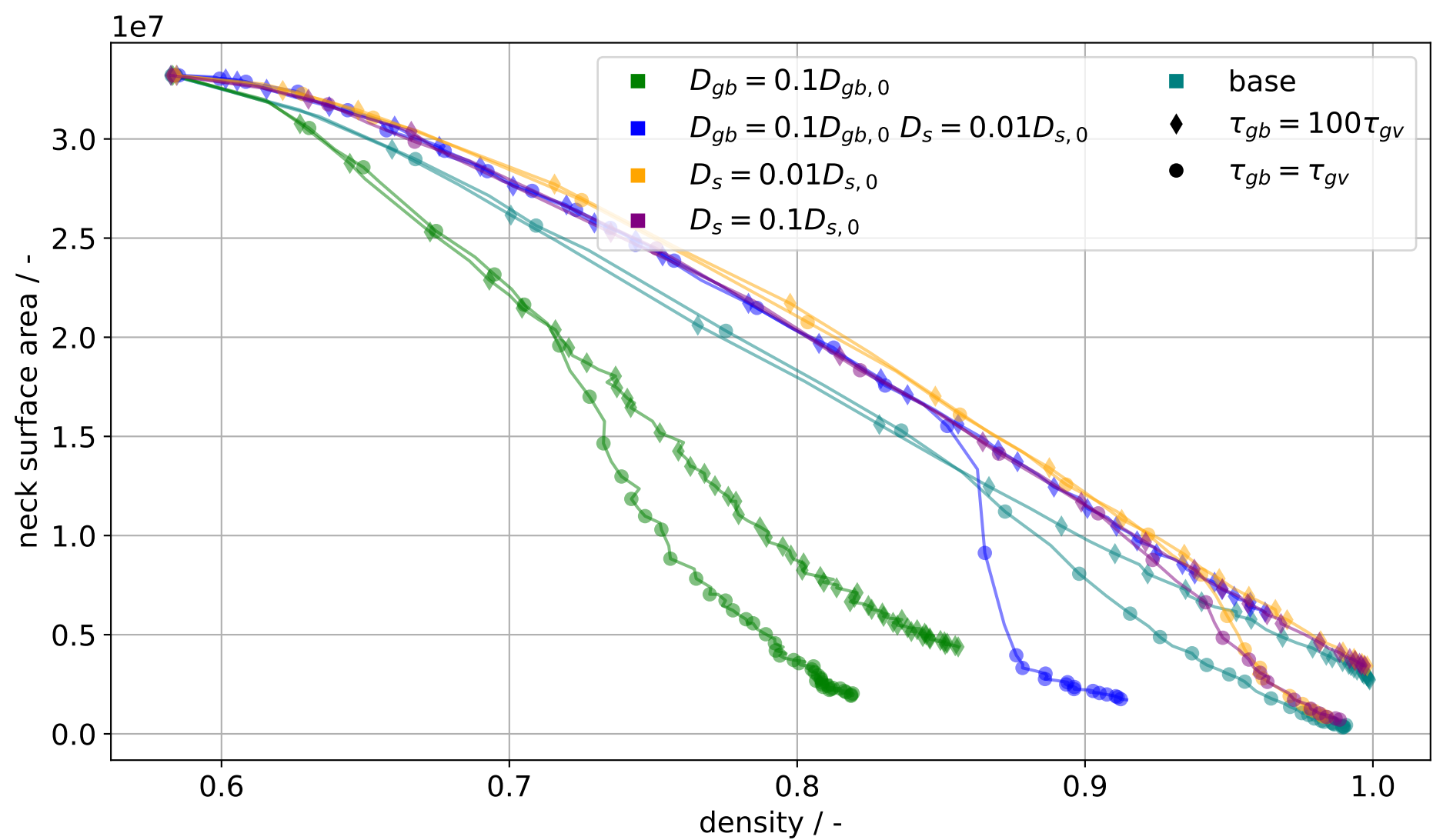

Supplement: Supplementary file 1 — Supplementary Information. [file 41598_2024_51915_MOESM1_ESM.zip › supmat/necksurfarea.pdf]

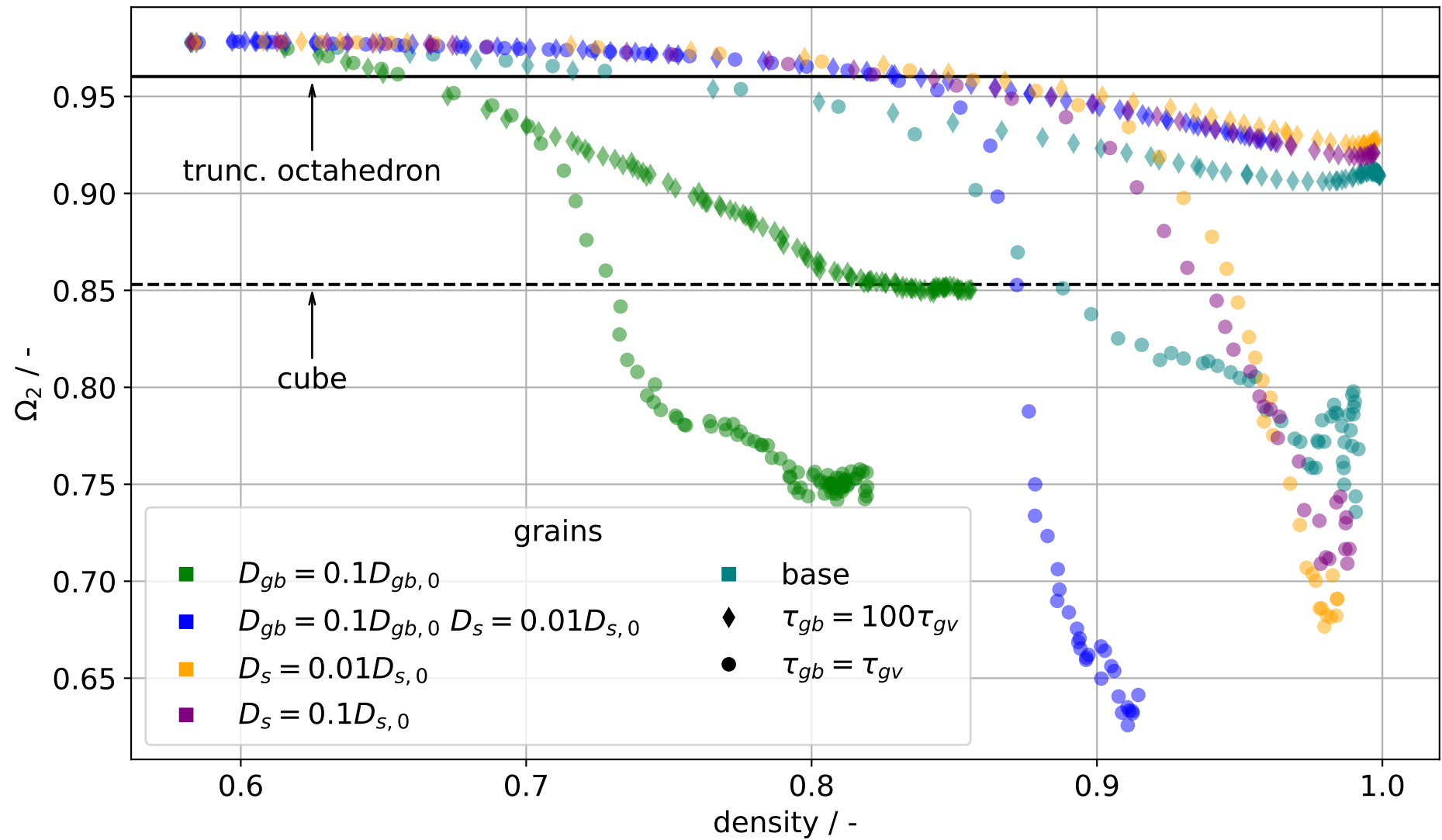

Supplement: Supplementary file 1 — Supplementary Information. [file 41598_2024_51915_MOESM1_ESM.zip › supmat/om2_grains.pdf]

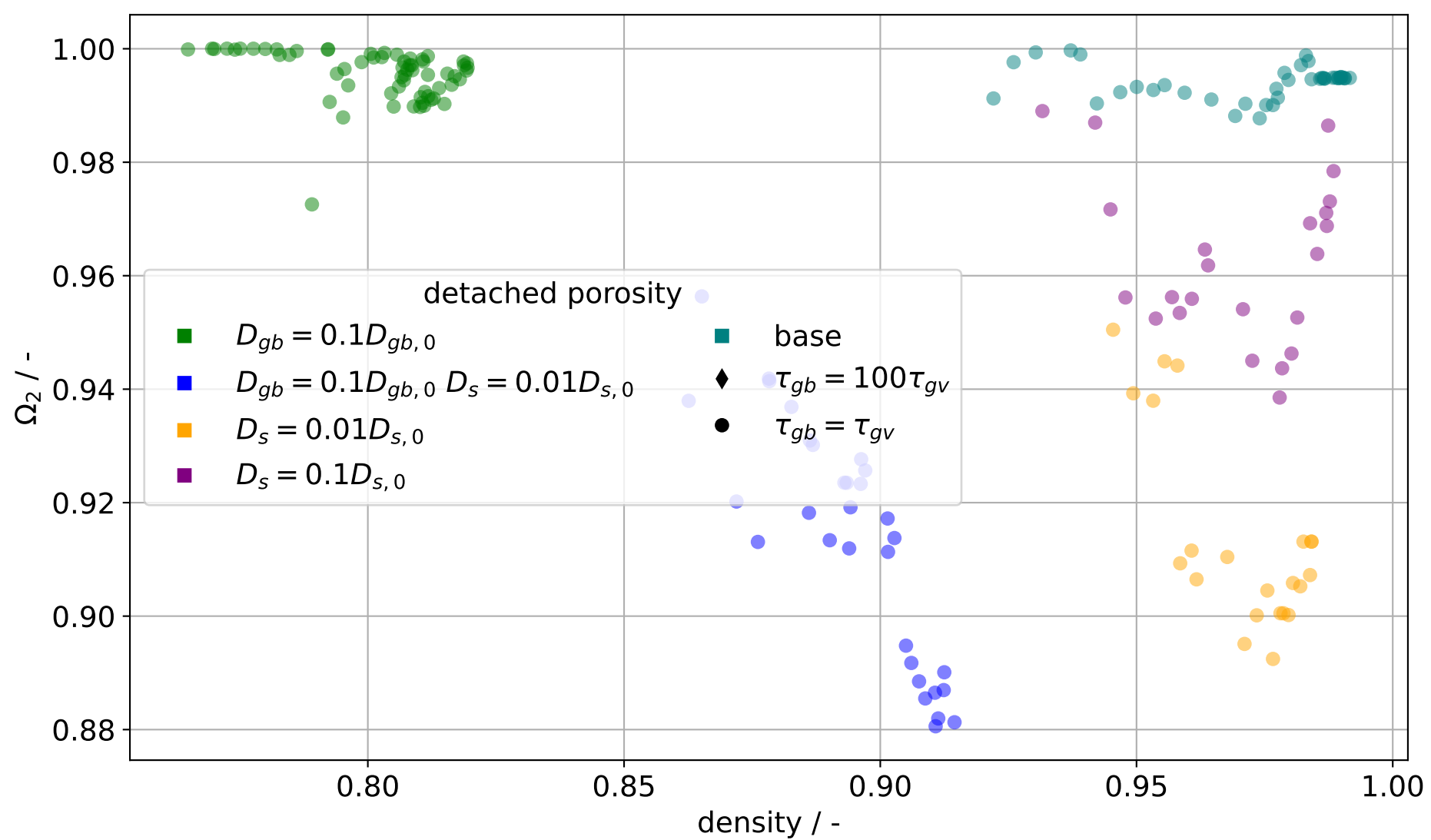

Supplement: Supplementary file 1 — Supplementary Information. [file 41598_2024_51915_MOESM1_ESM.zip › supmat/om2_detached_porosity.pdf]

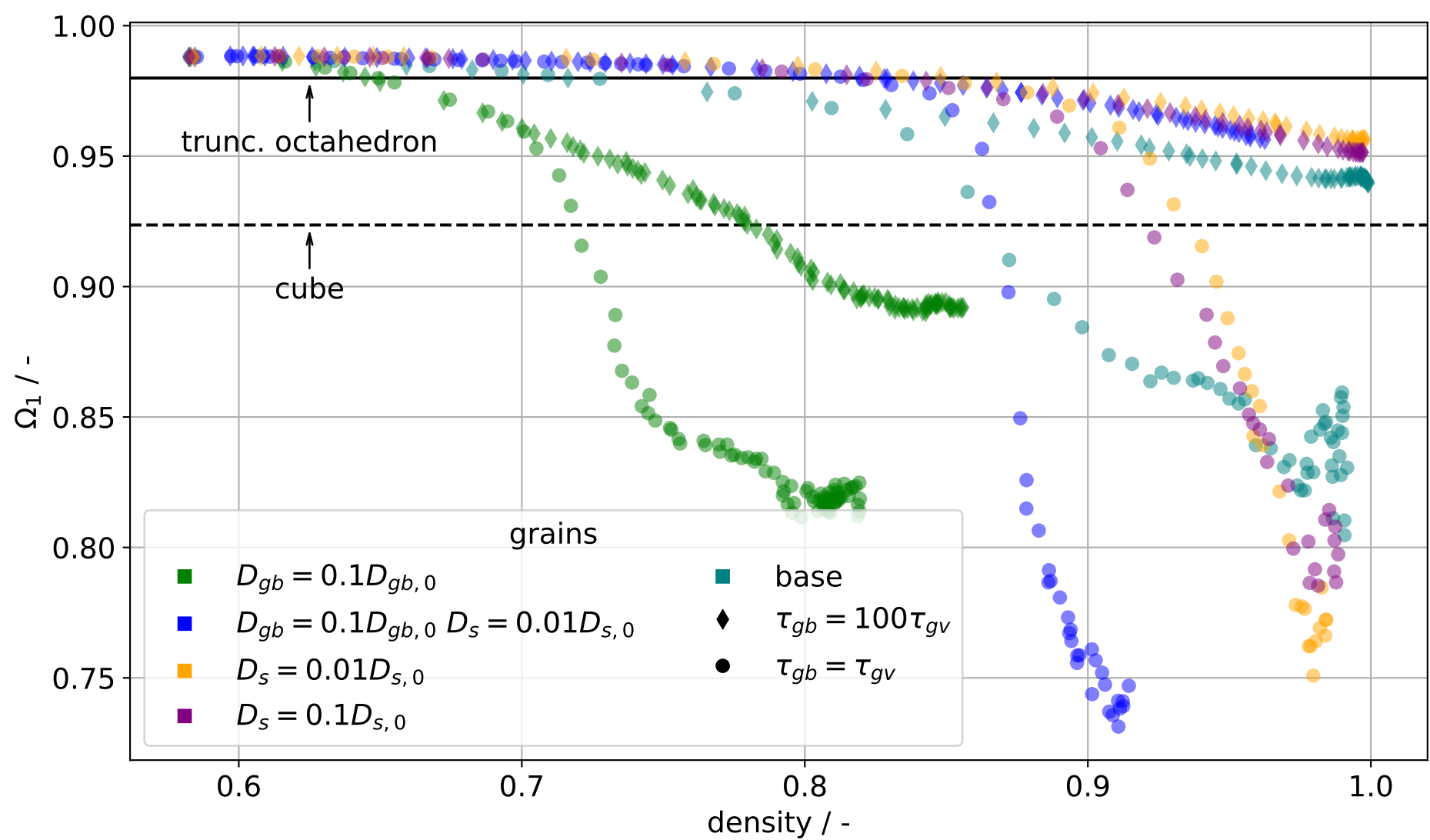

Supplement: Supplementary file 1 — Supplementary Information. [file 41598_2024_51915_MOESM1_ESM.zip › supmat/om1_grains.pdf]

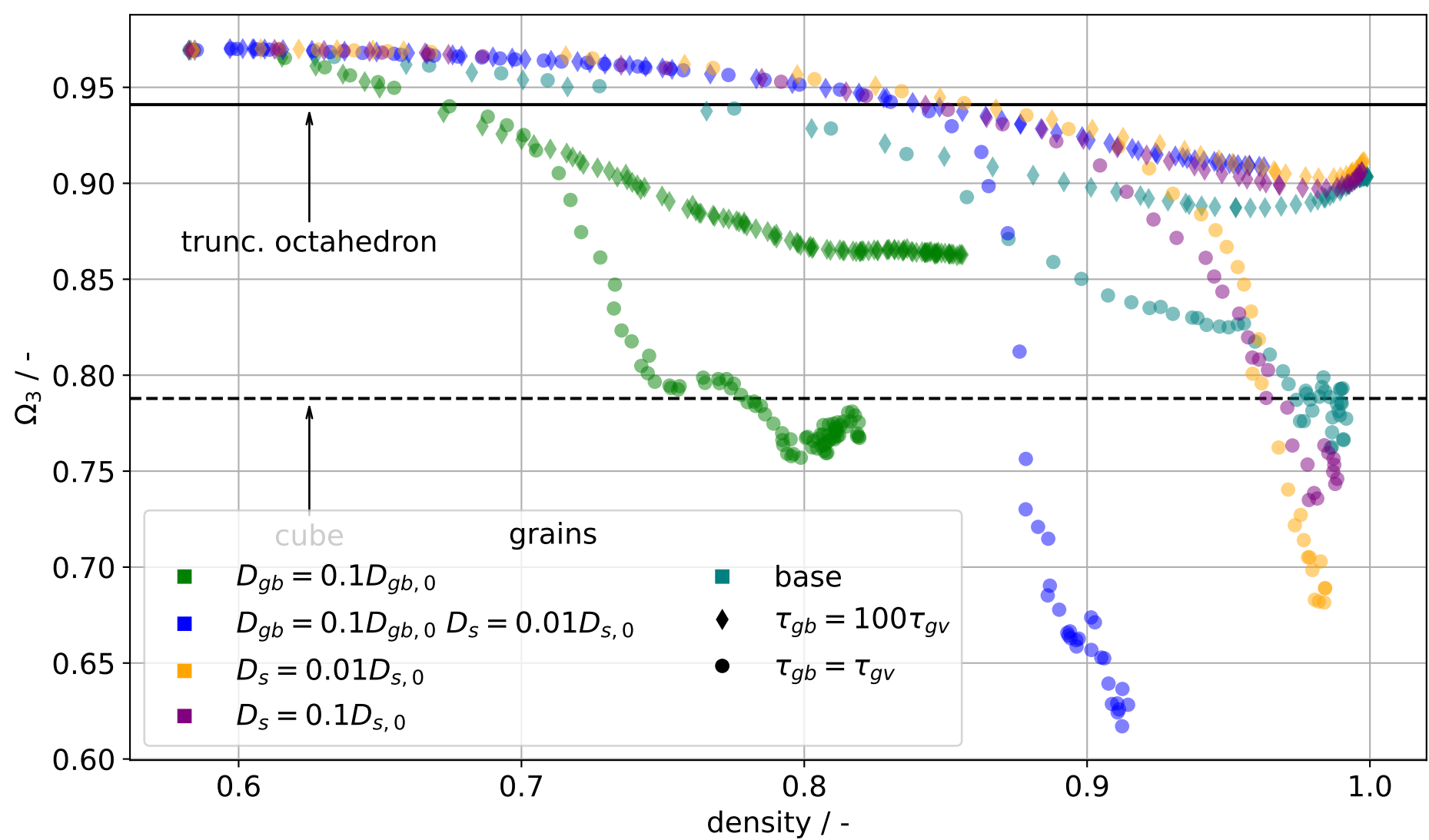

Supplement: Supplementary file 1 — Supplementary Information. [file 41598_2024_51915_MOESM1_ESM.zip › supmat/om3_grains.pdf]

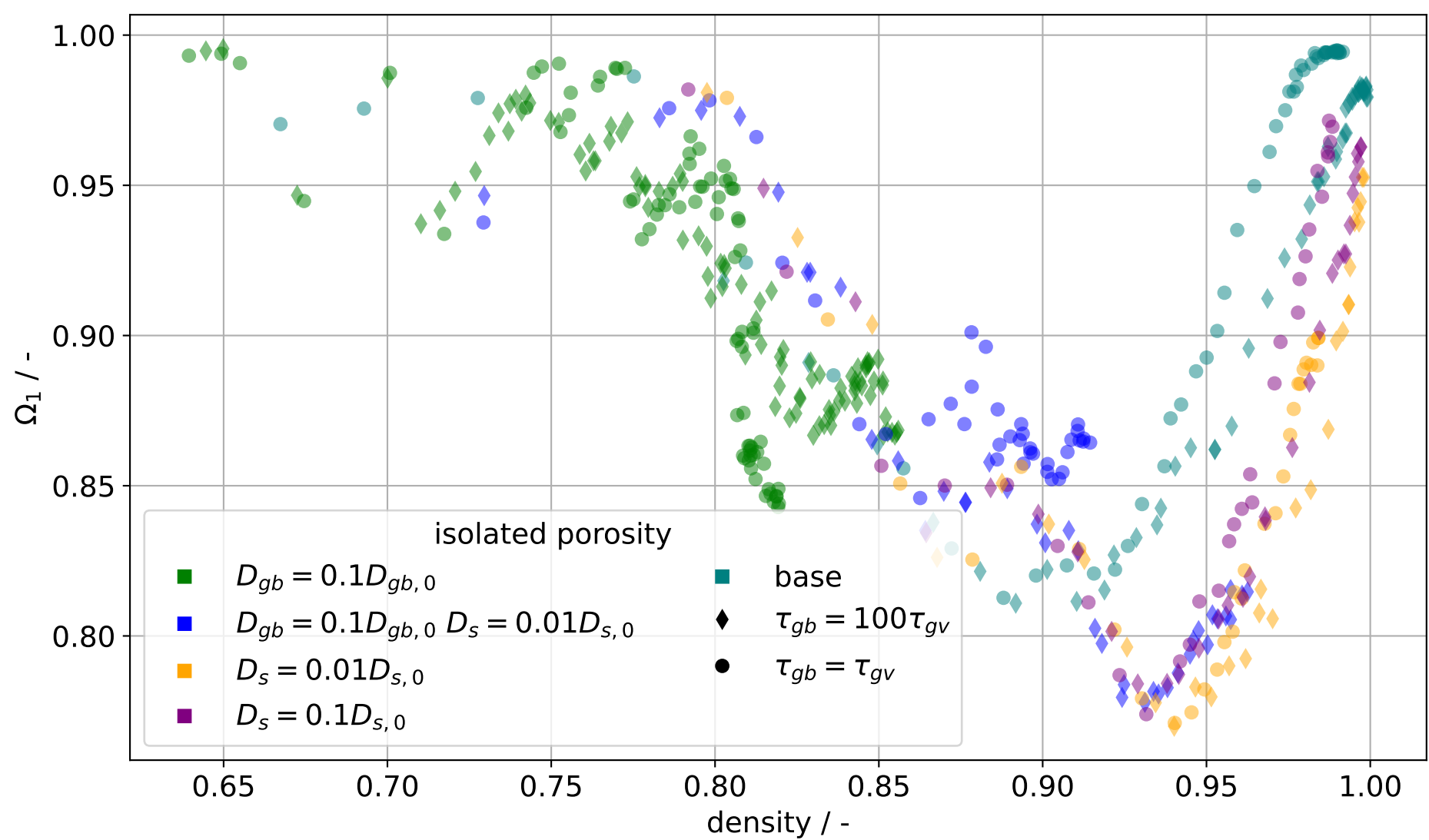

Supplement: Supplementary file 1 — Supplementary Information. [file 41598_2024_51915_MOESM1_ESM.zip › supmat/om1_isolated_porosity.pdf]

reference configuration

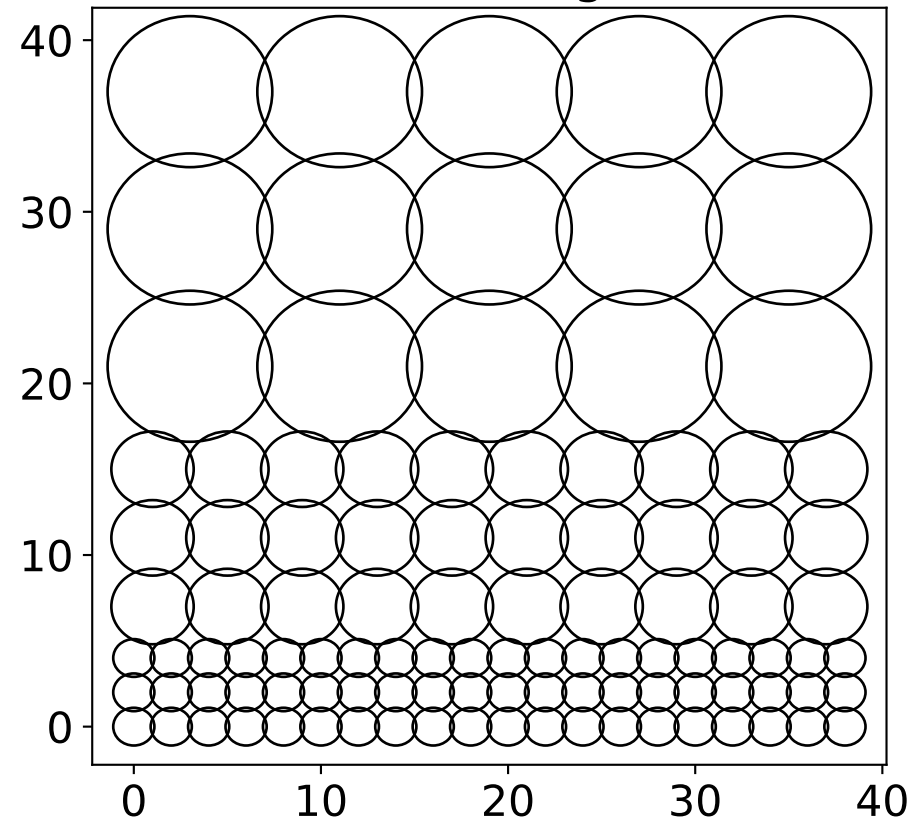

displaced configuration

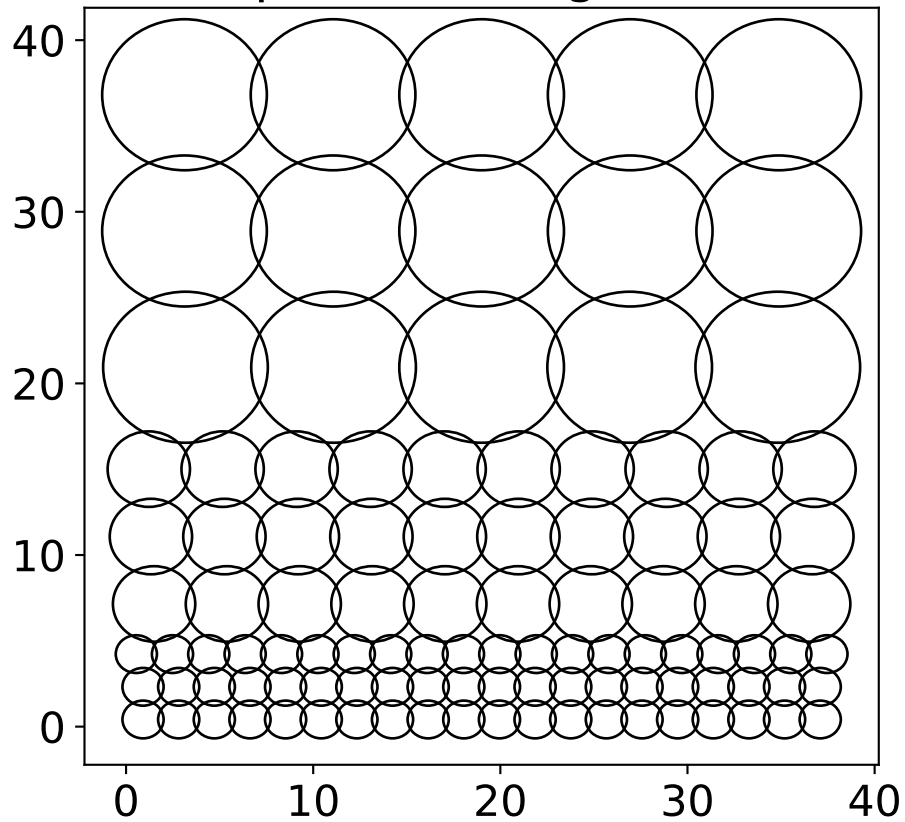

Supplement: Supplementary file 1 — Supplementary Information. [file 41598_2024_51915_MOESM1_ESM.zip › supmat/ref-displ.pdf]

normal strain / -

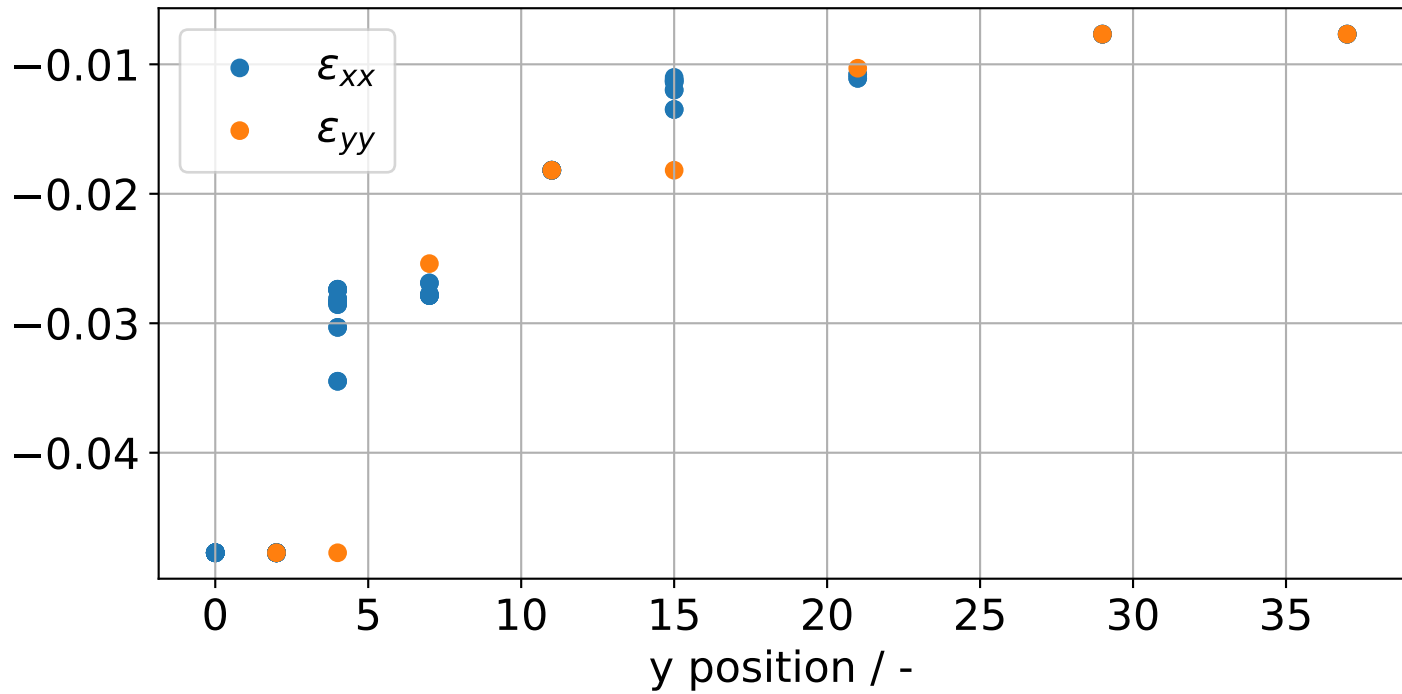

Supplement: Supplementary file 1 — Supplementary Information. [file 41598_2024_51915_MOESM1_ESM.zip › supmat/straingradient.pdf]

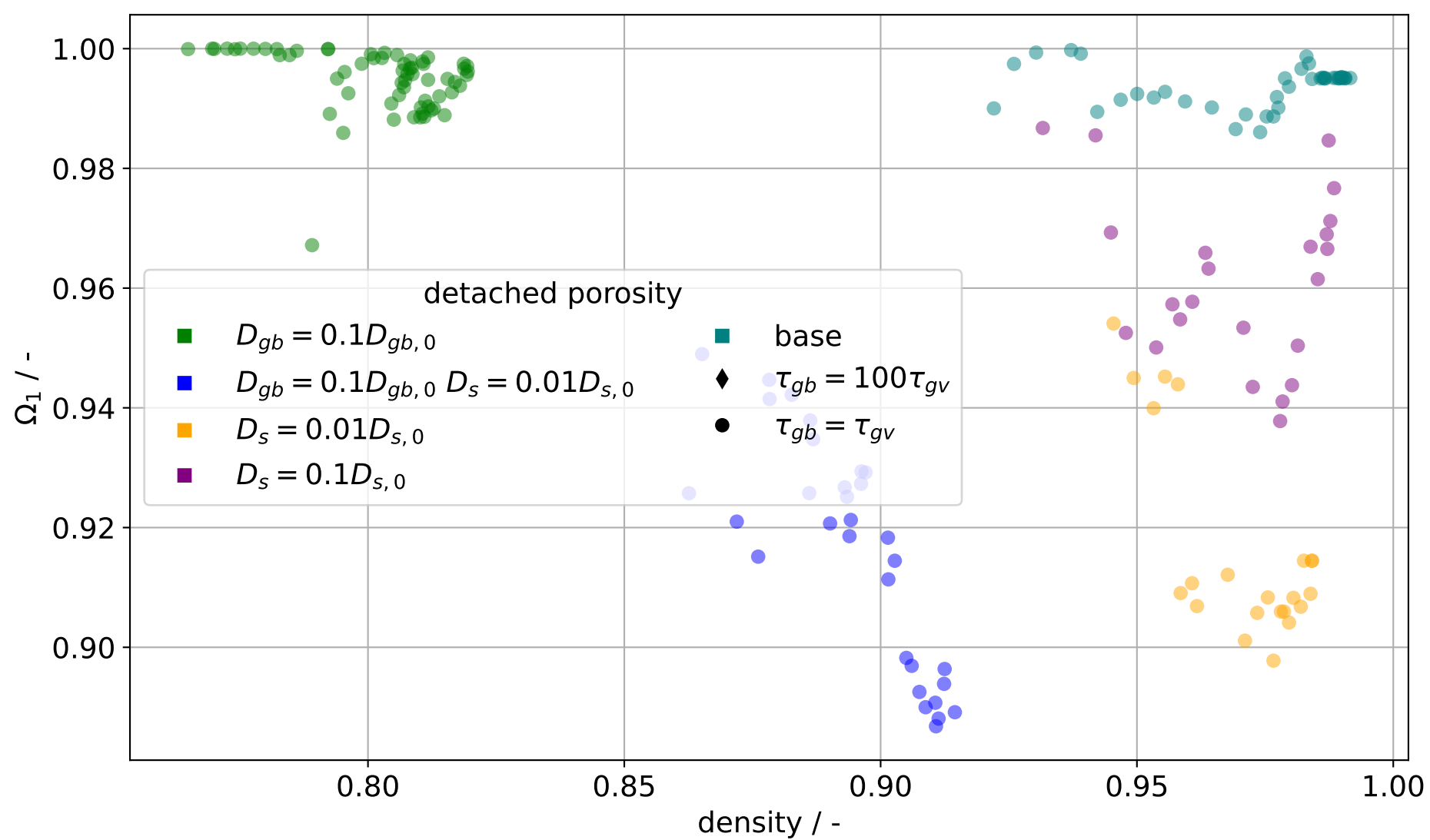

Supplement: Supplementary file 1 — Supplementary Information. [file 41598_2024_51915_MOESM1_ESM.zip › supmat/om1_detached_porosity.pdf]

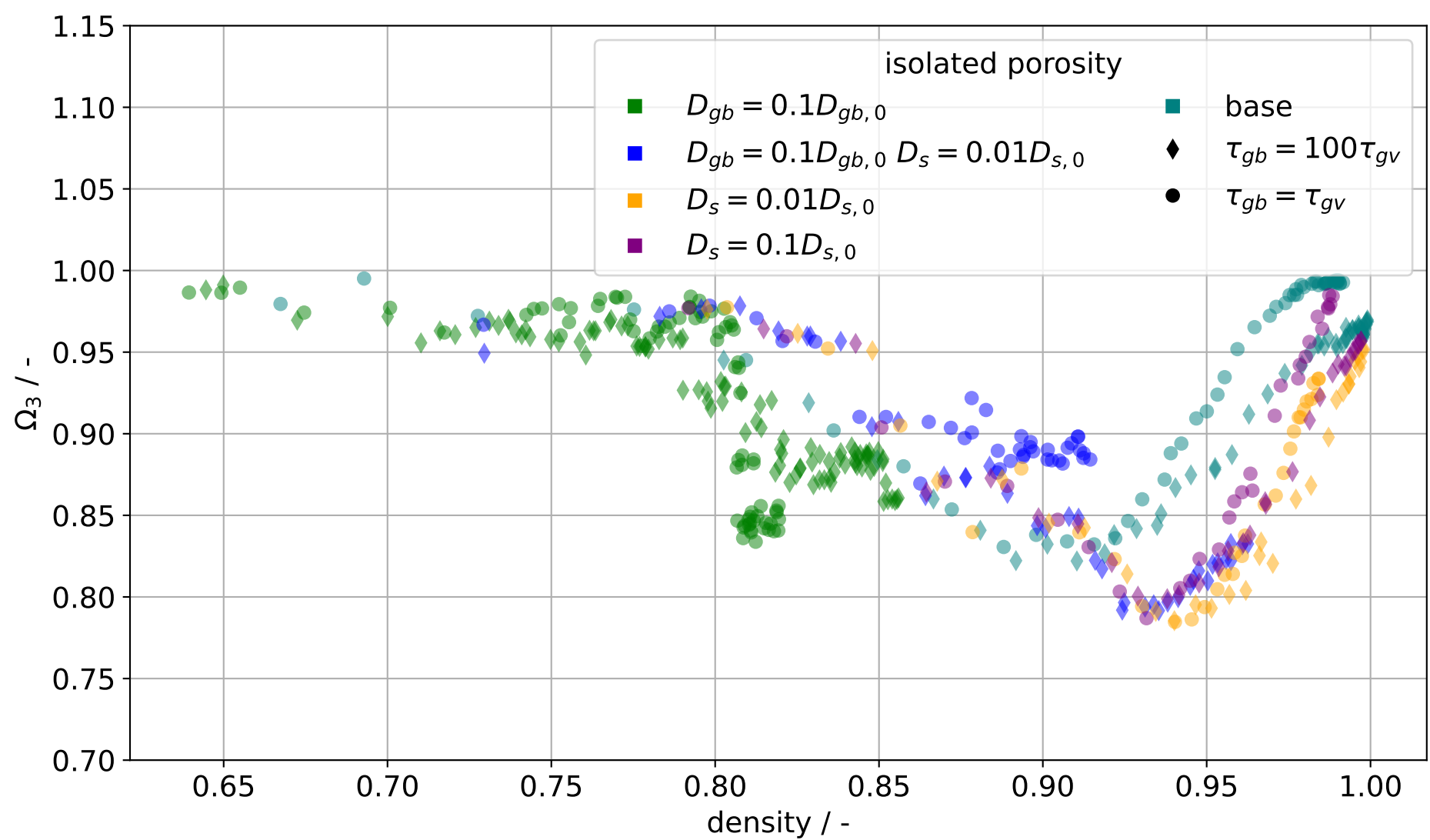

Supplement: Supplementary file 1 — Supplementary Information. [file 41598_2024_51915_MOESM1_ESM.zip › supmat/om3_isolated_porosity.pdf]

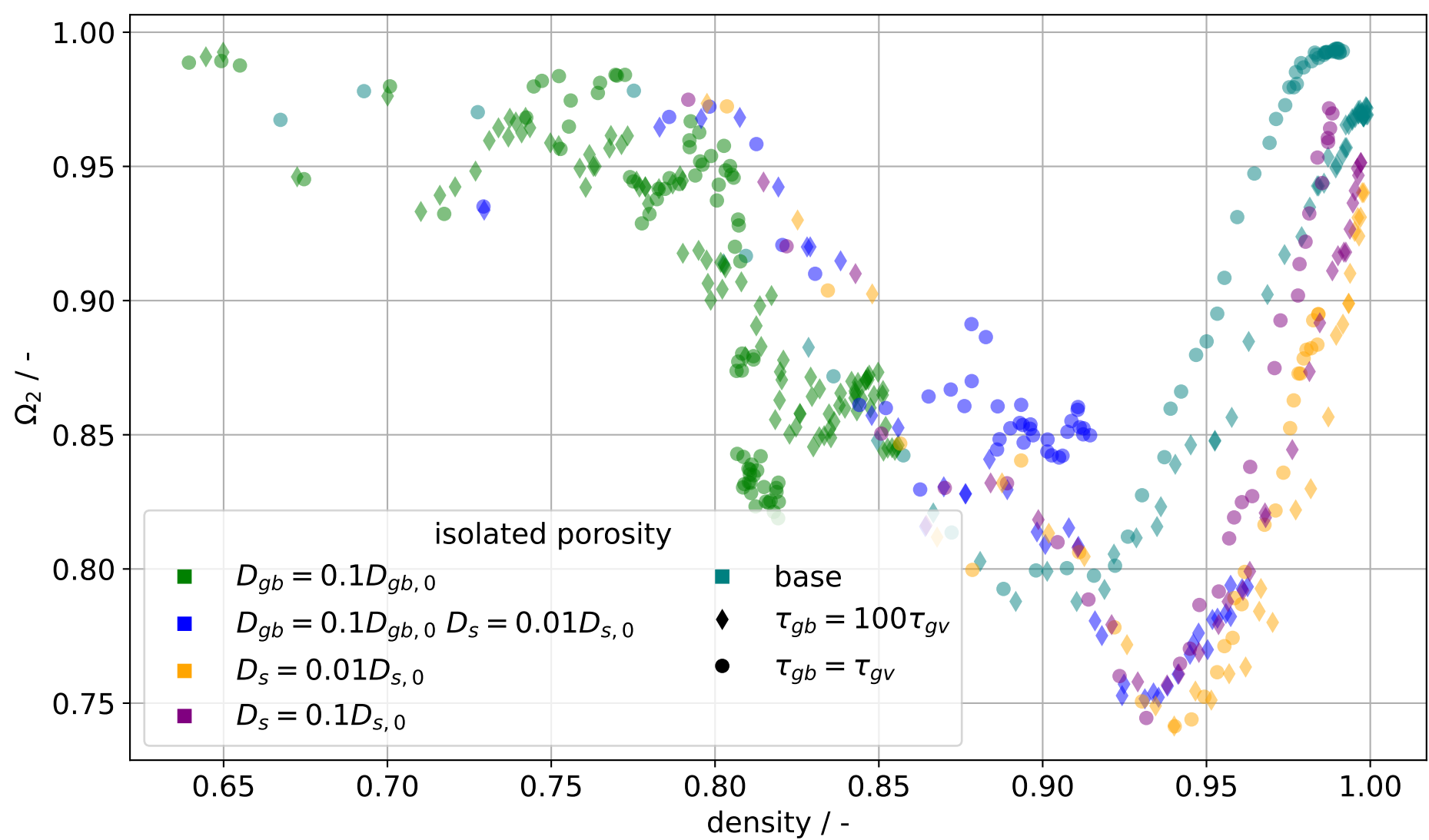

Supplement: Supplementary file 1 — Supplementary Information. [file 41598_2024_51915_MOESM1_ESM.zip › supmat/om2_isolated_porosity.pdf]

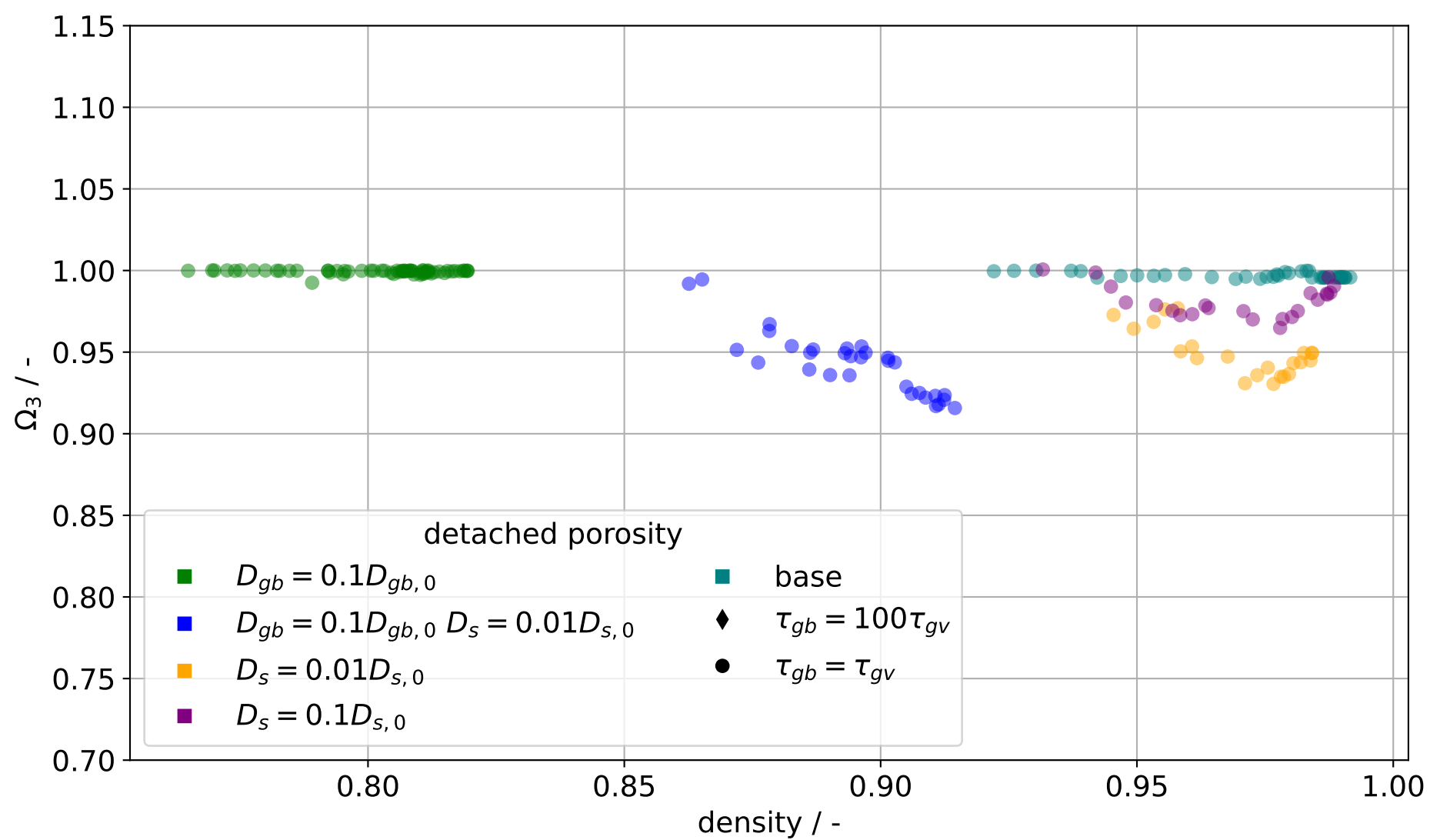

Supplement: Supplementary file 1 — Supplementary Information. [file 41598_2024_51915_MOESM1_ESM.zip › supmat/om3_detached_porosity.pdf]

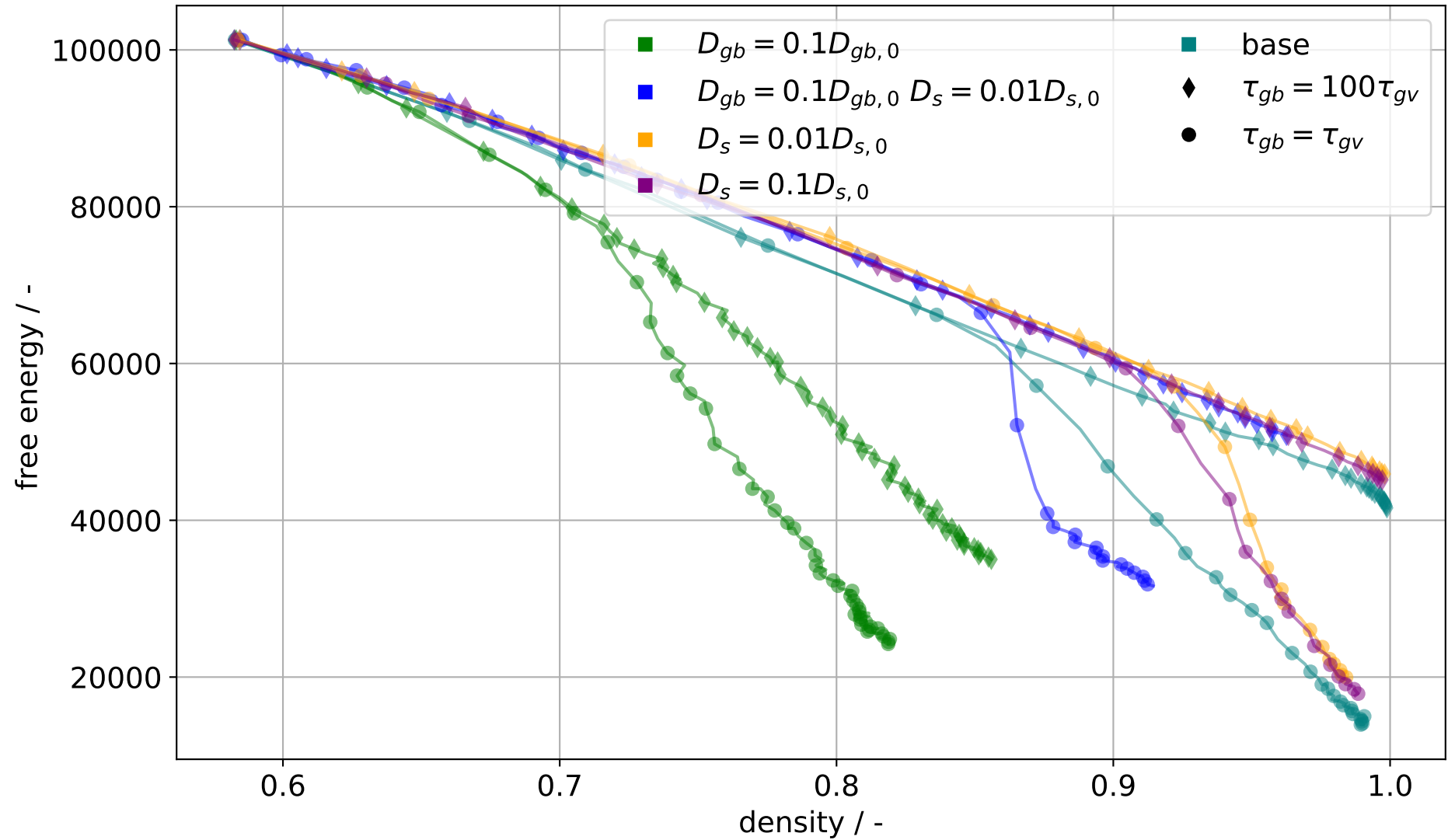

Supplement: Supplementary file 1 — Supplementary Information. [file 41598_2024_51915_MOESM1_ESM.zip › supmat/energy.pdf]

bulk viscosity  $K$  / MPas

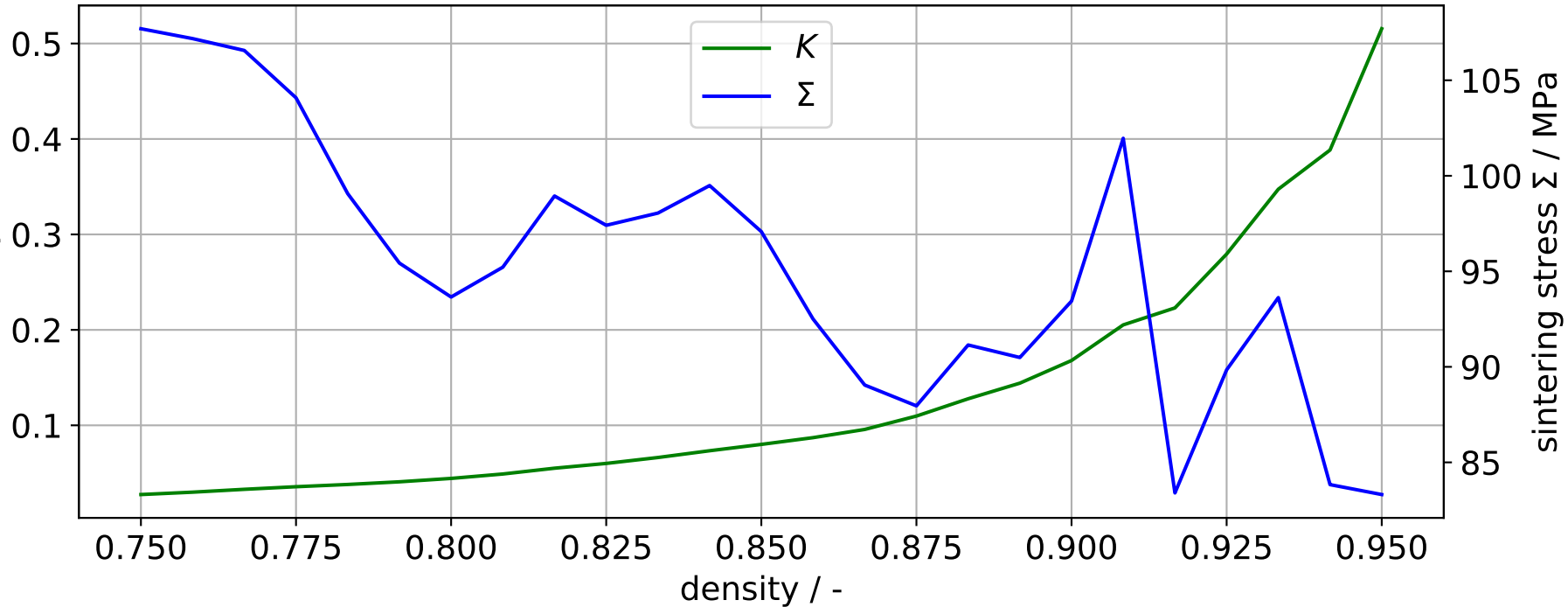

Supplement: Supplementary file 1 — Supplementary Information. [file 41598_2024_51915_MOESM1_ESM.zip › supmat/viscmodel.pdf]
